# Unveiling high solifuge diversity: Review of the genus *Pseudocleobis* Pocock, 1900 (Ammotrechidae) in Chile with the description of nine new species

**DOI:** 10.1371/journal.pone.0309776

**Published:** 2025-01-15

**Authors:** Hernán Augusto Iuri, Andrés Alejandro Ojanguren-Affilastro, Emilio A. Maury, Fermín M. Alfaro, Bernardino Camousseigt-Montolivo, Jaime Pizarro-Araya

**Affiliations:** 1 División Aracnología, Museo Argentino de Ciencias Naturales Bernardino Rivadavia, Capital Federal, Buenos Aires, Argentina; 2 Laboratorio de Entomología Ecológica, Departamento de Biología, Facultad de Ciencias, Universidad de La Serena, La Serena, Chile; 3 Programa de Doctorado en Conservación y Gestión de la Biodiversidad, Facultad de Ciencias, Universidad Santo Tomás, Santiago, Chile; 4 Programa de Doctorado en Biología y Ecología Aplicada, Universidad Católica del Norte, Universidad de La Serena, La Serena, Chile; 5 Environment & Permitting ‐ HSEQ, Enel Green Power & Thermal Generation, Santiago, Chile; 6 Instituto de Ecología y Biodiversidad (IEB), Santiago, Chile; 7 Grupo de Artrópodos, Sistema Integrado de Monitoreo y Evaluación de Ecosistemas Forestales Nativos (SIMEF), Santiago, Chile; Laboratoire de Biologie du Développement de Villefranche-sur-Mer, FRANCE

## Abstract

The *Pseudocleobis* from Chile are revised. *Pseudocleobis morsicans* (Gervais, 1849) and *P. chilensis* Roewer, 1934 are considered *species inquerenda. Pseudocleobis andinus* (Pocock, 1899) is removed from the Chilean fauna, and its previous records are considered misidentifications. *Pseudocleobis alticola* Pocock, 1900 is recorded from Chile for the first time. Nine new species are described, *Pseudocleobis elongatus* n. sp., *P. atacamensis* n. sp., *P. puna* n. sp., *P. krausi* n. sp., *P. choros* n. sp., *P. lalackama* n. sp., *P. mumai* n. sp., *P. cekalovici* n. sp. and *P. escuadra* n. sp. The species can be distinguished by the male chelicera morphology. We discuss the relationship of Chilean *Pseudocleobis* with other species of the genus, based on morphology. This work increases the number of known Chilean solifuge species by almost 70%, revealing the high degree of diversification of this group in Chile.

## Introduction

**Fig 1:**
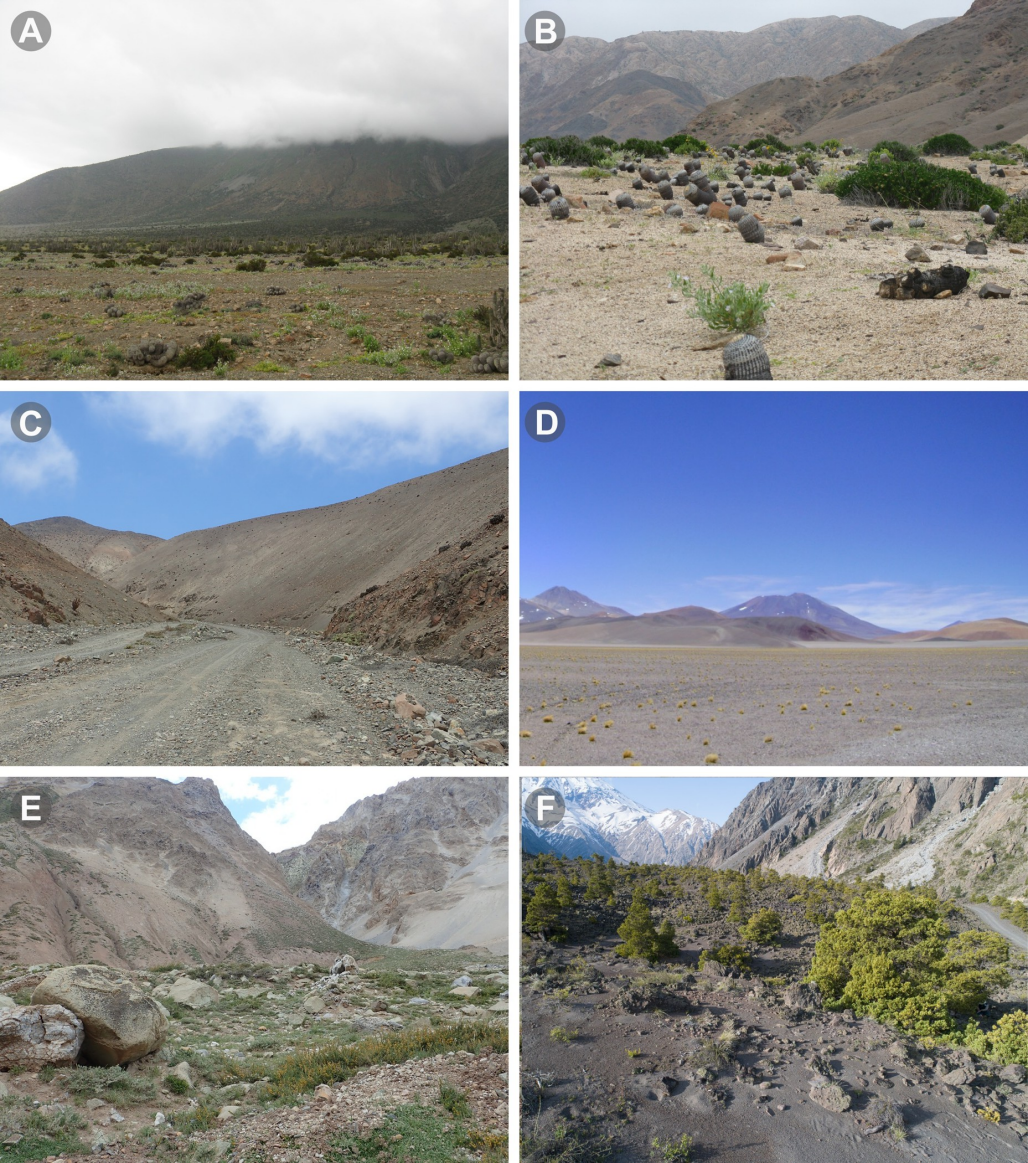
Habitat of some *Pseudocleobis* Pocock, 1900 in Chile. (A) Paposo, Antofagasta region (Coastal Desert enviroment). (B) Pan de Azúcar national park, Atacama region (Coastal Desert enviroment). (C) Caleta El Cobre, Antofagasta region (Coastal Desert-Absolute Desert ecotone). (D) Nevados Tres Cruces national park, near Paso San Francisco, Atacama region (High Andean environment). (E). Rivera Río Volcán, Metropolitan región (Central Andes environment). (F) Fundo La Escuadra, Maule region (Central Andes environment).

**Fig 2:**
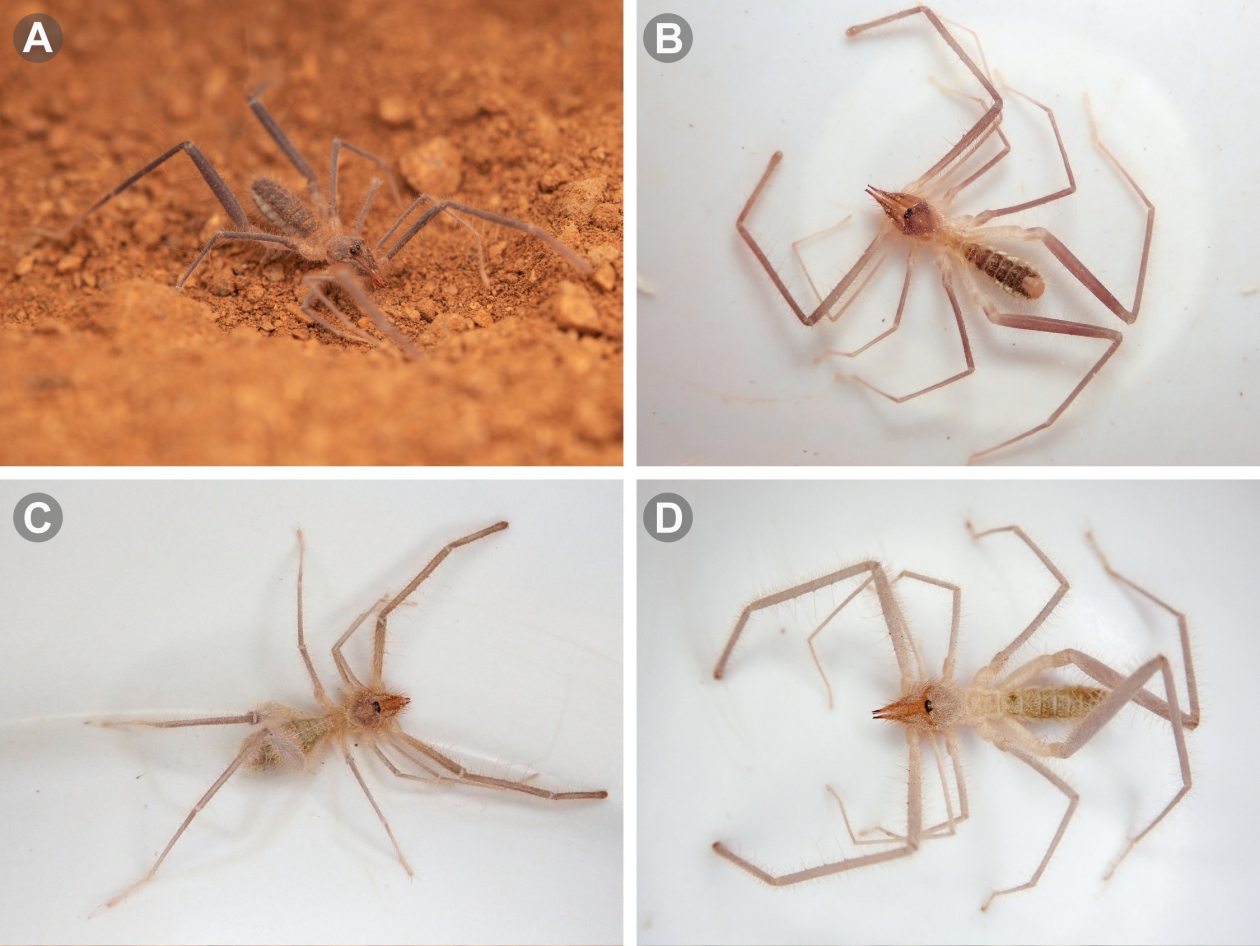
Live male specimens of some Chilean *Pseudocleobis* species. (A) *Pseudocleobis lalackama* n. **sp.** (B) *Pseudocleobis elongatus* n. **sp.** (C) *Pseudocleobis krausi* n. **sp.** (D) *Pseudocleobis atacamensis* n. **sp**.

**Fig 3:**
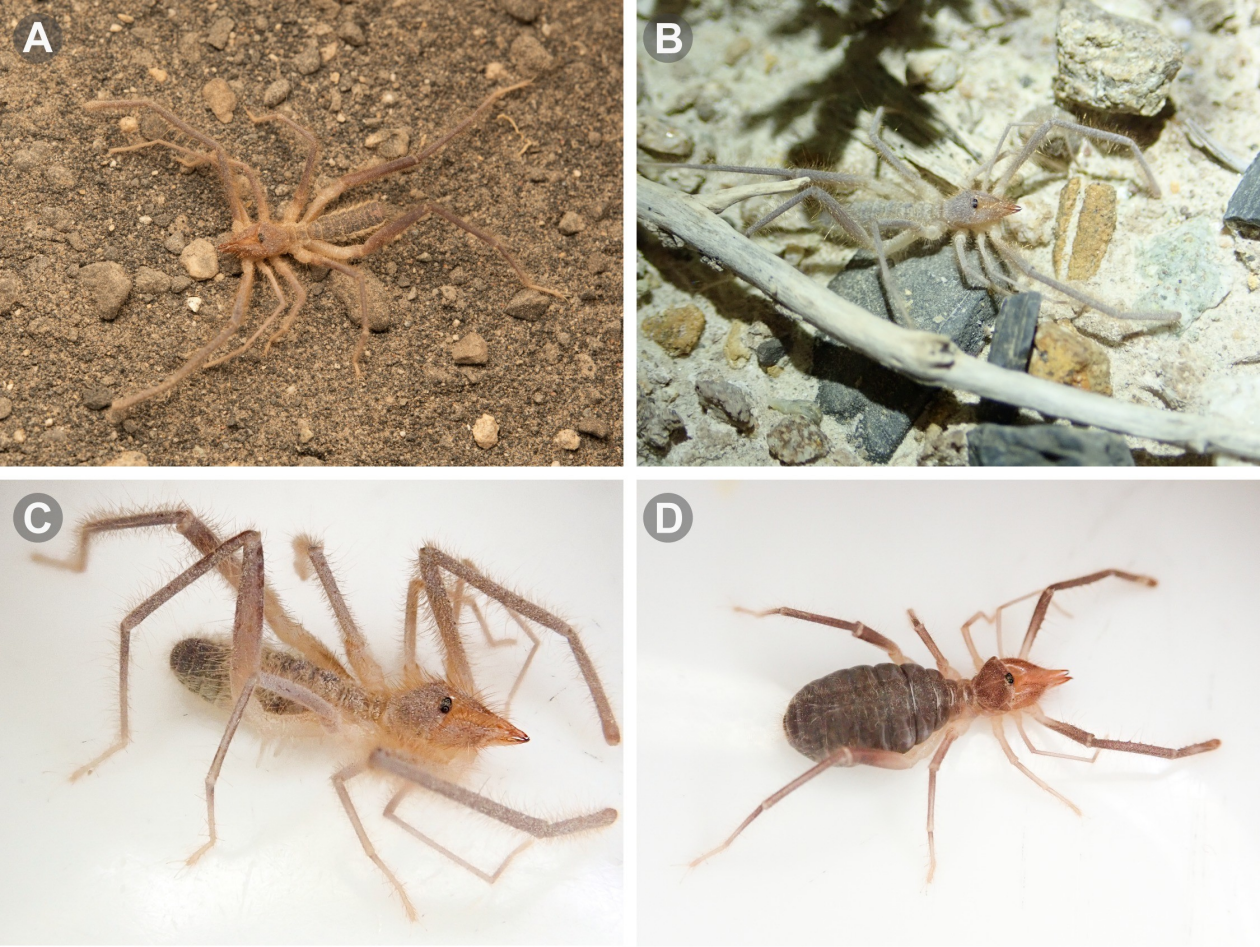
Live specimens of some Chilean *Pseudocleobis* species. (A) *Pseudocleobis escuadra* n. **sp.**, male. (B-C) *Pseudocleobis cakalovici* n. **sp.**, male. (D) *Pseudocleobis cakalovici* n. **sp.**, female.

The solifuge family Ammotrechidae Roewer, 1934 is endemic to the New World and currently comprises 24 genera and 101 species [1–3]. The genus *Pseudocleobis* Pocock, 1900 is the most diverse within Ammotrechidae, currently with 22 valid species [2] distributed in Argentina (13 spp), Bolivia (3 spp), Chile (3 spp) and Perú (6 spp). This genus occurs in arid and semiarid environments from Southern South America (Fig 1), and comprises small- to medium-sized species (Figs 2 and 3) with nocturnal habits. It includes lowland species as well as high Andean species, with some of the highest elevational records of the order [4].

Gervais [5] described the species *Galeodes morsicans* Gervais, 1849 from Chile. The species was later transferred to *Pseudocleobis* by Kraepelin [6]. Roewer [7] described *Pseudocleobis chilensis* Roewer, 1934 and recorded *Pseudocleobis andinus* (Pocock, 1899) from Chile. Kraus [8] added several records of *P. andinus* from Chile. Muma [9] provided some records of *Pseudocleobis* from Chile that he considered as *P. andinus*, given that he couldn’t place the specimens through Roewer’s (1934) diagnoses.

Maury published several contributions on *Pseudocleobis*, but all of them were focused on Argentine species [10–12]. Among these contributions, he clarified the identity of *P. andinus*, the type species of the genus [12]. Whilst he never published on Chilean *Pseudocleobis*, we found several unpublished notes at the “Museo Argentino de Ciencias Naturales” (MACN) regarding the Chilean *Pseudocleobis*, including descriptions and drawings of previous records from Chile made by Roewer, Kraus, and Muma. The examination of these notes, along with the examination of several specimens of *Pseudocleobis* from Chile, belonging to different collections and newly collected materials, let us to identify the described Chilean species of the genus, as well as to the recognition of nine new species, which are described in this contribution. Due to this we decided to include the late Dr. Emilio A. Maury (^**†**^ MACN) as one of the authors of this contribution, in recognition of his contribution to the preliminary definition of the taxonomic and morphological limits of some *Pseudocleobis* species from Chile which were fundamental for this contribution.

## Materials and methods

### Studied material

Fieldwork was performed during 2006–2023 on several localities from Antofagasta, Atacama, Coquimbo, Valparaiso, Metropolitan and Maule regions. Specimens were collected using pitfall traps, or manually by searching on the ground at night with a headlight. The type of *P. chilensis*, as well as most of the Muma’s records of *P. andinus* from Chile were examined. Other specimens recorded by Muma, and the specimens recorded by Kraus were examined by Maury, from which we revised unpublished notes, drawings and descriptions now deposited at the MACN. Additionally, materials from other collections were also examined. Collections abreviations: AMNH: American Museum of Natural History, New York, USA (Lorenzo Prendini); LEULS: Laboratorio de Entomología Ecológica, Universidad de La Serena, La Serena, Chile (Jaime Pizarro-Araya); MACN: Museo Argentino de Ciencias Naturales “Bernardino Rivadavia”, Buenos Aires, Argentina (Martín J. Ramírez); MCZ: Museum of Comparative Zoology, Harvard, Massachusetts, USA (Gonzalo Giribet); MNHN: Museum Nationale d’Histoire Naturelle, París, France (Mark Judson); MNNC: Museo Nacional de Historia Natural, Santiago, Chile (Mario Elgueta Donoso); MNRJ: Museo Nacional de Río de Janeiro, Río de Janeiro, Brasil (Adriano Kury); MZUC-UCCC: Museo de Zoología de la Universidad de Concepción, Concepción, Chile (Laura Tavera Martínez); SMF: Senckenberg Museum Frankfurt, Frankfurt, Germany (Peter Jäger); ZMH Zoologischer Museum Hamburg, Hamburg, Germany (Danilo Harms). Other specimens of *Pseudocleobis* from Argentina, Bolivia and Perú were examined only for comparative purposes since they are not within the scope of this work.

### Fieldwork permissions

The authorization to collect was provided by Corporación Nacional Forestal, Chile (CONAF), with the project’s numbers 18/2011, 006/2014, 028/2015, 053/2015, 008/2017 (CONAF-SIMEF), 056/2017 (CONAF-SIMEF) 85/2019 (CONAF-SIMEF) 44/2022 (CONAF-SIMEF), 045/2022 (CONAF-SIMEF) and 04/2023 (CONAF-SIMEF).

### Taxonomic methodology

Measurements, taken using an ocular micrometer, are recorded in mm. Cheliceral terminology follows Bird et al. [13]. Pedipalp chaetotaxy terminology follows Cushing & Casto [14]. Leg segmentations follow Shultz [15]. Terminology used for leg metatarsal spiniform setal patterns follows Iuri & Iglesias [2]. Abbreviations: FD, fixed finger, distal tooth; FM, fixed finger, medial tooth; FP, fixed finger, proximal tooth; FSD, fixed finger, subdistal tooth/teeth; FSM, fixed finger, submedial tooth/teeth; MM, movable finger, medial tooth; MP, movable finger, proximal tooth; MSM, movable finger, submedial tooth/teeth; PFM, fixed finger, profondal medial tooth; PFP, fixed finger, profondal proximal tooth; PFSM, fixed finger, profondal submedial tooth/teeth; PFSP, fixed finger, profondal subproximal tooth/teeth; RFM, fixed finger, retrofondal medial tooth; RFP, fixed finger, retrofondal proximal tooth; RFSM, fixed finger, retrofondal submedial tooth/teeth; RFSP, fixed finger, retrofondal subproximal tooth/teeth; *rlpc*, retrolateral proximal setal cluster.

The species are presented in the text based in the following criteria: first, species previously described and cited for Chile; second, new records of known species; last, the new species herein described. The new species are presented following morphological similarity, as they might comprise natural species groups, and in order to make interspecies comparisons easier.

The descriptions are based on the male holotype, and a female specimen (when known). Each description has a variability section where the variations observed on some discrete characters (e.g., spine-like setal patterns, dentition) are provided. The variability on measurements, the minimum (MIN), maximum (MAX), and mean values for body, propeltidium and chelicera are provided in table format, and based on the specimens available (up to an n = 10 in the case of the males of *P. cekalovici* **n. sp.**) (S1 Table).

### Microscopy and images

Illustrations were performed with Adobe Illustrator CC 2020 24.3 by superimposing vectors on previously obtained micrographs or drawings. Digital images of fixed specimens were taken at the MACN under visible light, using a digital camera (Leica DFC290) attached to a stereomicroscope (Leica M165C or Nikon SMZ1500), and the focal planes stacked with Helicon Focus 3.10.3 (http://helicon.com.usa/heliconfocus/). Digital images of live specimens were taken with an Olympus Though TG-5 (Figs 2B–2D and 3B–3D), and Nikon D850, equipped with a Laowa 100 mm macro lens (Figs 2A and 3A). Images were edited with Adobe Photoshop 21.2.4, Adobe Lightroom Classic v9.4 and Olympus Workspace v1.4.1. The distribution maps were produced using SimpleMappr [16]. The original polygons of phytogeographic areas from Gajardo [17] were downloaded from an online source [18] and converted to WKT format for use in SimpleMappr.

Scanning electron microscopic (SEM) images were taken with a Zeiss Gemini SEM 360 microscope at the MACN Electron Microscopy Laboratory. Prior to SEM imaging, the focal body parts were dissected and cleaned with ultrasonic cleaner and a thin brush. The dehydration of the pieces was carried out via 80%, 85%, 96%, 100% ethanol series; all samples were critical-point dried after dehydration in ethanol. A gold palladium coating was applied on a Termo VG Scientific SC 7620 mini sputter-coater.

### Nomenclatural acts

The electronic edition of this article conforms to the requirements of the amended International Code of Zoological Nomenclature, and hence the new names contained herein are available under that Code from the electronic edition of this article. This published work and the nomenclatural acts it contains have been registered in ZooBank, the online registration system for the ICZN. The ZooBank LSIDs (Life Science Identifiers) can be resolved and the associated information viewed through any standard web browser by appending the LSID to the prefix "http://zoobank.org/". The LSID for this publication is: urn:lsid:zoobank.org:pub:196C364E-784B-453A-A393-A1D9647F105F. The electronic edition of this work was published in a journal with an ISSN, and has been archived and is available from the following digital repositories: PubMed Central, LOCKSS and Consejo Nacional de Investigaciones Científicas y Técnicas, Argentina (CONICET).

## Results

### Taxonomy

***Pseudocleobis* Pocock, 1900.** *Pseudocleobis* Pocock, 1900 [19]: 304; Kraepelin, 1901 [6]: 108; Roewer, 1934 [7]: 602; Mello-Leitão, 1938 [20]: 23; Muma, 1971 [9]: 14–16; Muma, 1976 [21]: 28; Harvey, 2003 [22]: 206; Iuri & Iglesias, 2022 [2]: 440–441.

*Tetracleobis* Roewer, 1934 [7]: 605; Muma, 1976 [21]: 28 (synonymized by Maury [10]: 93).

**Type species.** *Cleobis andinus* Pocock, 1899, by original designation.

**Fig 4:**
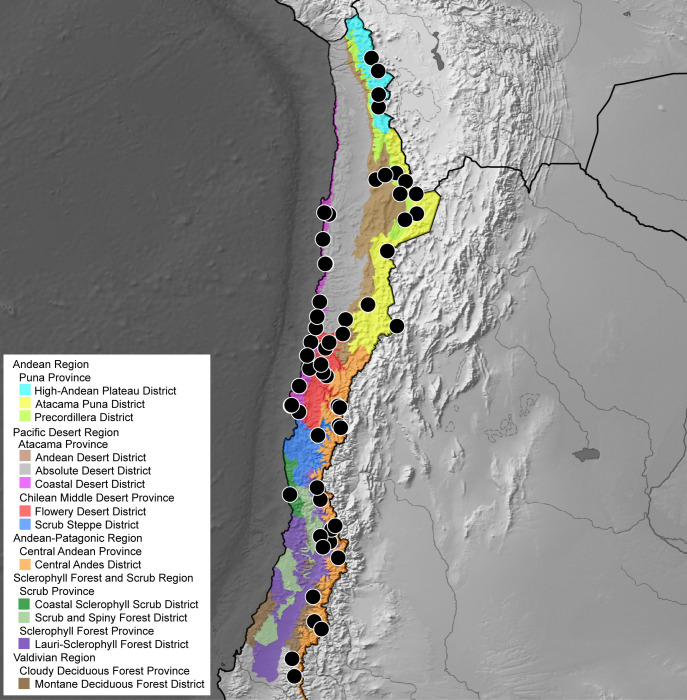
Confirmed records of the genus *Pseudocleobis* in Chile.

**Fig 5:**
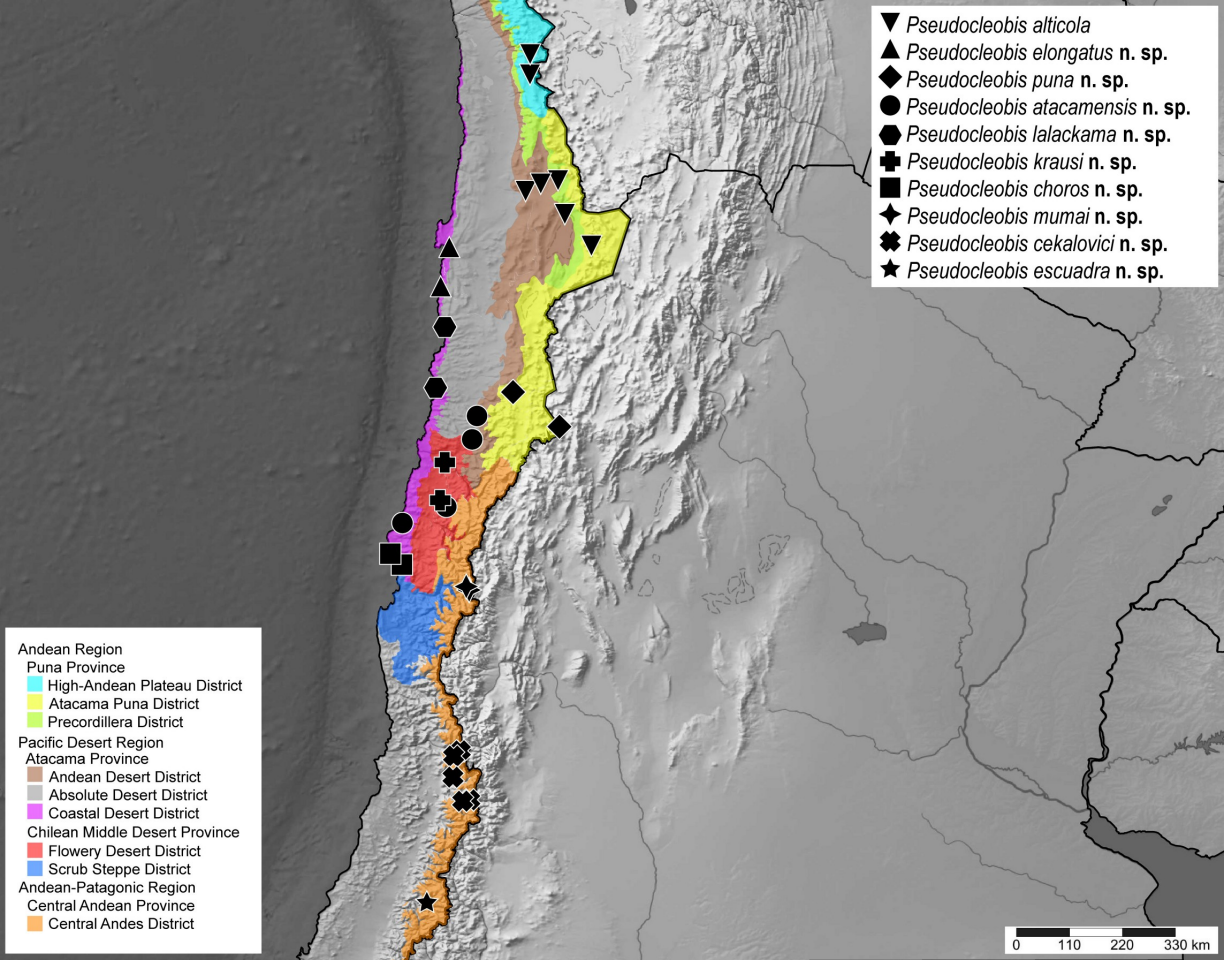
Records of *Pseudocleobis* species in Chile.

**Distribution in Chile.** The genus is distributed from the Arica and Parinacota region to Biobío region (Figs 4 and 5). In the northern part of the country (Arica and Parinacota, Tarapacá, Antofagasta, Atacama and Coquimbo regions), the genus is distributed in both the Andes and coastal deserts, but in the central and southern part of the country (from Santiago downwards) the genus seems to be restricted to the high altitudes of the Andes Mountain chain (Figs 4 and 5).

**Fig 6:**
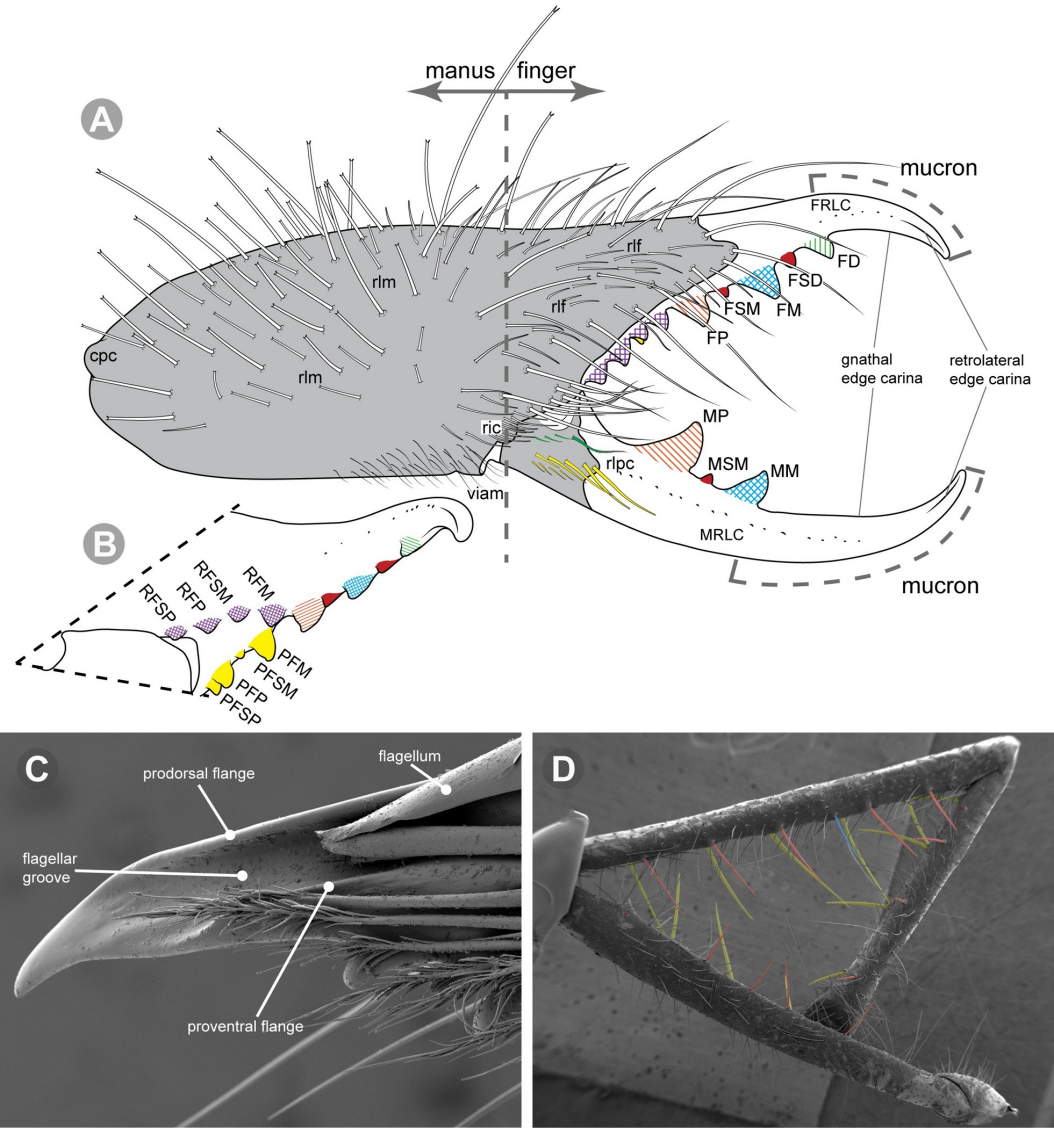
*Pseudocleobis* general morphology. (A) *Pseudocleobis elongatus* **n. sp.**, scheme of female chelicera, retrolateral view. (B) *Pseudocleobis chilensis* Roewer, 1934, fixed finger, retroventral view. (C) *Pseudocleobis alticola* Pocock, 1900, SEM images of male fixed finger mucron, prolateral view. (D) *Pseudocleobis lalackama* **n. sp.**, right pedipalp, retrolateral view (retrolateral spine-like setae in red [main] and blue [supplementary], prolateral in yellow; arrow indicates supplementary spine-like setae). Abbreviations: *cpc*, cheliceropeltidial condyle; FD, fixed finger, distal tooth; FM, fixed finger, medial tooth; FP, fixed finger, proximal tooth; FRLC, fixed finger retrolateral carina; FSD, fixed finger, subdistal tooth/teeth; FSM, fixed finger, submedial tooth/teeth; MM, movable finger, medial tooth; MP, movable finger, proximal tooth; MRLC, movable finger retrolateral carina; MSM, movable finger, submedial tooth/teeth; PFM, profundal medial tooth; PFP, profundal proximal tooth; PFSM, profundal submedial tooth; PFSP, profundal subproximal tooth; RFM, retrofondal medial tooth; RFP, retrofondal proximal tooth; RFSM, retrofondal submedial tooth/teeth; RFSP, retrofondal subproximal tooth/teeth; *ric*, retrolateral interdigital condyle; *rlf*, retrolateral finger setae; *rlm*, retrolateral manus setae; *rlpc*, retrolateral proximal setal cluster (dorsal cluster in green, ventral cluster in yellow); *viam*, ventral interdigital articular membrane.

**Fig 7:**
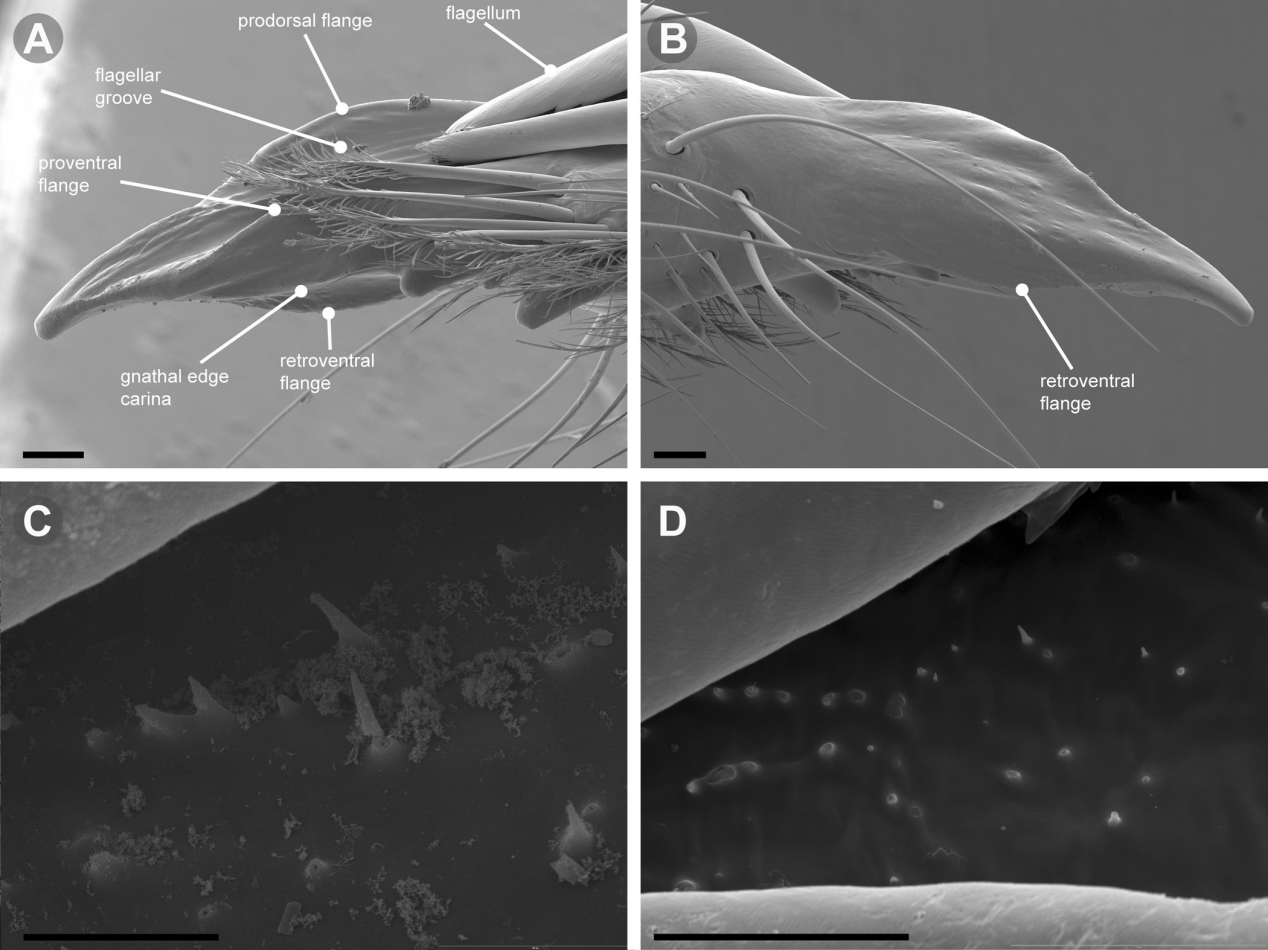
*Pseudocleobis andinus* (Pocock, 1899), male right chelicera. (A) Fixed finger mucron proventral view. (B) Fixed finger, retrolateral view. (C–D) Flagellum, detail of distal prolateral surface. Scale bars: 01 mm (A–B); 0,05 (D); 0,02 mm (C).

**Fig 8:**
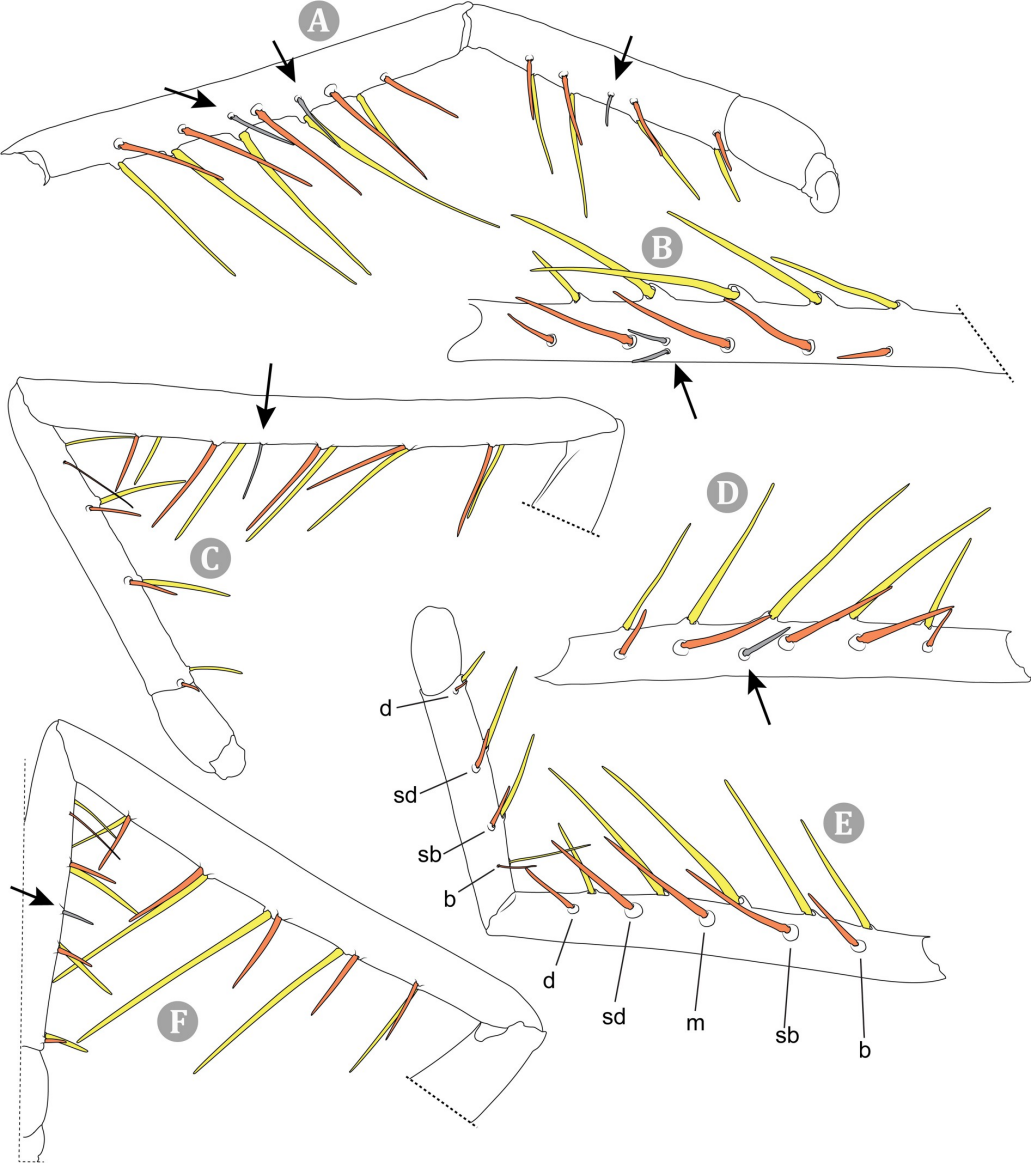
General spiniform setal pattern and some variability observed in pedipalps of Chilean *Pseudocleobis*. Prolateral spiniform setae in yellow, retrolateral spiniform seta in red, accessory spiniform setae in grey; arrows indicate the accessory spiniform setae. (A) *Pseudocleobis escuadra* n. sp., female, right pedipalp, retroventral view. (B) *Pseudocleobis choros* n. sp., female, right pedipalp, retroventral view. (C) *Pseudocleobis choros* n. sp., male, left pedipalp, lateral view. (D) *Pseudocleobis krausi* n. sp., hembra, left pedipalp, retroventral view. (E) *Pseudocleobis krausi* n. sp., female, right pedipalp, retroventral view. (F) *Pseudocleobis elongatus* n. sp., female, left pedipalp, lateral view. d: distal; sd: subdistal; m: medial; sb: subbasal; b: basal.

**Generalities on Chilean species.** The Chilean *Pseudocleobis* exhibits all the generic traits as described in Iuri & Iglesias [2]. The whole body with bifurcated tip setae. Cheliceral fixed finger with FD, FSD, FM, FSM and FP teeth on the median series (Fig 6A); with RFM, RFSM, RFP, RFSP teeth on the retrofondal series (Fig 6A) and with PFM, PFSM, PFP, PFSP teeth on the profondal series (Fig 6B); the distalmost RF tooth is more distal relative to the PFM tooth (as occur in most species of this genus), which might suggest considering it as RFA, however, as Bird et al [13] defined, the RFA is more aligned with the median series and is more similar in shape to the teeth of the median series, none of which occur here (the tooth has a similar triangular shape as the other RF teeth, and is not a part of the linear progression of the median series), so we consider it as a displaced RFM tooth. Movable finger with MM, MSM, MP teeth (Fig 6A), without MPL tooth; dorsal cluster of *rlpc* with more than one seta (Fig 6A). Females and juveniles without dorsal hump on fixed finger. Fixed finger of males highly modified including a conspicuous flagellar groove with well-defined prodorsal and proventral flange (Figs 6C and 7A); a retroventral flange (Fig 7B) is present in some species. Flagellum base as translucent, pear-shaped (concave to the apex) or teardrop-shaped (straight or convex to the apex) membrane; the prolateral surface might be smooth or with some projections (Fig 7C and 7D) on the distal half. Pedipalp with long, paired lateroventral spiniform setae (Figs 6D and 8). Tibia II and III without dorsoapical spiniform seta. Basitarsus II and III with two retrolateral (i.e., RL-b, RL-sd), one retrodorsal distal (i.e., RD-d), two proventral (i.e., PV-sd, PV-d) and one retroventral distal (i.e., RV-d) spiniform setae. Telotarsus II and III divided into two tarsomeres with the following spiniform setal formula 1.2.2/2.2. Telotarsus IV divided into four tarsomeres with the following spiniform setal formula 2.2-2-2/2.2. Arolium very small.

The filiform ctenidia on sternites III and IV are present in the Chilean species, in a similar pattern as shown for the genus in previous works [1, 2], although the number seems to be intra-specifically variable.

The pedipalps of *Pseudocleobis* species exhibit four lateroventral paired spiniform setae on the basitarsus (herein referred by their respective position as basal, subbasal, subdistal and distal) and five pairs on the tibia (herein referred by their respective position as basal, subbasal, medial, subdistal and distal; Fig 8E), usually being more spiniform in females than in males. In many Chilean species the proximal pair of spiniform setae of the basitarsus (and sometimes in tibia) is weaker, being more setiform (Fig 8C, 8E and 8F), especially in males, giving the appearance of having only three pairs of spiniform setae on the basitarsus (and four pairs on the tibia); in rare cases one or both of the spiniform seta of the basal pair of the basitarsus may appear completely replaced by a bifurcated tip seta, and this may occur in only one of the pedipalps, or both. Additional spiniform setae (Figs 6D and 8A), beside the normal pattern, are frequent in some of the Chilean species. For example, a small accessory retroventral spiniform seta between the medial and the subdistal spiniform setae of the tibia (Figs 6D and 8C) appears in *P. lalackama* n. sp., *P. choros* n. sp., *P. cekalovici* n. sp. and *P. escuadra* n. sp.; all of which also share similarities in male cheliceral morphology. *Pseudocleobis escuadra* n. sp. also bears a small accessory retroventral spiniform seta between the subbasal and the medial (Fig 8A) spiniform setae of the tibia (in males and females), and an additional retroventral one between the subbasal and subdistal (observed only in females). Anomalous spiniform setae have been observed also (Fig 8B, 8D and 8F), and they were identified when occurring in one of the pedipalps of the focal specimen. A similar pattern occurs in the femur, where the proventral series seems to have five lateroventral spiniform setae, but in males the basal one is usually setiform. In the retroventral series there are five spiniform setae imperfectly paired with the retroventral; additional smaller spiniform setae intercalated, and imperfectly aligned, may also be present, especially in females, raising the number to 6 or 7. On the other hand, some males only show four retroventral spiniform setae, the rest being setiform and indistinguishable from the normal setae.

The color in alcohol is usually brown dorsally, and pale ventrally; however, there are some variations, with some individuals being paler than others (Figs 2 and 3). It’s common to see live specimens, especially males, with paler coloration, but this seems to be an artifact of the dirt and sand sticking between the hairs (males are usually hairier than females), as they turn brown when put in alcohol. As in other species of this genus the coxae and trochanter of the legs and pedipalps have no pigmentation.

**Species composition in Chile:** *Pseudocleobis alticola* Pocock, 1900, *P. elongatus* n. sp., *P. atacamensis* n. sp., *P. puna* n. sp., *P. krausi* n. sp., *P. choros* n. sp., *P. lalackama* n. sp., *P. mumai* n. sp., *P. ceckalovici* n. sp., *P. escuadra* n. sp.

*Species inquirenda*: *P. morsicans* (Gervais, 1849), *P. chilensis* Roewer, 1934.

**Note:** Although the type locality of *P. andin*us is in the Andes, close to the Argentina-Chile border, no specimens of this species are known from Chile, and all the previous records were misidentifications of different new species herein described.

***Pseudocleobis morsicans* (Gervais, 1849) species *inquirenda. Galeodes morsicans*** Gervais, 1849 [5]: 17–16, Fig 1A–1E; Putnam, 1883 [23]: 269.

*Gluvia morsicans* (Gervais): Butler, 1873 [24]: 424.

*Cleobis morsicans* (Gervais): Simon, 1879 [25]: 150.

*Pseudocleobis morsicans* (Gervais): Kraepelin, 1901[6]: 109; Roewer, 1934 [7] (Part): 605, figs 336g, 341c; Mello-Leitão, 1938 [20]: 25, fig. 57; Mello-Leitão, 1939 [26]: 614; Zilch, 1946 [27]: 152; Cloudsley-Thompson, 1968 [28]: 110; Muma, 1971 [9]: 16–17, Figs 29–30; Cekalovic, 1975 [29]: 133–134; Muma, 1976 [21]: 28; Cloudsley-Thompson, 1977 [30]: 64; Cloudsley-Thompson, 1978 [31]: 186; Maury, 1998 [4]: 568.

**Type material (not located).** Types (1 male, 1 female and 1 juvenile? See notes below) from “provincias centrales”, Chile; MNHN N° 9100 (?).

**Notes.** *Galeodes morsicans* was described by Gervais [5] based on several specimens from Chile, “central provinces”; the work did not provide any information about the type depositories. The species was mentioned in further works, e.g., Simon [25], Putnam [23], Kraepelin [6], but none of them mentions having seen the types nor said anything about their location. Roewer [7] is the only researcher who claims to have revised the types, as he states “vidi 3 ♂, 4 ♀, 1 pullus, inkl. Typus”. The material studied by Roewer [7] is from Chile, Bolivia (Oruro) and Argentina (S. Lorenzo, Las Cuevas, Mendoza, Punta de Vacas), however the records from Argentina and Bolivia belong to other *Pseudocleobis* species.

According to Zilch [27] the Argentinian specimens are in the SMF and correspond to 1 ♂ from Las Cuevas and 2 ♂ from S. Lorenzo/Salta. Maury examined these specimens and stated that they are 1 ♂ and 2 ♀ placed in the same vial, and the label has the two localities. He states that the male is *P. alticola* and the females are *P. andinus*, this may suggest that the male is from San Lorenzo (Salta province) where *P. alticola* is common, and the two females are from Las Cuevas (Mendoza province), where *P. andinus* is commonly found.

The specimens from Bolivia (Oruro) are at the ZMH. According to Maury’s unpublished notes, they are 1 ♂ and 1 ♀ of *P. alticola*. The catalog of the ZMH by Harms & Dupérré [32] mentioned these specimens as *P. alticola* (see Harms & Dupérré [32], page 55, appendix 1).

Thus, the only known specimens of *P. morsicans* are the types of the species, and it can be deduced from Roewer [7] that the type material from Chile should be 1 ♂, 1 ♀ and 1 juvenile. The only type reference about its depository is given by Muma [21] who states “The type (*Galeodes morsicans*, sex?) from Chile, no. 9100 is in the MNHN.”. The question mark about sex and number of specimens suggests that Muma couldn’t revise the types. Maury visited the MNHN collection, and examined many types, however, in his unpublished notes, made it clear that he couldn’t find the type of *P. morsicans*.

Roewer’s [7] key to *Pseudocleobis* species, distinguished *P. morsicans* from other species of the genus, in part by the flagellum that extends beyond the FD tooth. This trait is not common in Chilean *Pseudocleobis*, as in most species the flagellum barely reaches the FD tooth; however, we found at least two species where the flagellum extends beyond the FD tooth.

The type locality: “central provinces” (of Chile), is extremely vague, especially while considering the historical context, since the description of *P. morsicans* dates from 1849, and the Chilean boundaries extended dramatically in 1884 after the Pacific War, thus making the term “central” even vaguer.

Therefore, since the type material could not be located, the descriptions available are insufficient to recognize the species, and the type locality is highly imprecise, we consider it currently as *species inquirenda*. Given that the type material includes a male, we consider that the identity of this species could be clarified only if the types are found.

There are two clues about the identity and depository of the types: one is given by Roewer [7] who claimed to have studied the specimens, and from whom it can be deduced that these are 1 ♂, 1 ♀ and 1 juvenile; the other one is given by Muma [21] who provides the collection and number of the types as MNHN 9100. The types of *Mummucia variegata* (Gervais, 1849), described by Gervais in the same work [5], are deposited in the same collection, suggesting that the MNHN is also the depository of the type material of *P. morsicans*. Additionally, as part of the same main comprehensive work, Nicolet [33] described many spiders, not specifying type material nor depository for them; most of these specimens, long-time considered lost, were found at the MNHN [34], thus providing further evidence that the remaining arachnological material from that work should also be deposited at the MNHN. We were not able to revise the MNHN collection, nor have we obtained any institutional information about this material. Due to this we consider that this material should be searched at the MNHN (and secondarily at the SMF, where most of the material studied by Roewer is housed) before considering the types as definitely lost.

**Fig 9:**
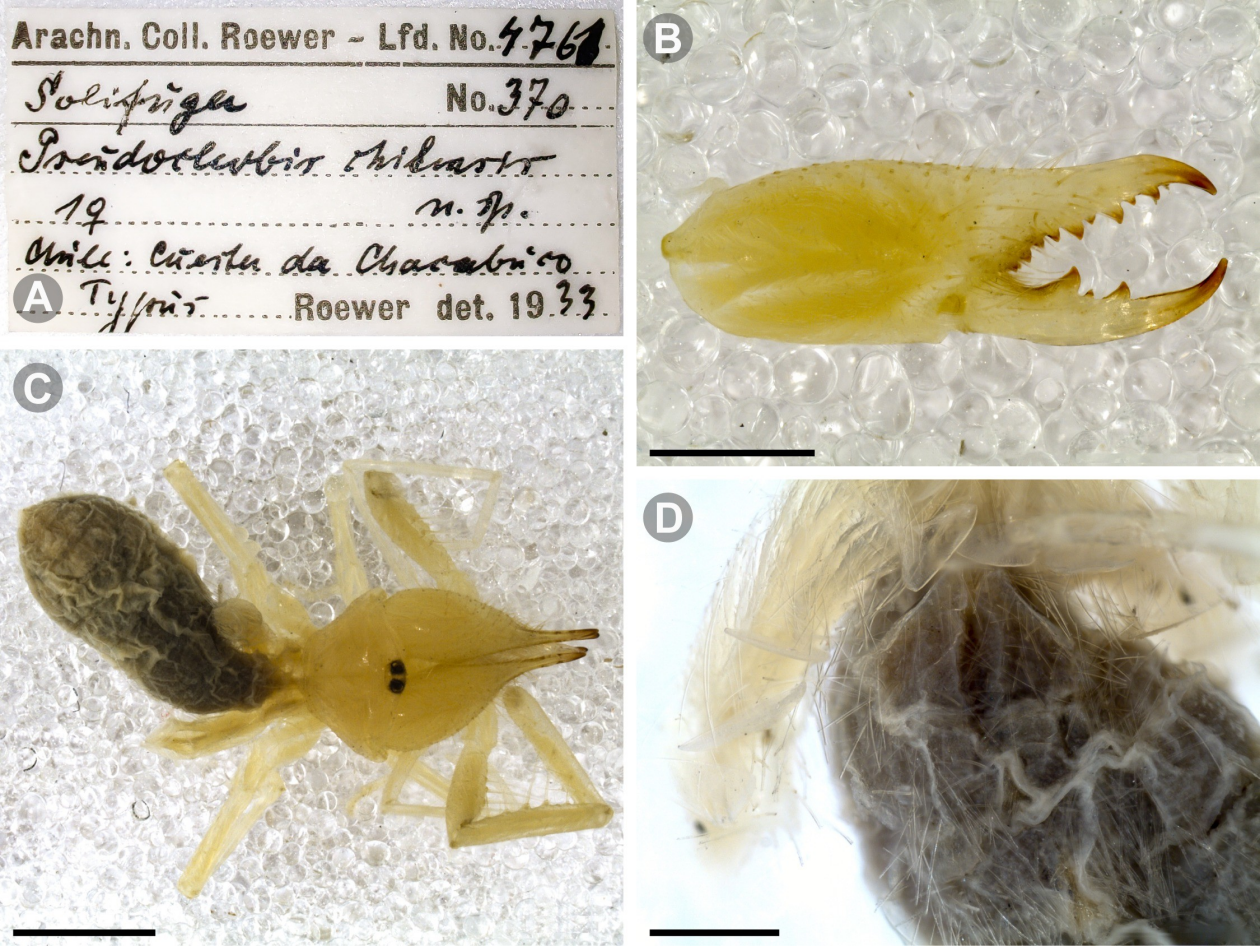
*Pseudocleobis chilensis* Roewer, 1934 holotype. (A) Specimen label. (B) Habitus, dorsal view. (C) Right chelicera, retrolateral view. (D) Genital sternite, ventral view. Scale bars: 2 mm (C); 1 mm (B); 0,5 mm (D).

**Fig 10:**
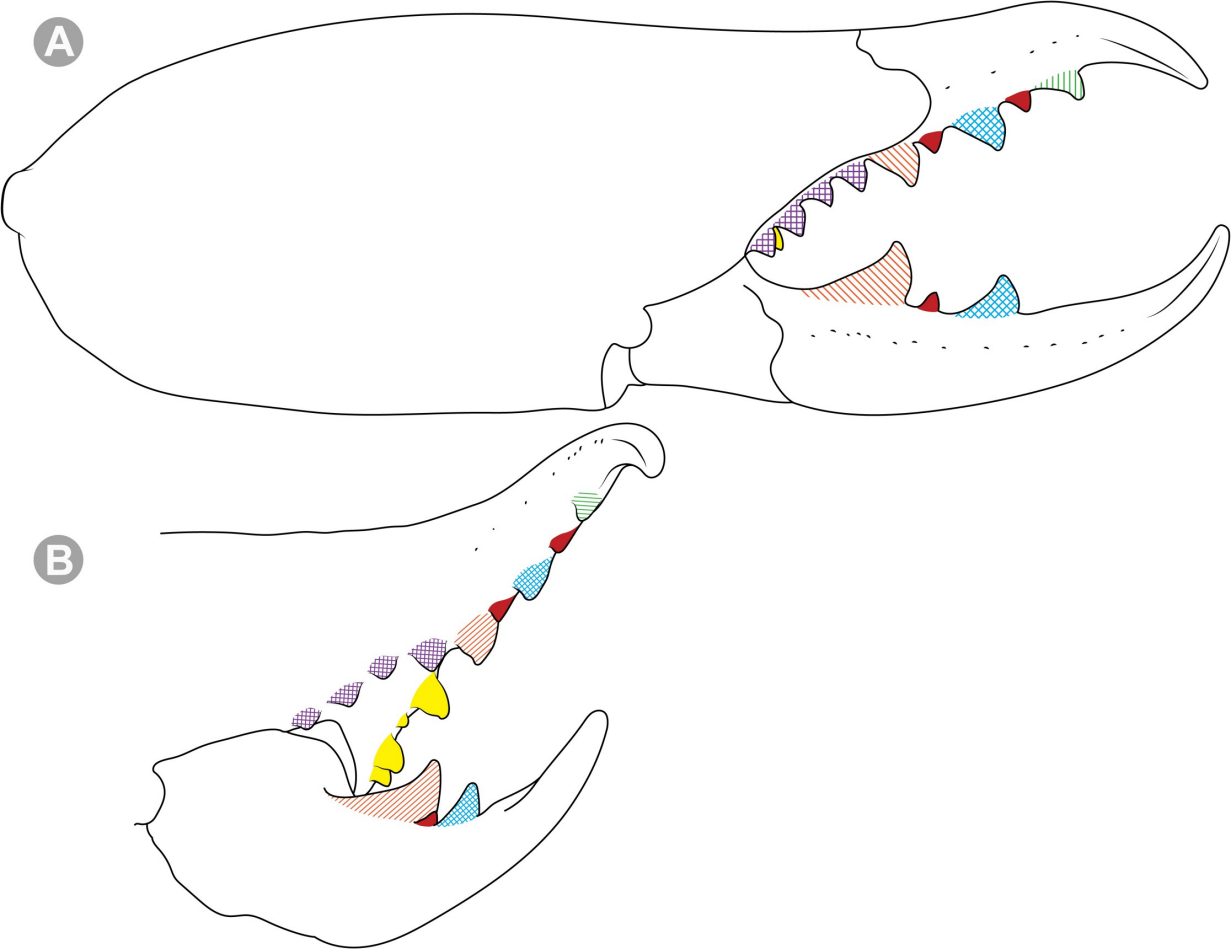
*Pseudocleobis chilensis* Roewer, 1934 holotype, schemes of right chelicera. (A) Retrolateral view. (B) Fixed finger, retroventral view.

***Pseudocleobis chilensis* Roewer, 1934 species *inquirenda*.** (Figs 9 and 10)

***Pseudocleobis chilensis*** Roewer, 1934 [7]: 605, figs 336i, 341e; Zilch, 1946 [27]: 152; Muma, 1971 [9]: 17, Figs 31–32; Cekalovic, 1975 [29]: 133; Maury, 1976 [10]: 94; Muma, 1976 [21]: 28; Harvey, 2003 [22]: 206.

**Type material (examined).** Holotype (adult female?); original label verbatim (Fig 9A): “*Arach. Coll. Roewer ‐ Lfd. No. 4761 / Solifuga No. 370 / Pseudocleobis chilensis / 1♀ n. sp. / Chile*: *Cuesta de Chacabuco / Typus Roewer det. 1933*”.

**Brief description.** The specimen seems to have been dried at some point (Fig 9C), and many setae were missing and fallen out; however, many of the spiniform setae are still present. The cuticula looks completely translucent and the yellowish coloration belongs to the contracted muscle bundles seen by transparency. All the limbs are present, except the left pedipalp that is broken and missing the tibia, basitarsus and tarsus (Fig 9C). *Legs*: The spiniform setal patterns as described for the genus. *Pedipalp*: Basitarsus with four pair of lateroventral spiniform setae, being the proximal pair setiform (especially the retroventral); tibia with five pairs of long lateroventral spiniform setae, the proximal more setiform, and the proventral longer that the retroventral; femur with a retroventral row of four long spiniform setae, and row of two proventral imperfectly aligned with the retroventral. *Chelicera* (Figs 9B and 10): Fixed finger median series with FD, 1FSD, FM, 1FSM and FP teeth (Figs 9B and 10A). Retrofondal series with four teeth, i.e. RFM, 1RFSM, RFP, 1RFSP (Figs 9B and 10A), the left chelicera has an additional very tiny RFSM tooth. Profondal series with four teeth, i.e., 1PFM, 1PFSM, 1PFP, 1PFSP (Fig 10B). Movable finger with MM, 1MSM and MP teeth. *Opisthosoma*: The cuticle is collapsed and shows unnatural folding (Fig 9C and 9D). The genital plate seems to show a wide middle opening (Fig 9D), but this seems to be an artifact of the dehydration, it is hard to assess if it is an adult female or a juvenile.

**Notes.** *Pseudocleobis chilensis* was described by Roewer [7] based on a single specimen from “Cuesta de Chacabuco”, Chile. This species has received very little attention, and the remaining bibliographic citations about it mostly consist of species lists and catalogs. There are no published redescriptions or any additional records of this species.

Roewer [7] identified this species by 4 fondal teeth on the ectal and medial series (nowadays 4RF and 4PF teeth), two intermediate teeth (nowadays 2FSM teeth), and the pedipalpal armature with 4 pairs of spiniform setae on femur and tibia, and 3 pairs on basitarsus. However, Roewer’s description has many errors, and now we know that most of the supposedly diagnostic characters that he used are common for most species of the genus. We consider that the described specimen does not present any morphological character that let us to distinguish it from other *Pseudocleobis*. The type locality “Cuesta de Chacabuco” refers to a relatively small geographic area with an altitudinal cline from 600 to 1350 m. a.s.l. on the Chacabuco mountain range, in Metropolitan Region, in central Chile. Given this, we consider this species as *species inquirenda*, and that an intensive sampling on the type locality should prove if it is possible to unequivocally assign new specimens to this species.

**Fig 11:**
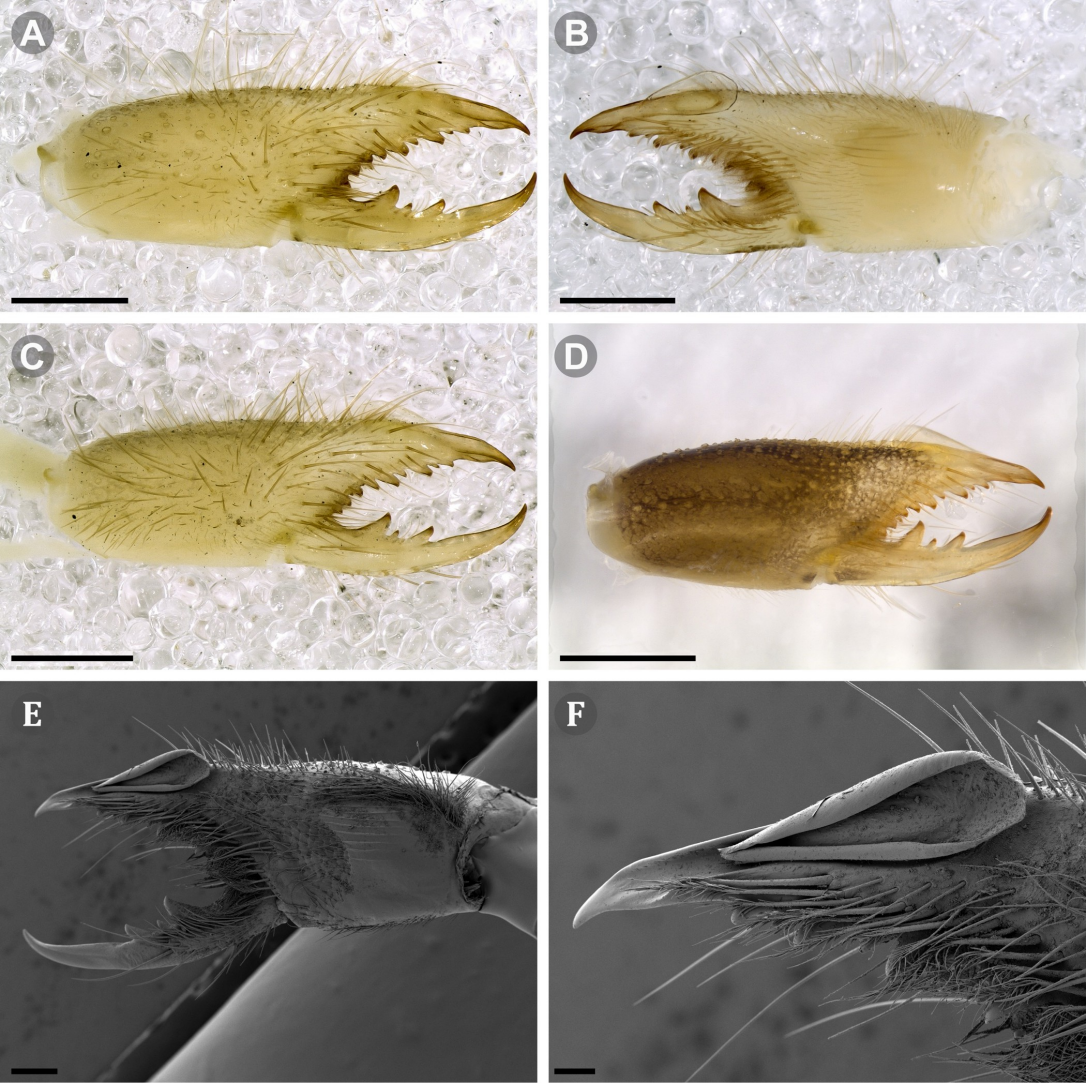
*Pseudocleobis alticola* Pocock, 1900, from Chile, male chelicera. (A, C, D) Retrolateral view, (B, E–F) prolateral view. (A–B) Male (MNNC; from Ollagüe, Antofagasta region). (C) Male (MNNC; from Chiu-Chiu, Antofagasta region). (D–F) Male (LEULS; from Salar de Lagunillas, Tarapacá region). Scale bars: 1 mm (A–D); 0,3 mm (E); 0,1 mm (F).

**Fig 12:**
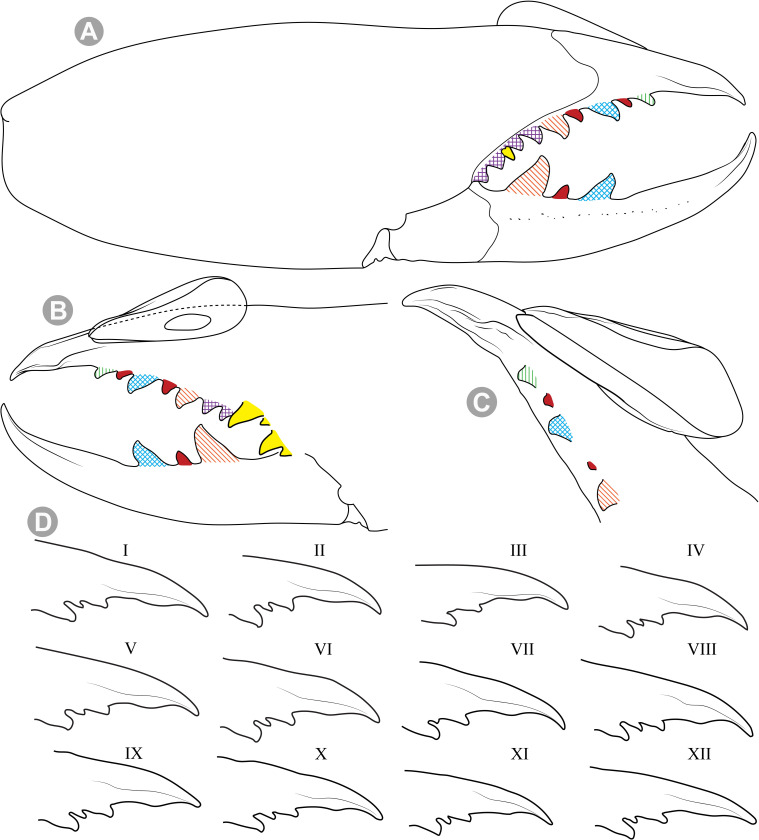
*Pseudocleobis alticola* Pocock, 1900, schemes of male chelicera. (A) Retrolateral view. (B) Retrolateral view. (C) Fixed finger, retroventral view. (D) Fixed finger mucron from six Chilean (I–VI) and six Argentinian (VII–XII) specimens. I–II (LEULS), III (MACN-Ar 46516), IV–V (MNNC), VI (MACN-Ar 46517), VII (MACN-Ar 7626), VIII (MACN-Ar 7632), IX (MACN-Ar 7628), X (MACN-Ar 7627), XI (MACN-Ar 7640), XII (MACN-Ar 7695).

***Pseudocleobis alticola* Pocock, 1900.** (Figs 5, 11 and 12).

***Pseudocleobis alticola*** Pocock, 1900 [19]: 304–305, Fig 8; Kraepelin, 1901 [6]: 109; Roewer, 1934 [7]: 605, figs 336h, 341d; Zilch, 1946 [27]: 152; Muma, 1976 [21]: 28; Maury, 1983 [12]: 170–172, Figs 3–4, 6; Maury, 1998 [4]: 568; Harvey, 2003 [22]: 206; Iuri & Iglesias, 2022 [2]: 439, 444.

*Pseudocleobis andinus (Pocock)*: Muma, 1971 [9]: 15, 16, Fig 24 misidentification (in part).

**Type material (not examined):** male holotype, Bolivia, La Paz department, Larecaja province, Mount Sorata, M. Conway coll., (BMNH).

**Previous records: BOLIVIA (not examined):** 1♀, “Humpnea and above”, M. Conway (BMNH). **ARGENTINA (examined): Jujuy:** 18♂, 5♀, Abra Pampa, 3500 m.a.s.l., 10-12-I-1966, E.A. Maury coll (MACN-Ar 7622); 3♂, 2♀, Purmamarca, 2143 m.a.s.l., 21-XI-1981, E.A. Maury & A. Roig coll (MACN-Ar 7623); 8♂, 1♀, Agua de Castilla, 10 km W of El Aguilar, 3600 m.a.s.l., 25-XI-1981, E.A. Maury & A. Roig coll (MACN-Ar 7624); 5♂, 3♀, Susques, 3675 m.a.s.l., 22-23-XI-1981, E.A. Maury & A. Roig coll (MACN-Ar 7625); 1♂, Sansana, 20-II-1952, R de la Fuente coll (MACN-Ar 7630); 1♂, 3♀, Humahuaca, II-1944, M. Birabén coll (MACN-Ar 7632); 2♂, La Quiaca, 6-XII-1951, R. de la Fuente coll (MACN-Ar 7631); 2♂, 1♀, same locality, II-1949, A. Martínez coll (MACN-Ar 7633); 5♂, 3♀, Mina Aguilar, 4700 m.a.s.l., 11-I-1960 (AMNH). **Salta:** 1♂, 1♀, San Antonio de los Cobres, 3775 m.a.s.l., 24-XI-1981, E.A. Maury & A. Roig coll (MACN-Ar 7628); 1♂, 3♀, Guachipas, 25-III-1947, M. Birabén coll (MACN-Ar 7627). **Salta/Jujuy limit:** 7♂, 1♀, Abra del Cóndor, road to Iruya, 4100 m.a.s.l., 26-XI-1981, E.A. Maury & A. Roig coll (MACN-Ar 7626); 1♂, 1♀, same locality, X-1972, E.A. Maury & R. Voyat coll (MACN-Ar 7629). **Catamarca:** 10♂, 1♀, Campo Arenal, 38 km NE of Hualfin, 14-I-1981, E.A. Maury & A. Roig coll (MACN-Ar 7695); 3♂, 2♀, Cuesta Minas, Capillitas, 8-II-1973, F. Enders coll (MACN-Ar 7639). **La Rioja:** 1♂, 2♀, Embalse Los Sauces, 7-8-X-1965, E.A. Maury coll (MACN-Ar 7640). **San Juan:** 1♂, Rodeo, 19-VIII-1982, J. Montivero coll (MACN-Ar 7740);

**New records**. **CHILE: Tarapacá:** 7♂, 2♀, 6 juveniles, Salar de Lagunillas, 4000 m.a.s.l., I-IV-2007, Ruiz, Gamboa and Ferrú coll., (LEULS); 1♂, Salar de Huasco, 3800 m.a.s.l., J.P. Castillo coll., (LEULS). **Antofagasta:** 1♂, Vegas de Loa, 10-VIII-1972, F. Soto coll., (MACN-Ar 46517); 1♂, Chiu Chiu, 25-X-1982, G. Arriagada coll., (MNNC); 1♂, 1♀, Ollagüe, 27-29-X-1982, G. Arriagada coll., (MNNC); 1♂, 1♀, 1 juvenil, Quebrada Turipite, 20-VIII-1962, F. di Castri coll., (SMF 17370–3; examined by photos); 1♂, San Pedro de Atacama, 14-I-1963, D. Ibarra Grasso coll., (MACN-Ar 46516); 2♂, El Loa, Laguna Lejía, Antojunta, XI-1968, (AMNH; not examined, chelicera illustrated in Muma [9] Fig 24); 1♂, Pajonal, 21-I-1997, (LEULS).

**Diagnosis.** *Pseudocleobis alticola* can be diagnosed by the morphology of the male fixed finger mucron, with a straight flagellar groove slightly pointed downwards (Figs 6C, 11B, 11E, 11F and 12B), with long and straight prodorsal flange (Figs 6C, 11E, 11F and 12B), with the proventral flange curved upwards (Figs 6C, 11E, 11F and 12B) and without retroventral flange (Figs 11A, 11C, 11D, 12A and 12C).

**Notes.** Slight variations were observed, mostly in the mucron morphology, between specimens from Argentina, Bolivia and Chile (Fig 12C); however, we didn’t find a clear pattern to separate them as different species. The wide distribution (ranging from the high Andes of Argentina, Bolivia and Chile) suggests that our current concept of *P. alticola* might actually correspond to a species group of high-altitude species, which diversified relatively recently, after the last Andean uplift. However, given the conserved morphology, we prefer to maintain it as a single species until we can study this group in depth.

**Distribution and ecology:** This species of *Pseudocleobis* is the only one that we have recorded in different habitats of the Chilean high Andean plateau or “Altiplano” of the Atacama Desert. This is considered the highest plateau of the Andes Mountains chain, an area that presents a marked climatic variation not only during the year, but also during the day. This area offers a unique ecosystem with extreme conditions, such as daily temperature changes from -10 to +25°C. The area where this species has been collected corresponds to the high-Andean altiplanic steppe and pre-Puneña shrubby steppe botanical regions [17].

**Fig 13:**
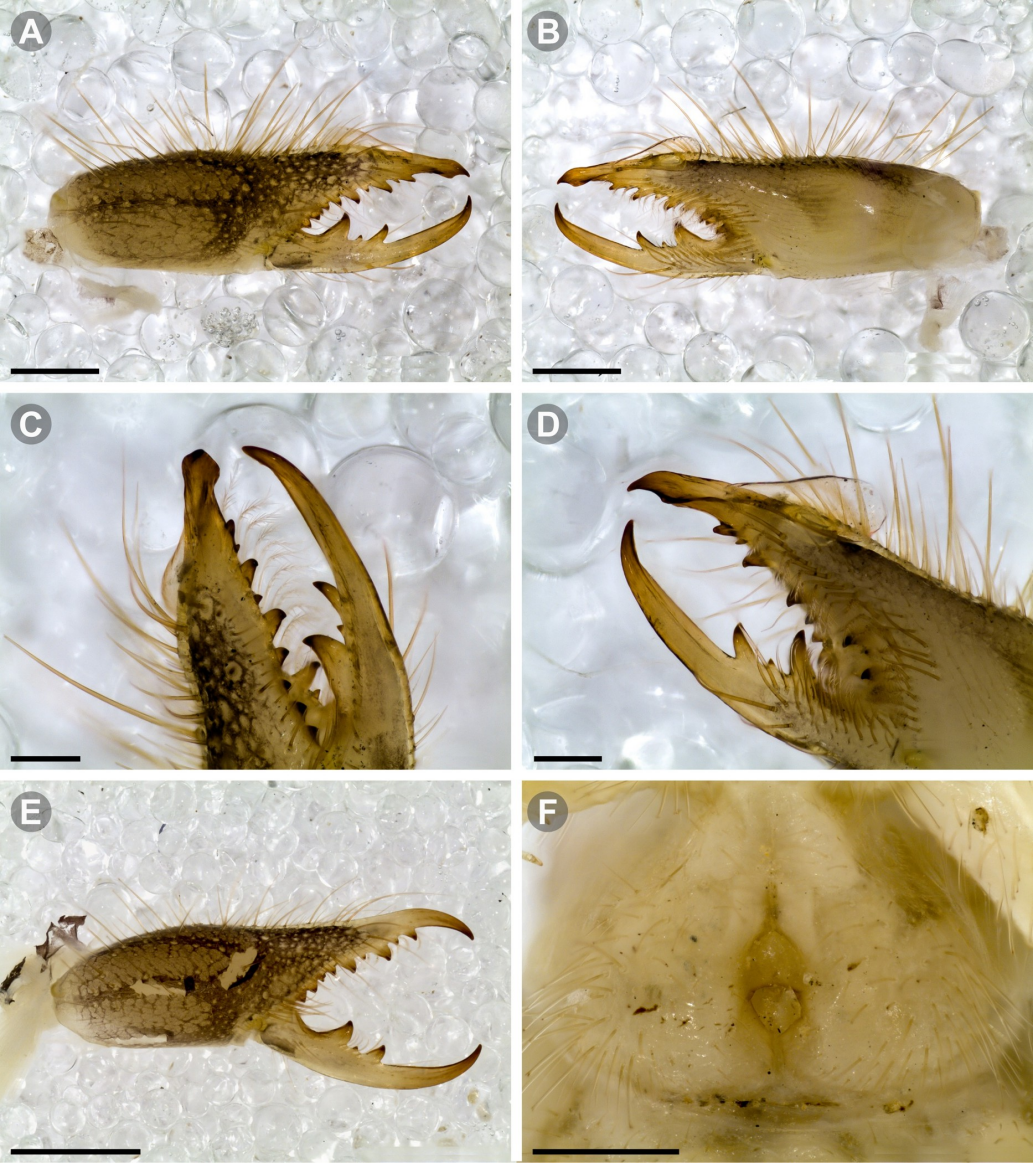
*Pseudocleobis elongatus* n. sp. (A–D) Male holotype, right chelicera. (E–F) Female paratype. (A) Retrolateral view. (B) Prolateral view. (C) Cheliceral fingers, retroventral view. (D) Cheliceral fingers, proventral view. (E) Right chelicera, retrolateral view. (F) Genital sternite, ventral view. Scale bars: 1 mm (E); 0,5 mm (A–B, F); 0,2 mm (C–D).

**Fig 14:**
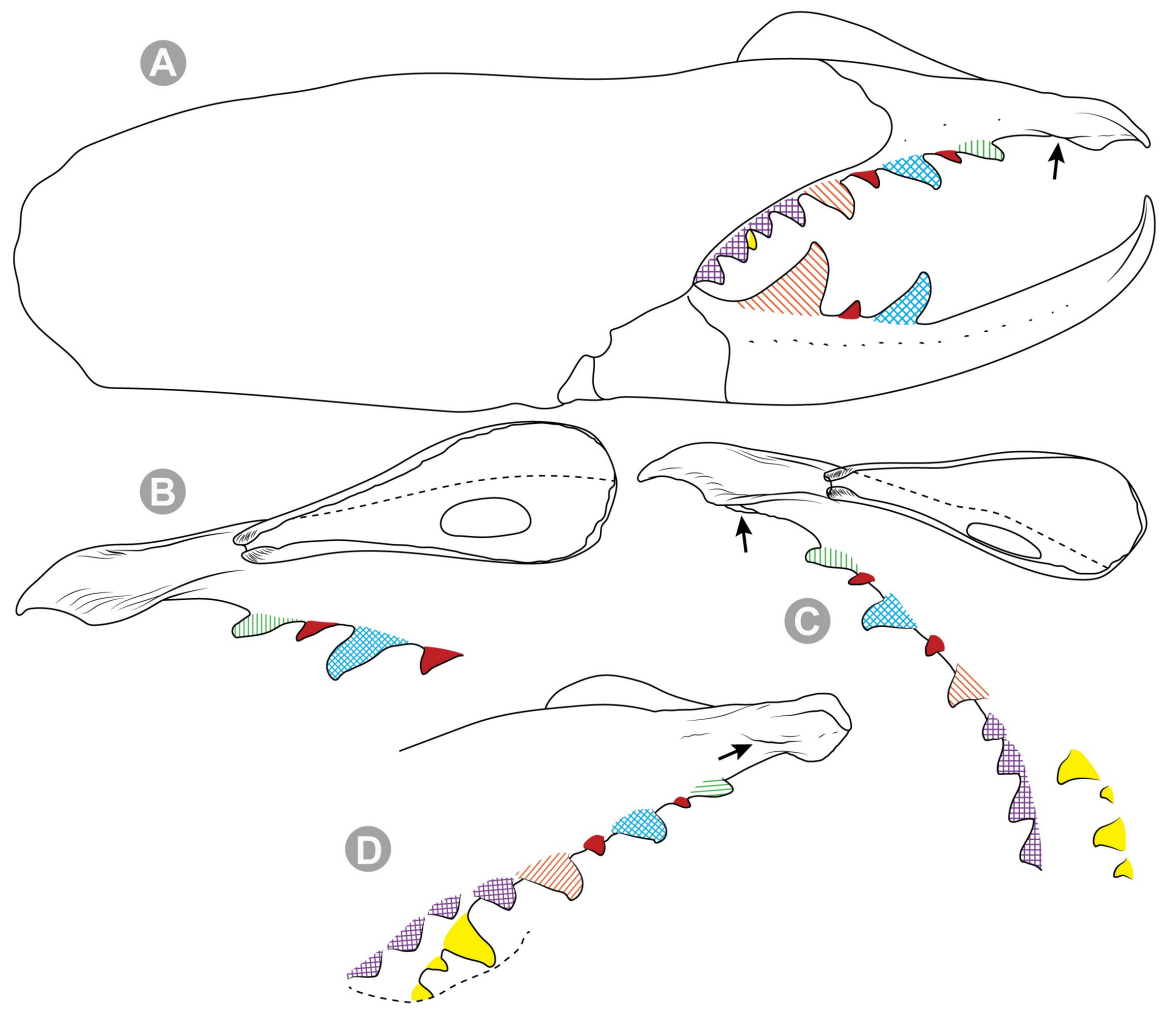
*Pseudocleobis elongatus* n. sp., schemes of male right chelicera. (A) Retrolateral view. (B) Fixed finger, prolateral view. (C) Fixed finger, proventral view. (D) Retroventral view. Arrow indicates the small retrolateral flange.

**Fig 15:**
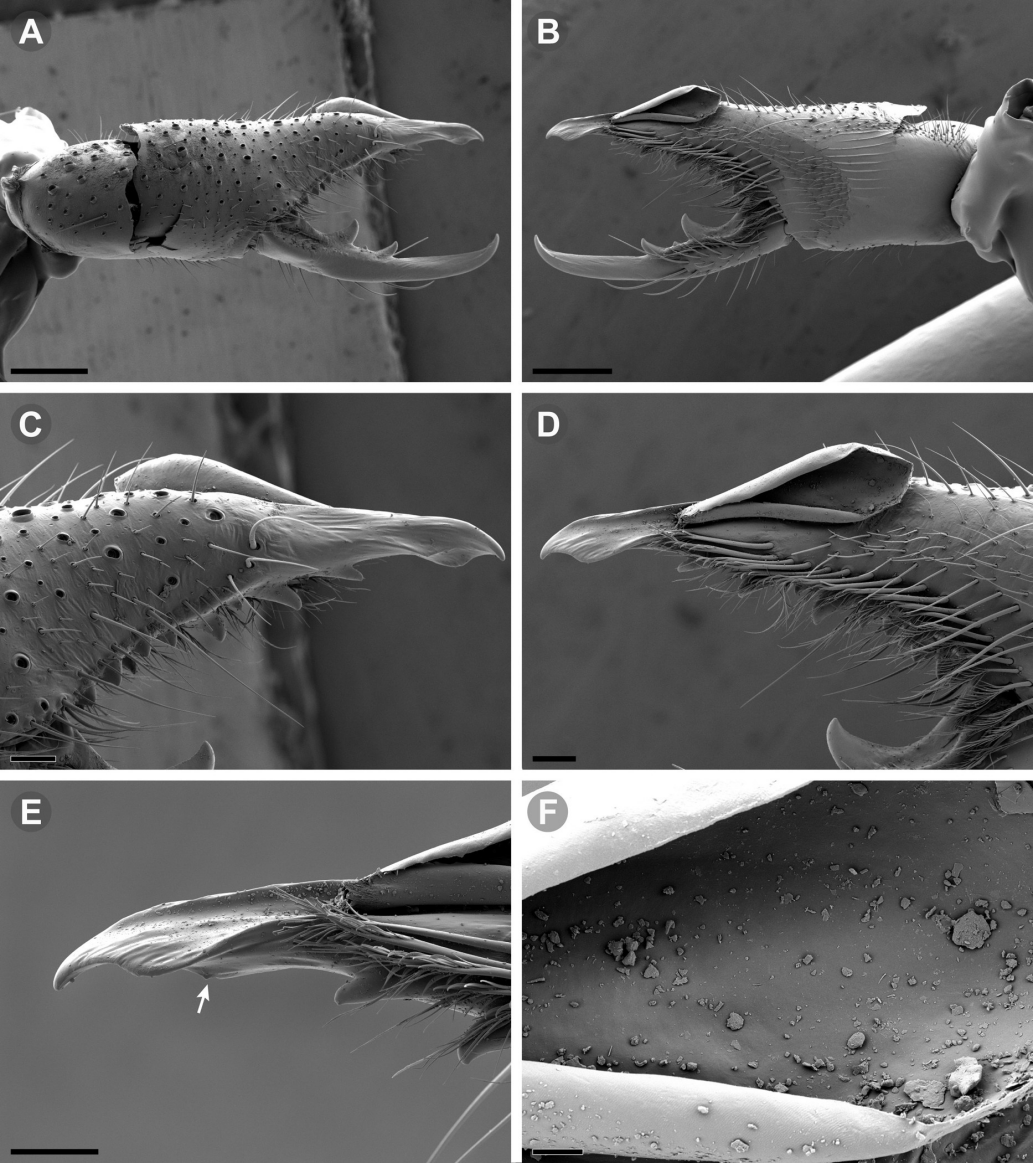
*Pseudocleobis elongatus* n. sp., SEM images of male right chelicera. (A) Retrolateral view. (B) Prolateral view. (C) Fixed fingers, retrolateral view. (D) Fixed fingers, prolateral view. (E) Fixed finger mucron, proventral view, arrow indicates the retroventral flange. (D) Flagellum, smooth prolateral surface detail. Scale bars: 0,4 mm (A–B); 0,1 mm (C–E); 0,02 mm (F).

***Pseudocleobis elongatus* Iuri n. sp.** urn:lsid:zoobank.org:act:C8A3C785-577F-4AF7-AC2F-417BA3CA6CA8.

(Figs 2B, 5 and 13–15 and Tables 1 and 2).

**Table 1:** Metric data for *Pseudocleobis elongatus* n. sp., *P. atacamensis* n. sp. and *P. puna* n. sp. Measurements in millimeters for male holotype and one female (when known).

		*P. elongatus* n. sp.		*P. atacamensis* n. sp.	*P. puna* n. sp.
		♂ (holotype)	♀	♂ (holotype)	♂ (holotype)
Total body	L^1^	6,17	8,59	10,6	14,02
Propeltidium	L	1,45	1,38	1,9	2,18
	W	1,74	2,04	2,42	2,77
Chelicera	L^2^	2,45	3,29	3,6	3,92
	W^3^	0,71	0,91	0,98	1,19
	H^4^	0,73	0,95	1,08	1,35
Pedipalp	L^5^	14,22	10,19	19,15	23,38
	femur L	5,17	3,63	6,24	8,39
	tibia L	5,27	3,73	7,57	8,8
	basitarsus L	3	2,12	4,18	4,81
	telotarsus L	0,78	0,71	1,16	1,38
Leg I	L^5^	10,28	7,77	16,1	17,74
	patela L	4,11	2,7	6,1	6,78
	tibia L	3,54	2,84	5,71	6
	basitarsus L	1,78	1,43	3,06	3,43
	telotarsus L	0,85	0,8	1,23	1,53
Leg IV	L^5^	15,84	12,7	22,84	23,92
	patela L	5,47	4,3	7,36	7,47
	tibia L	5,05	4,15	7,43	7,75
	basitarsus L	3,72	2,97	5,26	5,62
	telotarsus L^6^	1,6	1,28	2,79	3,08

L = length; W = width; H = height.

^1^ Measured along medial axis, from the propeltidium anterior margin to the opisthosoma posterior margin.

^2^ Measured in retrolateral view from chelicero-peltidial condyle to the tip of fixed finger mucron.

^3^ Measured in dorsal view at widest point.

^4^ Measured in retrolateral view at highest point of the manus.

^5^ Sum of individual segments length.

^6^ Measurement excludes claws.

**Table 2:** Variability on metric data for *Pseudocleobis elongatus* n. sp., *P. atacamensis* n. sp. and *P. puna* n. sp. Values in millimeters.

		*P. elongatus* n. sp.						*P. atacamensis* n. sp.			*P. puna* n. sp.		
		male (n = 4)			female (n = 4)			male (n = 5)			male (n = 2)		
		MIN	MEAN	MAX	MIN	MEAN	MAX	MIN	MEAN	MAX	MIN	MEAN	MAX
Total body	L^1^	5,93	6,20	6,49	6,79	8,33	10,43	7,47	9,25	10,60	10,94	12,48	14,02
Propeltidium	L	1,36	1,41	1,46	1,38	1,50	1,78	1,70	1,92	2,11	2,09	2,14	2,18
	W	1,50	1,69	1,77	2,04	2,41	2,66	2,12	2,44	2,79	2,69	2,73	2,77
Chelicera	L^2^	2,39	2,43	2,47	3,29	3,79	4,61	3,14	3,47	3,97	3,80	3,86	3,92
	W^3^	0,69	0,70	0,72	0,91	1,17	1,48	0,90	1,00	1,14	1,19	1,21	1,24

L = length; W = width; H = height.

^1^ Measured along medial axis, from the propeltidium anterior margin to the opisthosoma posterior margin.

^2^ Measured in retrolateral view from chelicero-peltidial condyle to the tip of fixed finger mucron.

^3^ Measured in dorsal view at widest point.

**Type material.** Holotype: CHILE: Antofagasta region: ♂, Antofagasta province, La Chimba National Reserve, 23°31’24.2’’S 70°20’26.8’’W, 712 m. a.s.l., 19-20-XI-2022, H.A. Iuri, A.A. Ojanguren-Affilastro, J. Pizarro-Araya, F.M. Alfaro, J.E. Calderón & J.E. Barriga-Tuñón coll., (MNNC 8418). Paratype: CHILE: Antofagasta region: 1♀, same data as holotype (MNNC 8419); 1♂, 1♀, same data as holotype (LEULS 186–187); 1♂, 2♀, same data as holotype (MACN-Ar 46518–46520); 1♂, Antofagasta province, Caleta El Cobre, 24°16’14.0’’S 70°31’02.6’’W, 324 m. a.s.l., 17-18-XI-2022, H.A. Iuri, A.A. Ojanguren-Affilastro, J. Pizarro-Araya, F.M. Alfaro, J.E. Calderón & J.E. Barriga-Tuñón coll., (MACN-Ar 46521).

**Etymology.** The specific epithet *elongatus* means ’prolonged’ or ’elongated’ and is the perfect passive participle of the Latin verb *elongo*, and refers to the long-legged appearance of the male.

**Diagnosis.** Differs from *Pseudocleobis krausi* n. sp., *P. choros* n. sp., *P. lalackama* n. sp., *P. mumai* n. sp., *P. cekalovici* n. sp. and *P. escuadra* n. sp. by the long, straight prodorsal flange, and differs from *P. alticola* by the proventral flange not curved upwards (Figs 13B, 14B, 15B and 15D). This species resembles *P. atacamensis* n. sp. and *P. puna* n. sp. in the male fixed finger mucron morphology (Figs 13B, 14B, 15B and 15D), and the flagellum pear-shaped (Figs 13B, 13D, 14B, 14C, 15B, 15D). It differs from both by the proventral flange of the flagellar groove, that turns down distally forming a wide cuticular extension near the tip (Figs 13A, 13B, 13D, 14A–14C and 15A–15E), and by the presence of a small retroventral flange on fixed finger mucron (Figs 13C, 13D, 14C, 14D, 15C and 15E).

**Description.** male holotype: *Chelicerae*: Dentition and processes: fixed finger: median series with FD, 1 FSD, FM, 1 FSM and FP teeth (Fig 14A), FP and FM are similar sized and are the biggest teeth on fixed finger; retrofondal series with RFM, 1 RFSM, RFP, 1 RFSP teeth and profundal series with PFM, 1 PFSM, PFP, 1 PFSP teeth (Fig 14C). Fixed finger mucron relatively long, and ending in a curved pointed tip; flagellar groove almost straight, directed downwards (Figs 14B and 15D), with a long prodorsal flange that ends almost at the tip (Fig 15D) and a long proventral flange that curves downwards at distal forming a wide distal cuticular expansion (Figs 14B, 14C, 15D and 15E); there is a small retroventral flange at the middle of the mucron (Figs 14C, 14D, 15C and 15E) Movable finger: with MM, 1 MSM and MP teeth (Fig 14A); MM bigger than FM and FP; MP is the biggest tooth of the chelicera. Movable finger mucron approx. 1.5 times as long as fixed finger mucron, slightly curved; gnathal edge carina poorly elevated and not forming a convex bulge; mucron tip distinctly curved upwards (Fig 15A and 15B). Stridulatory plate with 8 parallel ridges. *Flagellum*: flagellum base pear-shaped (Fig 14B), narrowing distally, dorsal margin runs slightly concave to distal, ventral margin is markedly concave distally (Figs 14B and 15D). The stalk (attachment ring) situated on the distal end of the prolateral setose area, longitudinally between FSM and FP teeth. Prolateral surface smooth (Fig 15F). *Pedipalps*: Femur with a proventral row of five long lateroventral spiniform setae, and a imperfectly aligned retroventral row of seven lateroventral spiniform setae; the retroventral smaller than the proventral and imperfectly paired with these. Tibia with 2.2.2.2.2 long ventral spiniform setae, the proximal pair setiform, the retroventral row smaller and more similar in size, the proventral row longer and arranged in increasing size I = V, II = IV, III; there is one pair of apical setae that seems like a weak pair of spiniform setae, but the insertion socket is different being completely circular and not strongly elevated. Basitarsus with 2.2.2.2 long ventral spiniform setae, the proximal pair setiform, the retroventral smaller than the proventral, but more equally sized in each row than those of the tibia. *Opisthosoma*: The ctenidia are present and hard to distinguish, 2–2 on sternite III and 3–3 on sternite IV.

**Female.** Morphology and chaetotaxy like males, except by the wider propeltidium, chelicerae without modified mucron (Fig 13E), and shorter legs and pedipalps. The spiniform setae of pedipalps are more spiniform than males. Opisthosomal sternite III and IV without ctenidia. Genital plate (Fig 13F) bell-shaped, with lateral margins slightly concave; with a long middle cuticular aperture, and with another circular aperture inside; the two halves touch each other posteriorly.

**Variability.** One female paratype has and additional small retroventral spiniform seta on basitarsus of left pedipalp, between the subdistal and subbasal ones (Fig 8F). Measurements and size variability in Tables 1 and 2.

**Distribution and ecology.** This species has been collected in La Chimba National Reserve, an area belonging to the Chilean national system of protected areas (SNASPE, CONAF). This reserve is located 15 kilometers northeast of Antofagasta and is situated in the Chilean Coastal Range. *Pseudocleobis elongatus* n. sp. has been collected in the internal area of the reserve, in areas of the coastal range, and in the external eastern part of the reserve, in open plain spaces. The area corresponds to the Tocopilla Coastal Desert botanical region [17]. La Chimba hydrographic basin, is composed of two main drainages, La Chimba Ravine and Guanaco Ravine, where small water pools and forms of erosion of volcanic relief are notable [35]. These ravines are important biological reservoirs in the coastal desert of northern Chile [36]. In the same area where this species has been collected, we have also found many other species of endemic arachnids, including several endemic scorpion species [37–39], and another solifuge species of the genus *Chileotrecha*. The particular conditions of this area, including the isolated ravines protected by La Chimba Reserve, proved to be an important biological relict of the coastal desert of northern Chile.

This species has also been collected in El Cobre Cove, 60 km south of Antofagasta city, in a similar environment to La Chimba Reserve. The vegetation in the area consists of an extremely xeromorphic open coastal desert scrub dominated by *Eulychnia iquiquensis* (K. Schum.) Britton & Rose 1920, *Atriplex clivicola* IM Johnst 1929, *Frankenia chilensis* C. Presl ex Schult. & Schult.f. 1830, *Nolana lachimbensis* Dillon, Arancio & Luebert 2007, *Ophryosporus triangularis* Meyen 1834, and *Tetragonia angustifolia* Barnéoud 1847 [35].

**Fig 16:**
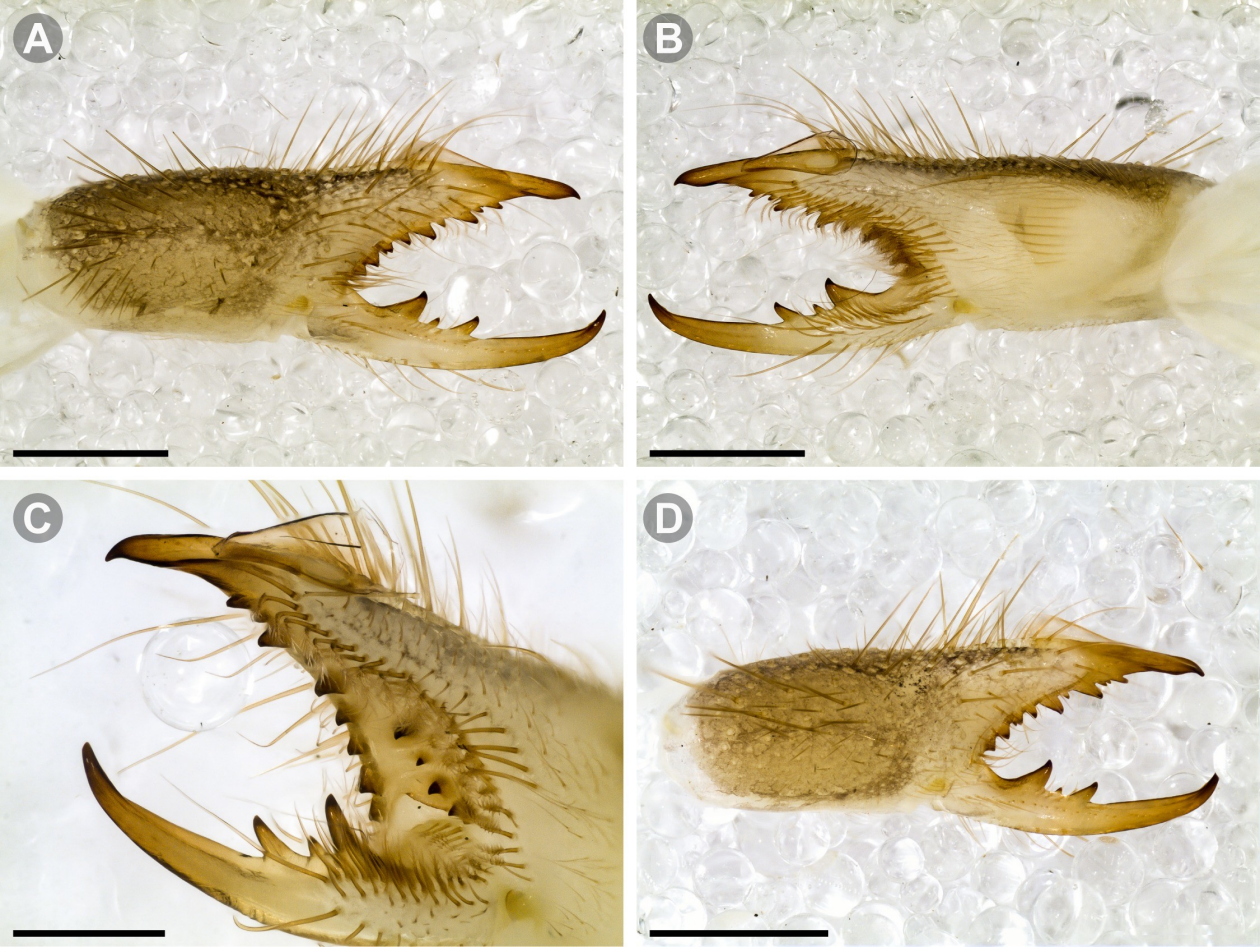
*Pseudocleobis atacamensis* n. sp., male holotype, right chelicera. (A) Retrolateral view. (B) Prolateral view. (C) Proventral view. (D) Male paratype, right chelicera, retrolateral view, note the two FSD teeth. Scale bars: 1 mm (A–B, D); 0,5 mm (C).

**Fig 17:**
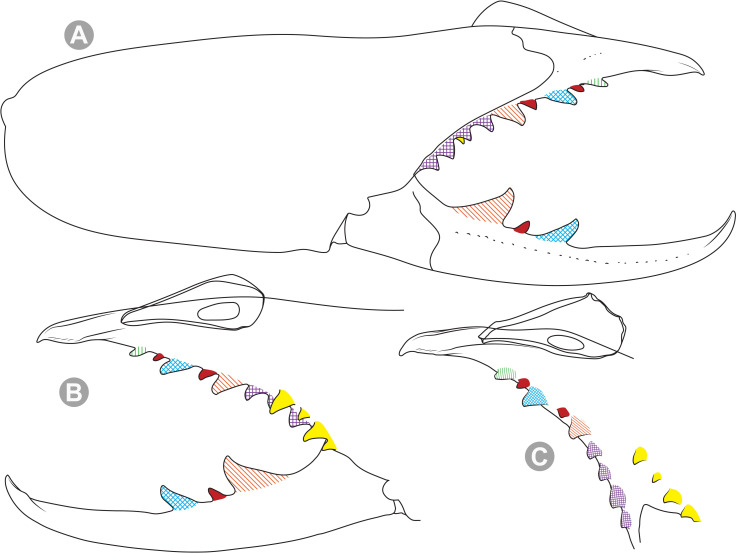
*Pseudocleobis atacamensis* n. sp., schemes of male right chelicera. (A) Retrolateral view. (B) Cheliceral fingers, prolateral view. (C) Fixed finger, proventral view.

**Fig 18:**
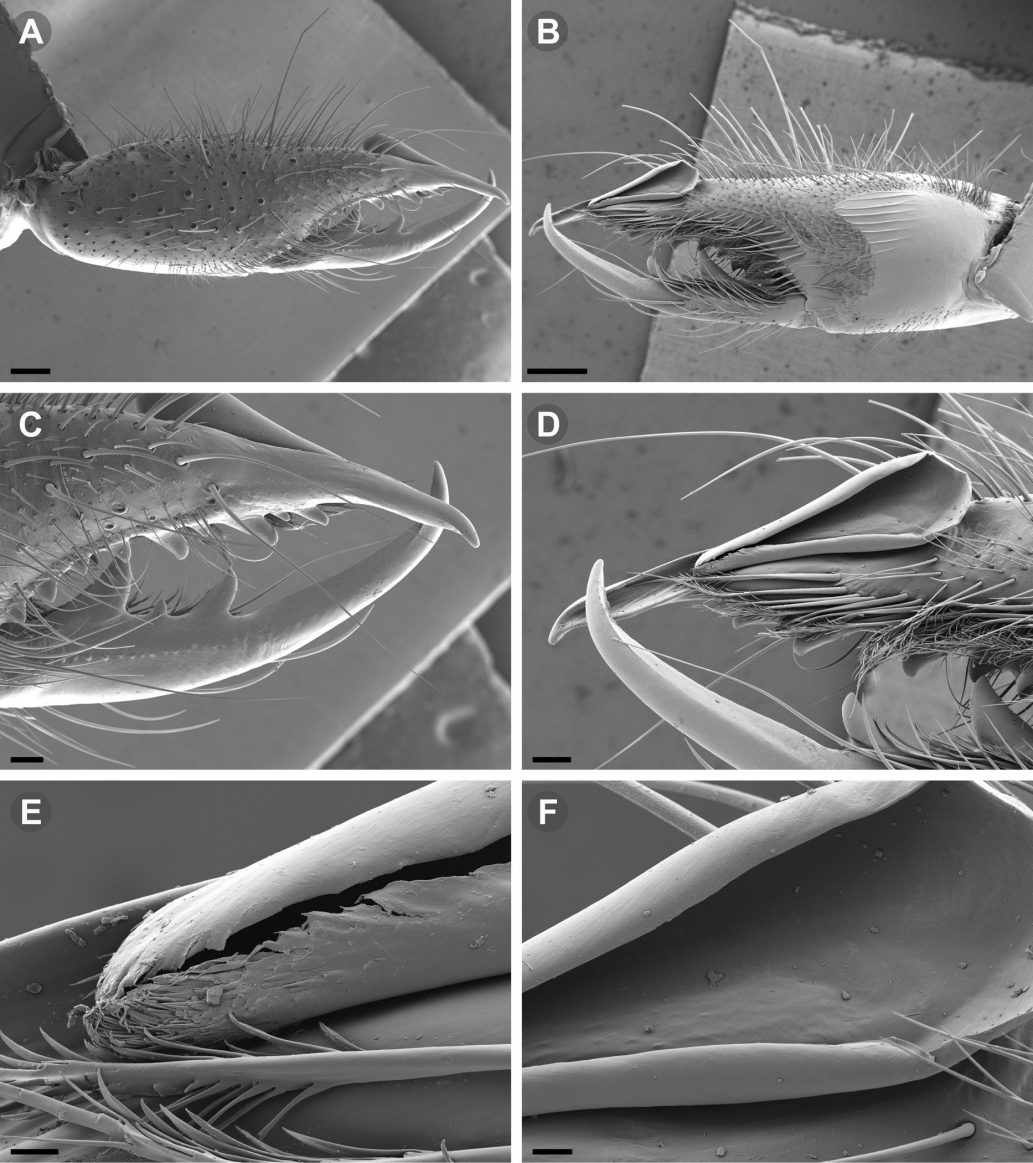
*Pseudocleobis atacamensis* n. sp., SEM images of male right chelicera. (A) Retrolateral view. (B) Prolateral view. (C) Cheliceral fingers, retrolateral view. (D) Cheliceral fingers, prolateral view. (E) Flagellum apex detail. (D) Flagellum smooth prolateral surface detail. Scale bars: 0,4 mm (A), 0,1 mm (B), 003 mm (D), 0,02 mm (C).

***Pseudocleobis atacamensis* Iuri & Maury n. sp.** urn:lsid:zoobank.org:act:1755B339-83FD-41D9-985E-C8F8502EAC0B.

(Figs 2D, 5 and 16–18 and Tables 1 and 2).

*Pseudocledobis andinus* (Pocock): Kraus, 1966 [8]: 182 (misidentification in part).

**Type material.** Holotype: CHILE: Atacama region: ♂, Copiapó province, Inca de Oro, La Vega, 26°40’45.9’’S 69°39’28.0’’W, 2,271 m. a.s.l., 23-XI-2022, H.A. Iuri, A.A. Ojanguren-Affilastro, J. Pizarro-Araya, F.M. Alfaro, J.E. Calderón & J.E. Barriga-Tuñón coll (MNNC 8420). Paratype: CHILE: Atacama region: ♂, Copiapó province, Inca de Oro, camp, 26°40’32.9’’S 69°50’21.5’‘W, 1,767 m. a.s.l., 22-23-XI-2022, same collectors as holotype (LEULS 188); ♂, same data as the anterior (MZUC 48062); ♂, Copiapó province, Boquerón Chañar, El Donkey, 28°22’15.6’’S 70°24’46.2’’W, 992 m. a.s.l., 24-25-XI-2022, same collectors as holotype (MACN-Ar 46522); ♂, Copiapó province, Torres de Labrar, 28°40’27.2’’S 71°13’53.0’’W, 532m. a.s.l., 25-XI-2022, same collectors as holotype (MACN-Ar 46523).

**Other material.** CHILE: Atacama region: ♂, Copiapó province, Dulcinea, 26-VIII-1963, F. di Castri (SMF 17368; examined by Maury, unpublished drawings and description at MACN; S1 Fig).

**Etymology.** Latinized demonym from the Atacama region, where this species is found.

**Diagnosis:** This species differs from *Pseudocleobis krausi* n. sp., *P. choros* n. sp., *P. lalackama* n. sp., *P. mumai* n. sp., *P. cekalovici* n. sp. and *P. escuadra* n. sp. by the long, straight prodorsal flange, and differs from *P. alticola* by the proventral flange not curved upwards (Figs 16B, 17B and 18D). This species resembles *P. elongatus* n. sp. and *P. puna* n. sp. in the male fixed finger mucron morphology (Figs 17B and 18D), and the flagellum pear-shaped (Figs 16B, 17B, 18B and 18D). It differs from both by the proventral flange of the flagellar groove, that curves at distal third of the mucron (Figs 17B, 18C and 18D) (no near the tip as *P. puna* n. sp.) and doesn’t turn down forming a wide cuticular extension (contrary to *P. elongatus* n. sp.), and fixed finger mucron doesn’t have retroventral flange (Figs 16C and 17C).

**Description.** Male holotype: *Chelicerae*: Dentition and processes: fixed finger: median series with FD, 1 FSD, FM, 1 FSM and FP teeth (Fig 17), FP and FM similarly sized being the biggest teeth on fixed finger; retrofondal series with RFM, 1 RFSM, RFP, 1 RFSP teeth (Fig 17) and profondal series with PFM, 1 PFSM, PFP, 1 PFSP teeth (Fig 17). Fixed finger mucron relatively long and ending in a curved pointed tip (Figs 17 and 18A); flagellar groove directed downwards (Figs 17B and 18D), with a long prodorsal flange that ends almost at the tip (Fig 18D) and a long proventral flange that curves at distal, forming in lateral view a convex ventral bulge in the distal half of the mucron (Fig 17A and 17B). Movable finger: with MM, 1 MSM and MP teeth (Fig 17A and 17B); MM is bigger than FM and FP; MP is the biggest tooth of the chelicera. Movable finger mucron is approx. 1.5 times as long as fixed finger mucron, slightly curved, and the gnathal edge carina is poorly elevated and doesn’t form a convex bulge (Fig 16A and 16D); the tip is distinctly curved upward (Figs 16A and 18D). Stridulatory plate with 8 parallel ridges (Figs 16B and 18B). *Flagellum*: flagellum base pear-shaped, narrowing distally (Figs 16B, 17B, 18B and 18D). The dorsal margin of the base runs slightly concave to distal, while the ventral margin is markedly concave to distal. The stalk (attachment ring) is situated at a level between FM and FP teeth (Fig 16B). Prolateral surface smooth (Fig 18F). *Pedipalps*: Femur with a proventral row of five long lateroventral spiniform setae, and a imperfectly aligned retroventral row of seven lateroventral spiniform setae; the retroventral being smaller than the proventral and imperfectly paired with these. Tibia with 2.2.2.2.2 long ventral spiniform setae, the proximal pair setiform, the retroventral row smaller and more similar in size. Basitarsus with 2.2.2.2 long ventral spiniform setae, the proximal pair setiform, the retroventral smaller than the proventral, but more equally sized in each row than those of the tibia. *Opisthosoma*: The ctenidia are present and hard to distinguish, 0–2 on spiracular sternite I and 0–2 on spiracular sternite II (but see variability section).

**Female.** unknown.

**Variability.** The number of retroventral spiniform setae on the femur of pedipalp varies in the paratypes being 5, 6 or 7 spiniform setae. The male paratype (LEULS 188) has one additional, small, distal FSD tooth on the right chelicera (Fig 16D). The male paratype (MZUC 48062) has one tiny additional proximal RFSM on both chelicerae. The male paratype (MACN-Ar 46523) has one tiny additional distal RFSM on the right chelicera, and has 3–3 and 3–4 ctenidia on sternites III and IV respectively. The male paratype (MACN-Ar 46522) has 3–3 and 3–4 ctenidia on sternites III and IV respectively. Size variability in Table 2.

**Distribution and ecology.** This species is known from several mid-altitude localities in the Atacama region, and was collected in areas of the inland Mediterranean desert scrub of *Adesmia argentea* Meyen and *Bulnesia chilensis* Gay [40]. These areas correspond to very open scrublands with tall shrubs and isolated tree species; among the shrubs are *A. argentea*, *Bulnesia chilensis*, *Balsamocarpon brevifolium* Clos, *Cordia decandra* Hook. & Arn., *Heliotropium sinuatum* (Miers) I.M. Johnst., and *Proustia ilicifolia* Hook. and Arn. Low shrubs such as *Caesalpinia angulate* (Hook. & Arn.) Baill., *Encelia canescens* Lam., and the cacti *Cumulopuntia sphaerica* (C.F. Först.) E.F. Anderson and *Trichocereus coquimbanus* (Molina) Britton & Rosa. Herbaceous plants are abundant during the rainy season such as *Cruckshanksia pumila* Clos and *Argylia radiate* (L.) D. Don. Among the trees, it is characterized by the presence of *Neltuma chilensis* (Molina) Hughes & Lewis, *Geoffroea decorticans* (Gillies ex Hook. & Arn.) Burkart, *Acacia caven* (Molina) Molina and *Schinus polygama* (Cav.) Cabrera [40]. The area where this species has been collected corresponds to the steppe desert of the hills of El Salvador, flowery desert of the interior plains and the coastal desert of Huasco botanical regions [17].

The collections carried out in this area are related to the “flowery desert”, a phenomenon of the desert after important rains (i.e., ENSO [El Niño-Southern Oscillation] years or humid years), an instance that originates a biological peak, and where we have been able to record this species and other new arthropod taxa.

All specimens were collected at night and were found running on the ground. In Boquerón Chañar, this species was found in sympatry with *Ammotrechelis* sp., while other species like *P. krausi* n. sp., two species of *Chileotrecha*, and at least, three species of Mummuciidae were found in close areas to the collection site. In Inca de Oro, at least three species of Mummuciidae were found in sympatry with this species, but all these are active during the day.

***Pseudocleobis puna* Iuri n. sp.** urn:lsid:zoobank.org:act:E071CDF7-5A42-43D2-895E-AE36AD30EDD4.

**Fig 19:**
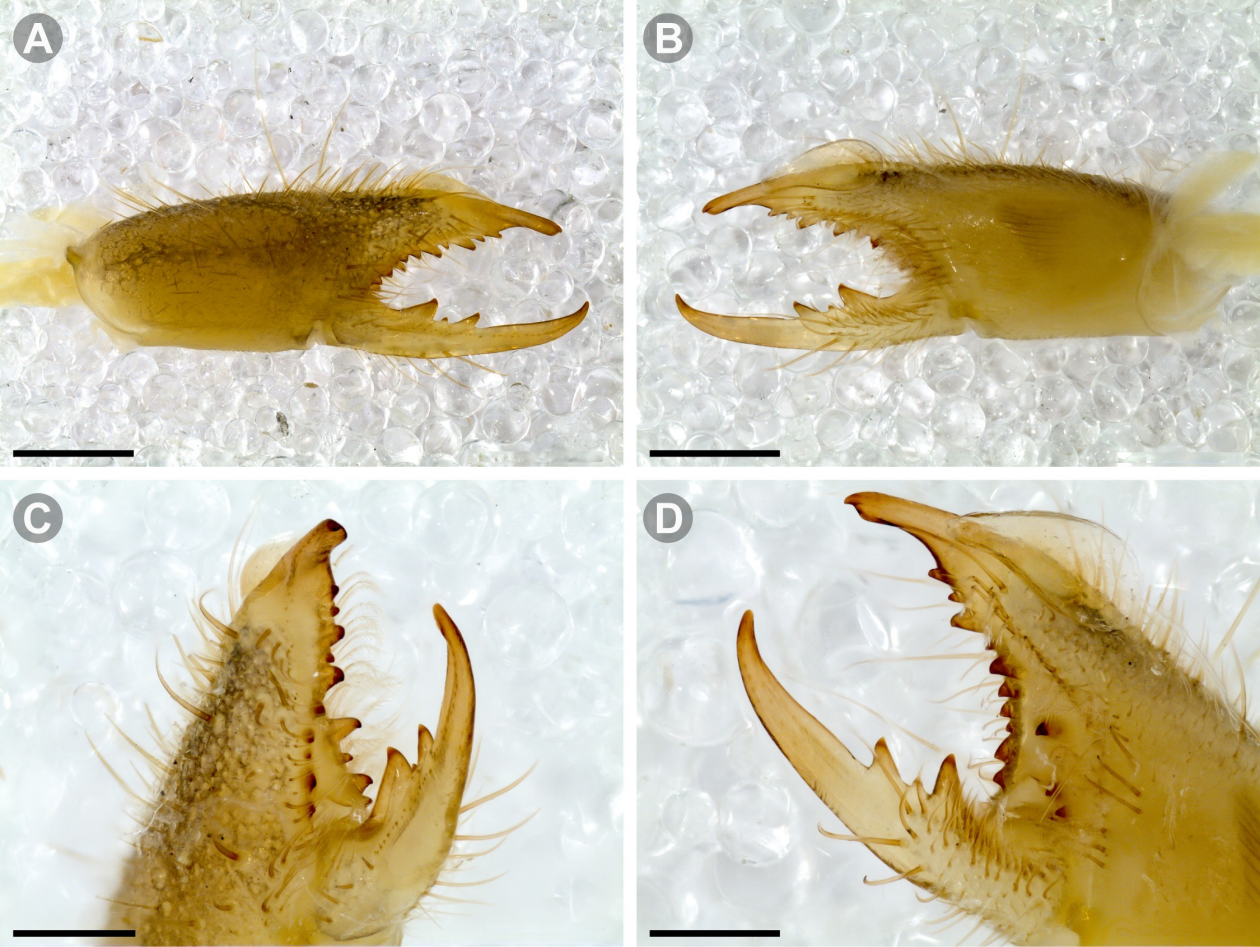
*Pseudocleobis puna* n. sp., male holotype, right chelicera. (A) Retrolateral view. (B) Prolateral view. (C) Cheliceral fingers, retroventral view. (D) Cheliceral fingers, proventral view. Scale bars: 1 mm (A–B); 0,5 mm (C, D).

**Fig 20:**
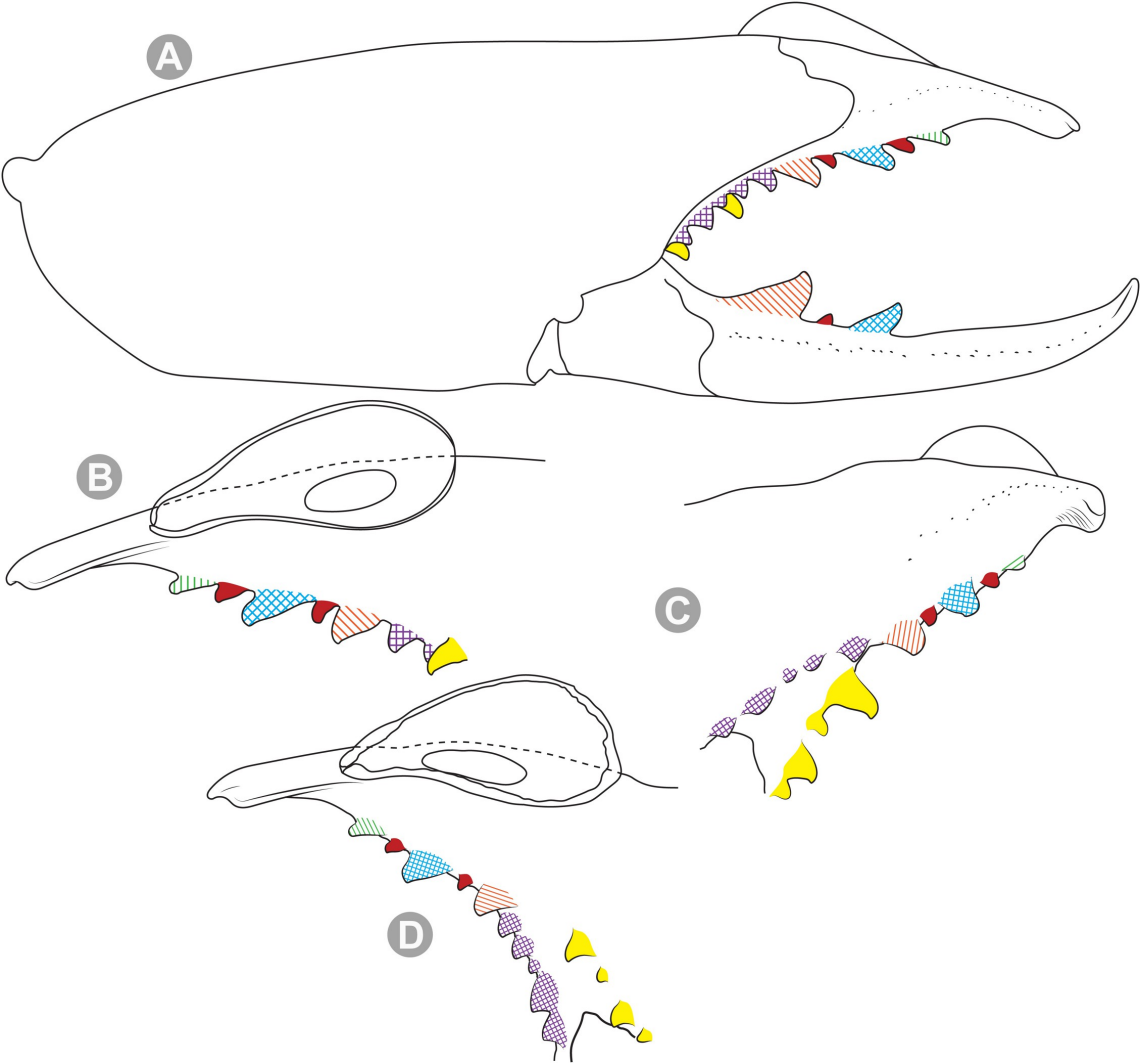
*Pseudocleobis puna* n. sp., schemes of male right chelicera. (A) Retrolateral view. (B–D) Fixed finger. (B) Prolateral view. (C) Retroventral view. (D) Proventral view.

Figs (5, 19 and 20 and Tables 1 and 2).

**Type material.** Holotype: CHILE: Atacama region: ♂, Copiapó province, Paso San Francisco, 20-I-2007, 4560 m. a.s.l.s, J. Pizarro-Araya coll (MNNC 8421). Paratype: CHILE: Atacama region: ♂, Copiapó province, Salar de Pedernales, 14-I-1972, 3300 m.a.l.s, collector unknown (MZUC 48063).

**Etymology.** The specific epithet *puna* is a noun in apposition taken from the Puna ecoregion, where this species occurs.

**Diagnosis.** Differs from *Pseudocleobis krausi* n. sp., *P. choros* n. sp., *P. lalackama* n. sp., *P. mumai* n. sp., *P. cekalovici* n. sp. and *P. escuadra* n. sp. by the long, straight prodorsal flange, and differs from *P. alticola* by the proventral flange not curved upward (Figs 19B, 19D and 20B). This species resembles *P. elongatus* n. sp. and *P. atacamensis* n. sp., in the mucron morphology (Figs 19B, 19D, 20B and 20D), and the flagellum pear-shaped (Figs 19B and 20B). It differs from both by the narrower flagellar groove, the lack of retroventral flange, and by the proventral flange that curves almost at the tip of the mucron.

**Description.** Male holotype: *Chelicerae*: Dentition and processes: fixed finger: median series with FD, 1 FSD (there is one additional, proximal, FSD teeth on left chelicera), FM, 1 FSM and FP teeth (Fig 20A–20D), FM slightly bigger than FM, is the biggest of fixed finger; retrofondal series with RFM, 2 RFSM (the proximal smaller), RFP, 1 RFSP teeth (Fig 20C and 20D) and profundal series with PFM, 1 PFSM, PFP, 1 PFSP teeth (Fig 20C and 20D). Fixed finger mucron relatively long, and ending in a very small, curved tip; flagellar groove narrow, with long, straight, parallel prodorsal and proventral flange; the proventral flange curves abruptly almost at the distal tip of the mucron (Figs 19B and 20B). Movable finger: with MM, 1 MSM and MP teeth (Fig 20A); MM similar in size as FM; MP is the biggest tooth of the chelicera. Movable finger mucron almost twice as long as fixed finger mucron, slightly curved; gnathal edge carina poorly elevated (Figs 19A, 19B and 20A). Stridulatory plate with 9 parallel ridges (Fig 19B). *Flagellum*: flagellum base pear-shaped (Figs 19B and 20B), narrowing distally, dorsal margin runs slightly convex to the apex, ventral margin has an evident concave curvature. The stalk (attachment ring) situated on the distal end of the prolateral setose area, longitudinally between FM and FP teeth (Fig 20B). *Pedipalps*: Femur with a proventral row of five long ventral spiniform setae, and a imperfectly aligned retroventral row of four lateroventral spiniform setae; the retroventral being smaller than the proventral and imperfectly paired with these. Tibia with 2.2.2.2.2 long ventral spiniform setae, the proximal pair setiform, the retroventral row smaller and more similar in size, the proventral row longer and arranged in increasing size I = V, II = IV, III. Basitarsus with 2.2.2.2 long ventral spiniform setae, the proximal pair setiform, the retroventral smaller than the proventral, but more equally sized in each row than those of the tibia. *Opisthosoma*: ctenidia present but hard to distinguish, 4–4 on sternite III and 2–2 on sternite IV.

**Female.** unknown.

**Variability.** The male paratype (MZUC 48063) has one tiny additional proximal MSM tooth on the right chelicera. Size variability in Table 2.

**Distribution and ecology.** This species is only known from two localities from the high altitudes of the Andes, in the Atacama region, with an altitude range of 3300–4560 m.a.s.l. The area where this species has been collected corresponds to the Desert Steppe of the Salares Andinos and High Andean Desert of Ojos del Salado botanical regions, formations which are characterized by the presence of large Andean salt flats and very sparse vegetation with some species of shrubs such as *Adesmia sentis* Phil. and plant associations such as *Atriplex deserticola* Phil.-*Lycium minutifolium* J. Remy, *Fabiana bryoides* Phil.-*Parastrephia lepidophylla* (Wedd.) Cabrera, *Atriplex imbricata* (Moq.) D. Dietr.-*Cristaria andicola* Gay, *Atriplex atacamensis* Phil.-*Tessaria absinthioides* (Hook. & Arn.) DC., and *Pappostipa chrysophylla* (E. Desv.) Romasch. [17].

Due to the extreme conditions where *Pseudocleobis puna* n. sp. occurs, this species presumably has low water requirements and is capable of withstanding wide thermal variations [17]. The specimen from Paso San Francisco was collected during the day, under a stone. Like all the other species of the genus, it is presumably nocturnal, preying on small arthropods.

**Fig 21:**
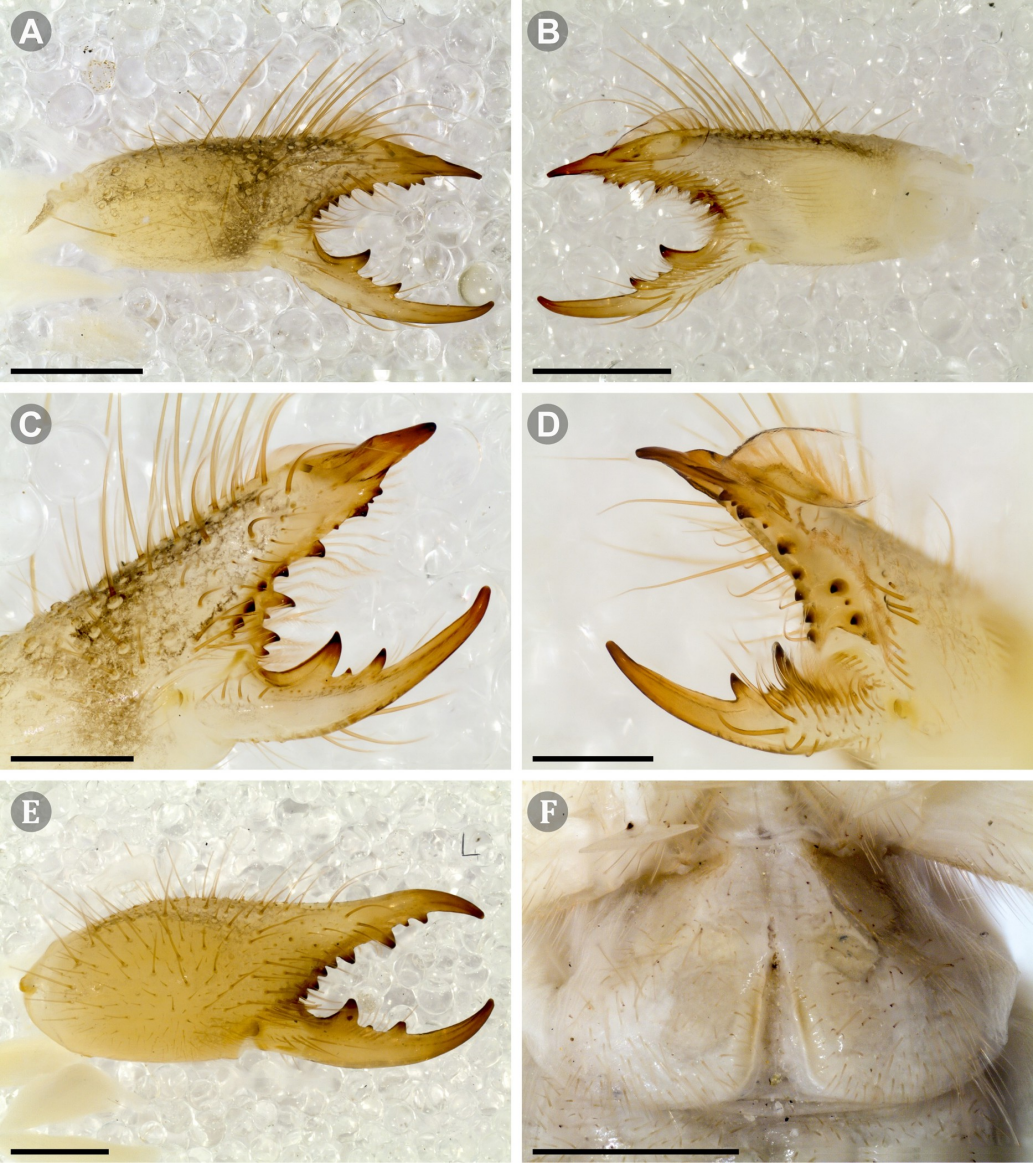
*Pseudocleobis krausi* n. sp., male holotype, right chelicera. (A) Retrolateral view. (B) Prolateral view. (C) Cheliceral fingers, retroventral view. (D) Cheliceral fingers, proventral view. Scale bars: 1 mm (A–B); 0,5 mm (C–D).

**Fig 22:**
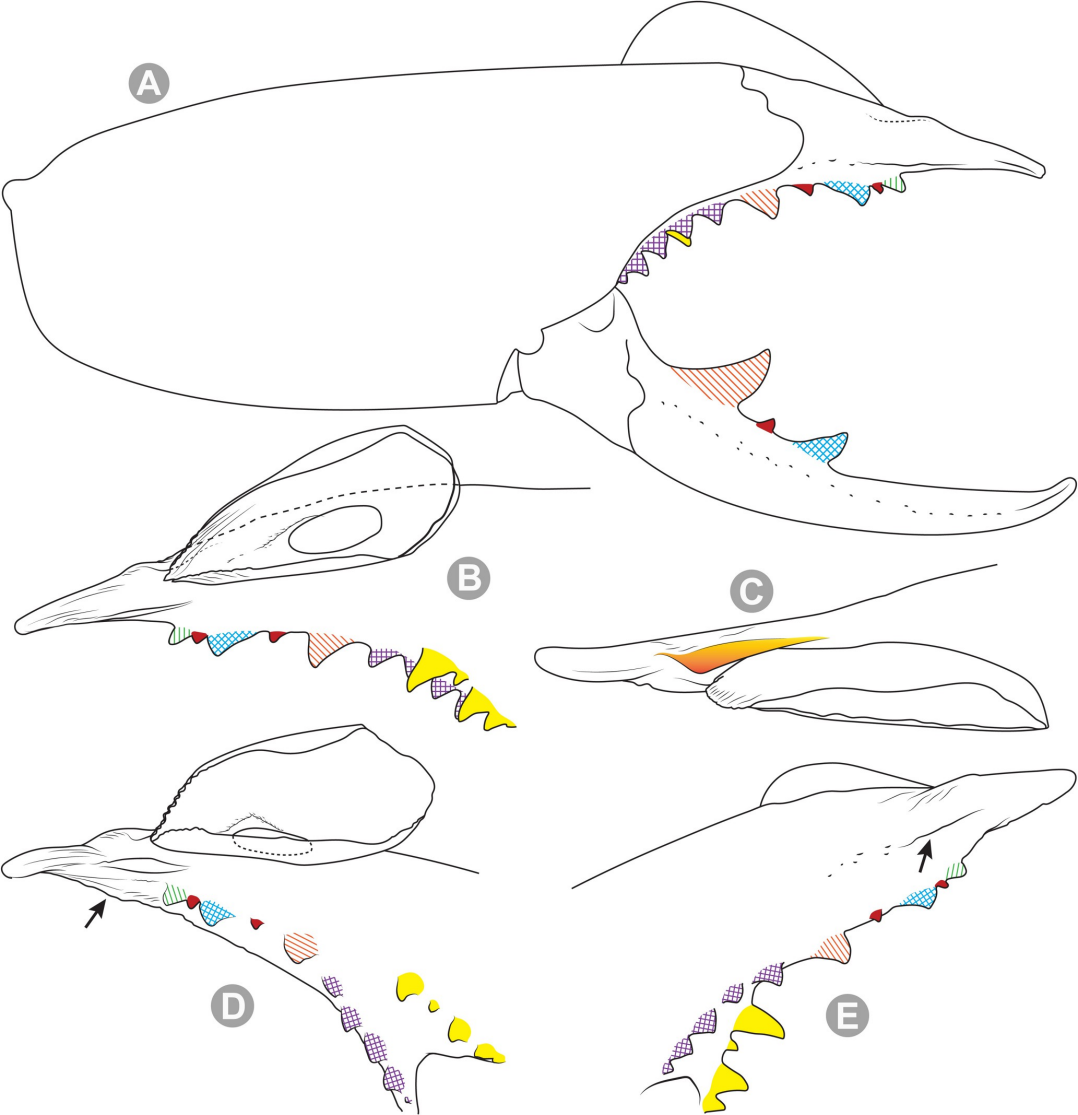
*Pseudocleobis krausi* n. sp., schemes of male right chelicera. (A) Retrolateral view. (B–E) Fixed finger. (B) Prolateral view. (C) Dorsal view (prodorsal flange orange colored). (D) Proventral view. (E) Retroventral view. Arrows in D and E indicates the retroventral flange.

***Pseudocleobis krausi* Iuri & Maury n. sp.** urn:lsid:zoobank.org:act:C7685379-D826-4B03-B900-DF528EE1F98A.

(Figs 2C, 5, 21 and 22; Table 3).

**Table 3:** Metric data for *Pseudocleobis krausi* n. sp., *P. choros* n. sp. and *P. lalackama* n. sp. Measurements in millimeters for male holotype and one female (when known).

		*P. krausi* n. sp.		*P. choros* n. sp.		*P. lalackama* n. sp.
		♂ (holotype)	♀	♂ (holotype)	♀	♂ (holotype)
Total body	L^1^	9,47	10,8	8,5	11,7	6,74
Propeltidium	L	1,78	1,83	1,62	1,48	1,25
	W	2,11	3,16	1,97	2,52	1,84
Chelicera	L^2^	3,23	4,5	2,78	3,81	2,37
	W^3^	0,98	1,54	0,81	1,14	0,71
	H^4^	1,04	1,66	0,87	1,24	0,77
Pedipalp	L^5^	14,42	11,42	13,35	8,24	11,18
	femur L	5,35	3,93	5,03	2,76	4,15
	tibia L	5,29	4,28	4,77	3,11	4,15
	basitarsus L	2,98	2,43	2,74	1,56	2,22
	telotarsus L	0,8	0,78	0,81	0,81	0,66
Leg I	L^5^	11,53	8,2	10,58	5,88	8,58
	patela L	4,63	2,84	4	2,22	3,28
	tibia L	3,91	3,15	3,79	1,73	2,96
	basitarsus L	2,09	1,59	1,91	1,38	1,62
	telotarsus L	0,9	0,62	0,88	0,55	0,72
Leg IV	L^5^	17,14	15,09	15,93	13,08	13,52
	patela L	5,54	4,78	5,23	4,23	4,4
	tibia L	5,33	4,69	5,26	4,1	4,35
	basitarsus L	4,35	3,51	3,74	3,15	3,16
	telotarsus L^6^	1,92	2,11	1,7	1,6	1,61

L = length; W = width; H = height.

^1^ Measured along medial axis, from the propeltidium anterior margin to the opisthosoma posterior margin.

^2^ Measured in retrolateral view from chelicero-peltidial condyle to the tip of fixed finger mucron.

^3^ Measured in dorsal view at widest point.

^4^ Measured in retrolateral view at highest point of the manus.

^5^ Sum of individual segments length.

^6^ Measurement excludes claws.

**Female:** morphology and chaetotaxy like males, except by the wider propeltidium, wider chelicerae, unmodified mucron (Fig 21E) and shorter legs and pedipalps. The chelicera exhibits an anomalous dentition, right chelicera (Fig 21E) lacks FSM tooth being a gap in his place, and there is also a gap between the FM and FSD teeth; left chelicera has the FSM tooth, but lacks FSD and FD teeth. The spiniform setae of pedipalps are more spiniform than in males. Opisthosomal sternite III and IV without ctenidia. Genital plate (Fig 21F) bell-shaped, with lateral margins concave at distal half, and convex at proximal half; has a long middle cuticular opening as an inverted V; the two halves don’t touch each other posteriorly.

*Pseudocleobis andinus* (Pocock): Kraus, 1966 [8]: 182 (misidentification in part).

**Type material.**: Holotype: CHILE: Atacama region: ♂, Copiapó province, Boquerón Chañar, 28°16’09.2’’S 70°31’08.7’’, 697 m. a.s.l., 24-25-XI-2022, H.A. Iuri, A.A. Ojanguren-Affilastro, J. Pizarro-Araya, F.M. Alfaro, J.E. Calderón & J.E. Barriga-Tuñón coll (MNNC 8422). Paratype: CHILE: Atacama region: 1♀, same data as holotype (MNNC 8423).

**Other material.** CHILE: Atacama region: ♂, Copiapó province, Travesía, 26-VIII-1963, F. di Castri (SMF 17374; examined by Maury, unpublished drawings and description at MACN; S2 Fig).

**Etymology.** The specific epithet is given in honor to Dr. Otto Kraus (1930–2017), German zoologist who published the first revisionary work on Chilean solifuge fauna.

**Diagnosis.** Differs from all known *Pseudocleobis*, except *P. andinus*, *P. choros* n. sp., *P. lalackama* n. sp. and *P. elongatus* n. sp., by having a retroventral flange on male fixed finger mucron (Figs 21D and 22D and 22E). Differs from *P. elongatus* n. sp. by the short prodorsal flange (Fig 22B and 22C), and from *P. andinus* by the prodorsal flange curved downwards, and the proventral flange not curved upward (Fig 22B). This species resembles *P. choros* n. sp. and *P. lalackama* n. sp. in the morphology of the male fixed finger mucron with a retroventral flange (Fig 22D) and the teardrop-shaped flagellum with a middle line of small, pointed projections on distal half of prolateral surface. Differs from both by having a poorly extended retroventral flange on fixed finger (not hiding part of the gnathal edge carina in retrolateral view) (Figs 21D and 22). The prodorsal flange of the flagellar groove extends beyond the FD more than in *P. lalackama* n. sp., but less than in *P. choros* n. sp.

**Description.** Male: Measurements in Table 1. *Chelicerae*: Dentition and processes: fixed finger: median series with FD, 1 FSD, FM, 1 FSM and FP teeth (Fig 22A), FSD tooth tiny and partially fused at the base with the FD and FM teeth (Figs 21A, 21B, 22A and 22B); FP and FM similar sized, are the biggest teeth on fixed finger; retrofondal series with RFM, 1 RFSM, RFP, 1 RFSP teeth (Fig 22D and 22E) and profondal series with PFM, 1 PFSM, PFP, 1 PFSP teeth (Fig 22D and 22E). Fixed finger mucron relatively long, and narrowly ended, with a retroventral flange poorly developed and not expanded downwards (Fig 22A–22C and 22E); flagellar groove wide, with short prodorsal flange that ends in the basal third of the mucron, and a long proventral flange that runs almost straight to the tip of the mucron (Figs 21B and 22B). Movable finger: with MM, 1 MSM and MP teeth (Fig 22A); MM bigger than FM and FP; MP is the biggest tooth of the chelicera. Movable finger mucron approx. 1.5 times as long as fixed finger mucron (Fig 22A), slightly curved; gnathal edge carina poorly elevated (Fig 22A). Stridulatory plate with 9 parallel ridges. *Flagellum*: flagellum base teardrop-shaped, narrowed distally, with the dorsal margin running concave till the anterior end, and the ventral margin is almost straight (Figs 21B and 22B). The stalk (attachment ring) situated on the distal end of the prolateral setose area, longitudinally between FSM and RFM teeth (Fig 22B). There is a distal middle row of irregular projections on prolateral surface (Fig 22B). *Pedipalps*: Femur with a proventral row of four long ventral spiniform setae and two retroventral imperfectly paired with the proventral. Tibia with 2.2.2.2.2 long ventral spiniform setae, proximal pair weaker, retroventral row smaller and more similar in size. Basitarsus with 2.2.2.2 long lateroventral spiniform setae, the proximal pair setiform, the distal pair smaller. *Opisthosoma*: tergites and sternites with bifurcated tip setae. Ctenidia present but hard to distinguish, 3–3 on sternite III and 3–3 on sternite IV.

**Variability:** The female has two additional small retroventral spiniform seta on tibia of left pedipalp, between subbasal and medial ones (Fig 8D).

**Distribution and ecology.** This species is only known from two localities of intermediate altitudes in Copiapó province, Atacama region, areas that correspond to the Flowering Desert of the Llanos and Flowering Desert of the mountain ranges botanical regions [17]. The area where this species has been collected is an isolated relict forest of *Neltuma chilensis* in an area dominated by shrub-steppe, with a dominance of *Heliotropium stenophyllum* Hook. & Arn., *Oxalis gigantea* Barnéoud, *Encelia tomentosa* Walp. and *Nolana paradoxa* Lindl [17].

Like *Pseudocleobis atacamensis* n. sp., our collections were made after the flowering desert phenomenon, the peak instance of the biological expression in the Atacama Desert, which allowed us to record a fauna that is usually difficult to collect, since it is inactive for long periods. This species was found in sympatry with *Ammotrechelis* sp. and *Chileotrecha* sp. Additionally, the species *P. atacamensis* n. sp., a species of *Chileotrecha*, and, at least, three species of Mummucidae were found in close areas to the collection site.

**Fig 23:**
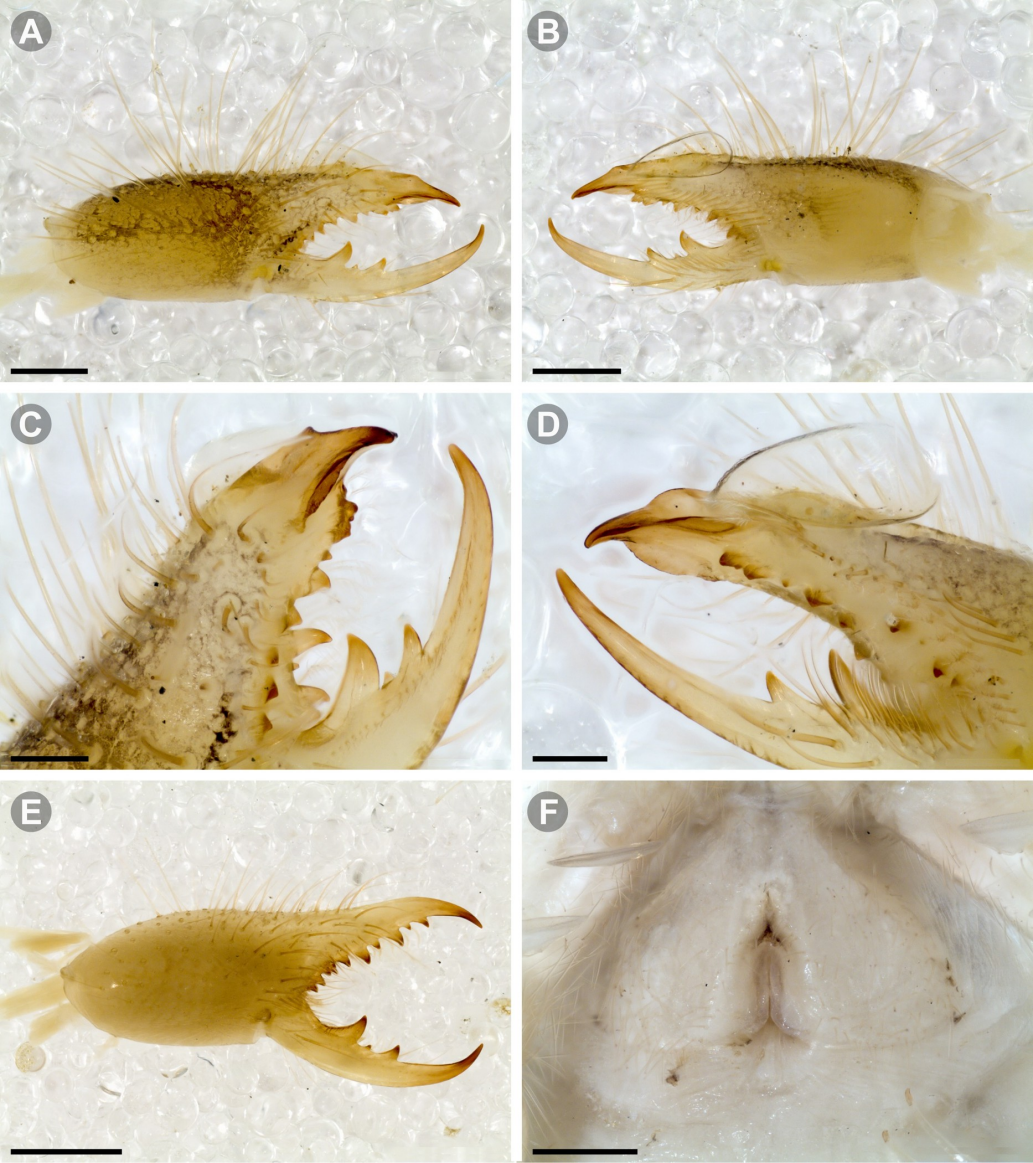
*Pseudocleobis choros* n. sp. (A–D) Male holotype, right chelicera. (A) Retrolateral view. (B) Prolateral view. (C) Cheliceral fingers, retroventral view. (D) Cheliceral fingers, proventral view. (E–F). Female paratype. (E) Right chelicera, retrolateral view. (F) Genital sternite, ventral view. Scale bars: 1 mm (A–B, E); 0,5 mm (C–D, F).

**Fig 24:**
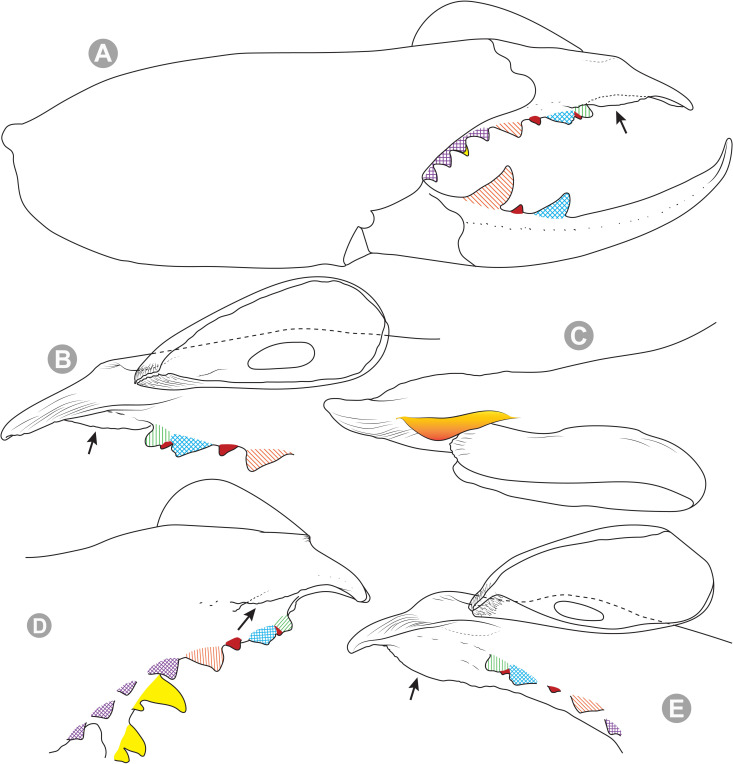
*Pseudocleobis choros* n. sp., schemes of male right chelicera. (A) Retrolateral view. (B–E) Fixed finger. (B) Prolateral view. (C) Dorsal view (prodorsal flange orange colored). (D) Retroventral view. (E) Proventral view. Arrows in A, B, D and E indicates the retroventral flange.

***Pseudocleobis choros* Iuri & Maury n. sp.** urn:lsid:zoobank.org:act:ADAFA668-5C52-4104-838A-E1AB391ACB27.

(Figs 5, 23 and 24 and Tables 3 and 4).

**Table 4:** Variability on metric data for *Pseudocleobis choros* n. sp., *P. lalackama* n. sp. Values in millimeters.

		*P. choros* n. sp.			*P. lalackama* n. sp.		
		female (n = 3)			male (n = 3)		
		MIN	MEAN	MAX	MIN	MEAN	MAX
Total body	L^1^	10,50	11,00	11,70	6,07	6,54	6,79
Propeltidium	L	1,48	1,69	1,80	1,25	1,31	1,37
	W	2,43	2,50	2,54	1,55	1,71	1,84
Chelicera	L^2^	3,68	3,75	3,81	2,04	2,28	2,43
	W^3^	1,14	1,18	1,24	0,67	0,70	0,73

L = length; W = width; H = height.

^1^ Measured along medial axis, from the propeltidium anterior margin to the opisthosoma posterior margin.

^2^ Measured in retrolateral view from chelicero-peltidial condyle to the tip of fixed finger mucron.

^3^ Measured in dorsal view at widest point.

*Pseudocleobis andinus* (Pocock): Kraus, 1966 [8]: 182 (misidentification in part).

**Type material.** Holotype: CHILE: Coquimbo region: ♂, Elqui province, Los Choros, González & Loos coll., (MNNC 8424). Paratype: CHILE: Coquimbo region: 1♀, Elqui province, Punta Choros (MNNC 8425); 1♀, Elqui province, Punta Choros, (MACN-Ar 46524); 1♀, Elqui province, Los Choros, 19-X-2008, J. Pizarro-Araya coll., (LEULS 189).

**Other material.** CHILE: Coquimbo region: 1♂, 1♀, Elqui province, El Tofo, 27-VIII-1963, F. di Castri (SMF 17367; examined by Maury, unpublished drawings at MACN; S3 Fig).

**Etymology.** The specific epithet *choros* refers to the type locality Los Choros, coastal town, which is located in front of the Reserva Nacional Pingüino de Humboldt.

**Diagnosis.** Differs from all known *Pseudocleobis*, except *P. andinus*, *P. krausi* n. sp., *P. lalackama* n. sp. and *P. elongatus* n. sp., by having a retroventral flange on male fixed finger mucron (Figs 23A–23D, 24B, 24D and 24E). Differs from *P. elongatus* n. sp. by the short prodorsal flange (Figs 24B and 24C), and from *P. andinus* by the prodorsal flange curved downwards and the proventral flange not curved upward (Fig 24B). This species resembles *P. krausi* n. sp. and *P. lalackama* n. sp. in the morphology of male fixed finger mucron with a retroventral flange and the teardrop-shaped flagellum with a middle line of projections on distal half of prolateral surface. Differs from both by having a greatly expanded retroventral flange (Fig 24D and 24E), the flagellar groove wider and the prodorsal flange extending much beyond the FD tooth (Fig 24A and 24B).

**Description.** Measurements in Table 3. Male holotype: *Chelicerae*: Dentition and processes: fixed finger: median series with FD, 1 FSD (absent in left chelicera), FM, 1 FSM and FP teeth (Fig 24A); FSM small, FSD vestigial; FD, FSD and FM fused at the base (Fig 24A and 24B); FP is the biggest tooth of fixed finger; retrofondal series with RFM, 1 RFSM, RFP, 1 RFSP teeth (Fig 24A and 24D) and profondal series with PFM, 1 PFSM, PFP, 1 PFSP teeth (Fig 24D). Fixed finger mucron long and wide in the proximal half, with a pronounced retroventral flange expanding downwards and covering part of the gnathal edge carina in lateral view (Fig 24A), and a wide prolateral flagellar groove; prodorsal flange extends beyond FD reaching almost the half of the mucron (Fig 24A and 24B). Movable finger: with MM, 1 MSM and MP teeth (Fig 24A); MM bigger than FP; MP is the biggest tooth of the chelicera; movable finger mucron almost twice as long as fixed finger mucron (Figs 23A, 23B and 24A), slightly curved; gnathal edge carina poorly elevated and not forming a convex bulge; the tip is distinctly curved upward (Figs 23A, 23B and 24A). Stridulatory plate with 9 parallel ridges. *Flagellum*: flagellum base teardrop-shaped, narrowing distally; dorsal margin of the base runs convex to distal, while the ventral margin is almost straight (Figs 23B and 24B). There is a distal middle row of irregular projections on prolateral surface (Fig 24B). The stalk (attachment ring) situated on the distal end of the prolateral setose area, longitudinally between FSM and FP teeth (Fig 24B). *Pedipalps*: Femur with a proventral row of five long lateroventral spiniform setae, and a imperfectly aligned retroventral row of four lateroventral spiniform setae; the retroventral smaller than the proventral and imperfectly paired with these. Tibia with 2.2.2.2.2 long ventral spiniform setae, the proximal pair weaker, the retroventral row smaller and more similar in size; there is a small additional spiniform retroventral seta between the medial and the subdistal (Fig 8C). Basitarsus with 2.2.2.2 long ventral spiniform setae, proximal pair setiform, distal pair smaller. *Opisthosoma*: Ctenidia present as thick setae, some of them seem to be broken but insertions are visible, 5–5 on sternite III and 4–4 on sternite IV.

**Female.** Morphology and chaetotaxy like males, except by the wider propeltidium, unmodified chelicerae (Fig 23E), and shorter legs and pedipalps. The spiniform setae of pedipalps are more spiniform than in males. Opisthosomal sternite III and IV without ctenidia. Genital plate (Fig 23F) bell-shaped, with lateral margins slightly concave at distal half, and convex at proximal half; has a long middle cuticular opening, as an inverted U, with two cuticular extensions covering the genital opening; the two halves don’t touch each other posteriorly.

**Variability:** One female has two additional small retroventral spiniform setae on the right pedipalp, between the medial and subdistal ones (Fig 8B). Size variability in Table 4.

**Distribution and ecology:** We have only recorded *Pseudocleobis choros* n. sp. in coastal areas close to the village of Los Choros (Elqui Province, Coquimbo Region), belonging to the Huasco coastal desert botanical region [17].

This species has been collected in coastal dunes and stabilized paleodunes. *Pseudocleobis choros* n. sp. inhabits sandbanks with sparse vegetation, generally *Crystaria glaucophyla* Cav., *Zephyranthes bagnoldii* (Herb.) and *Tetragonia maritima* Barn.

Only a few records are known for this species, although our research in the area has been going on for more than 15 years. The specimens collected were associated with paleodunes with some isolated rocky substrata, where they were collected during the day, although like all the species of the genus they are presumably nocturnal.

**Fig 25:**
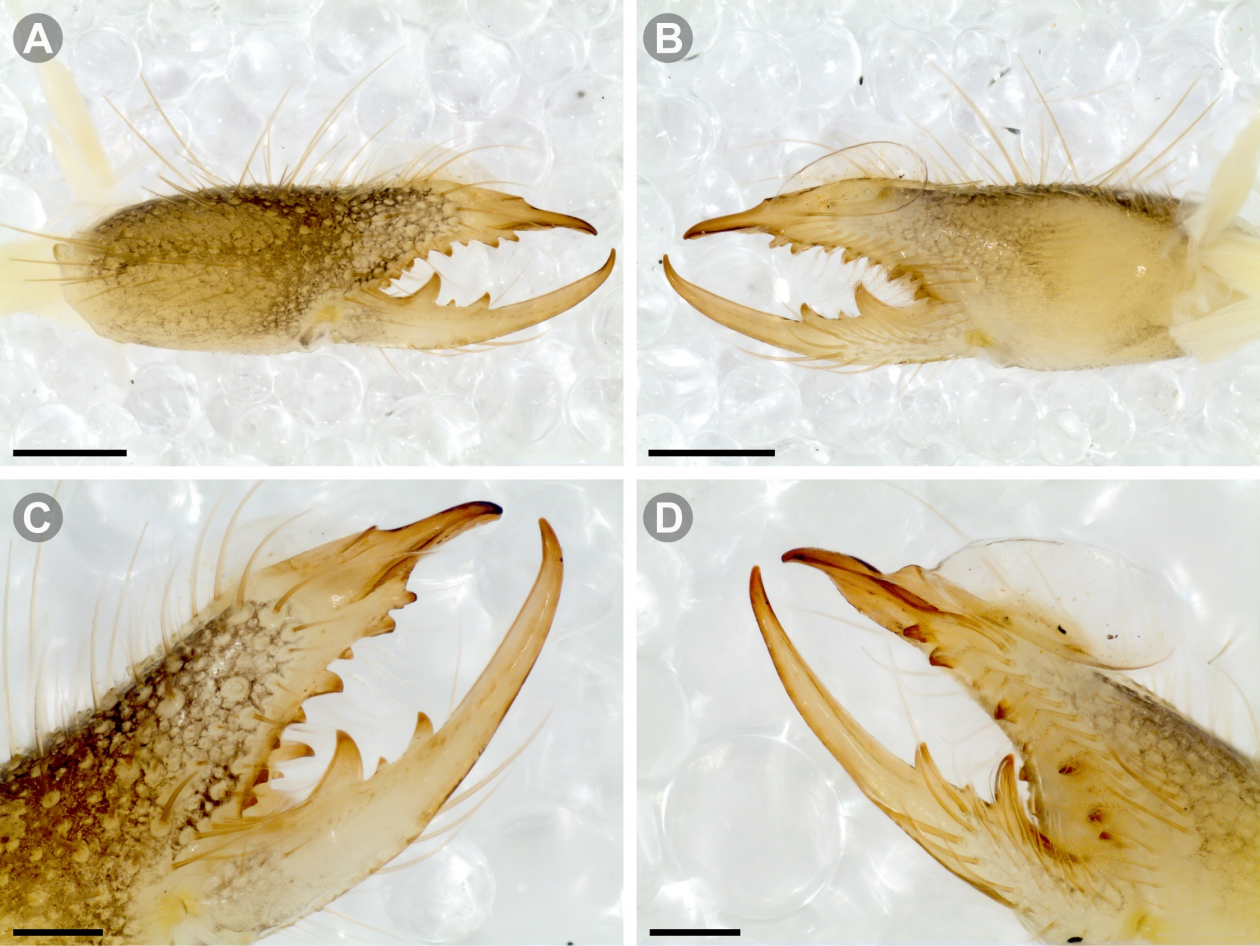
*Pseudocleobis lalackama* n. sp., male holotype, right chelicera. (A) Retrolateral view. (B) Prolateral view. (C) Fixed finger, retroventral view. (D) Fixed finger, proventral view. Scale bars: 0,5 mm (A–B); 0,2 mm (C–D).

**Fig 26:**
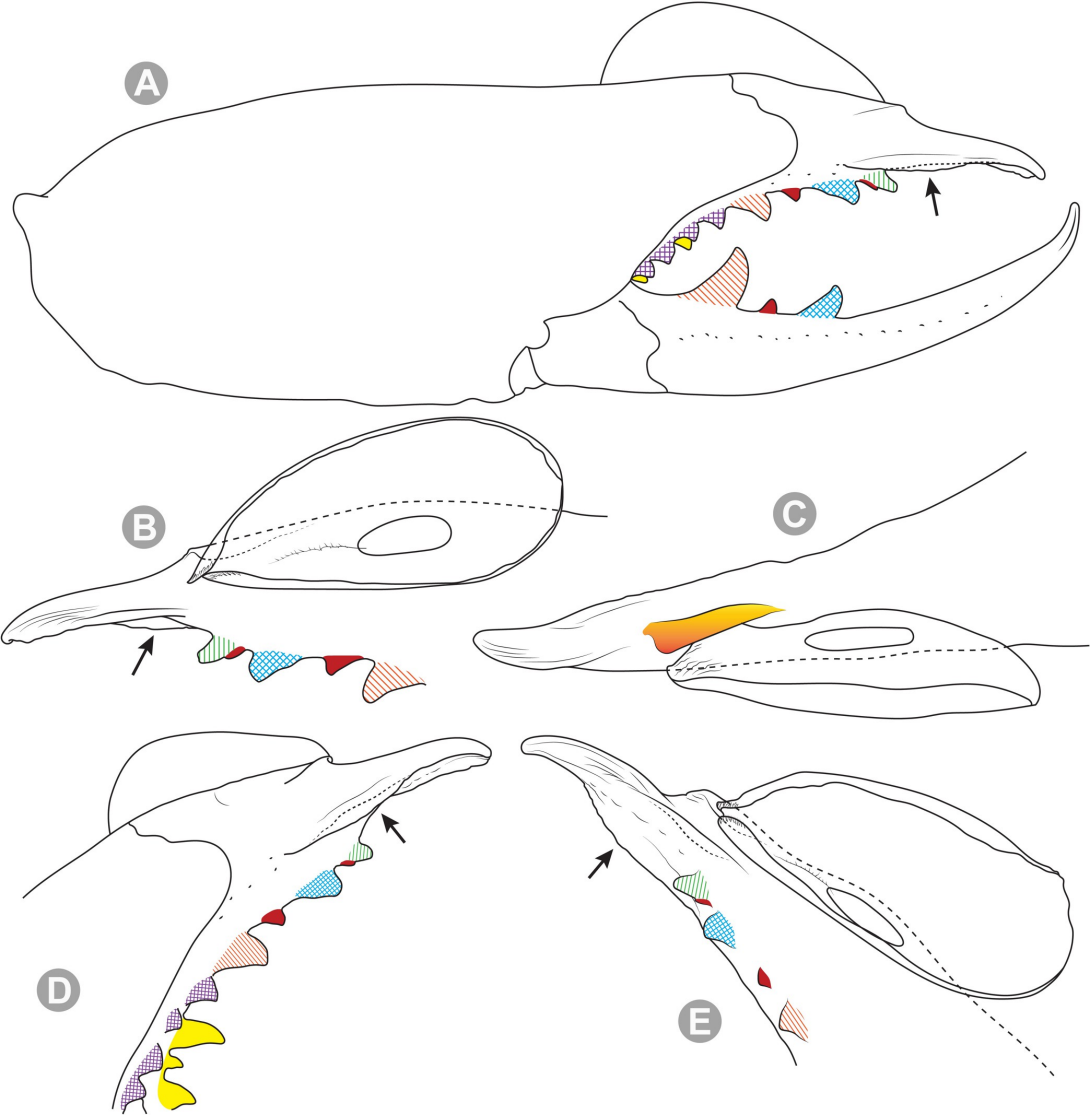
*Pseudocleobis lalackama* n. sp., schemes of male right chelicera. A. Retrolateral view. (B–E) Fixed finger. (B) Prolateral view. (C) Dorsal view (prodorsal flange orange colored). (D) Retroventral view. (E) Proventral view. Arrows in A, B, D and E indicates the retroventral flange.

**Fig 27:**
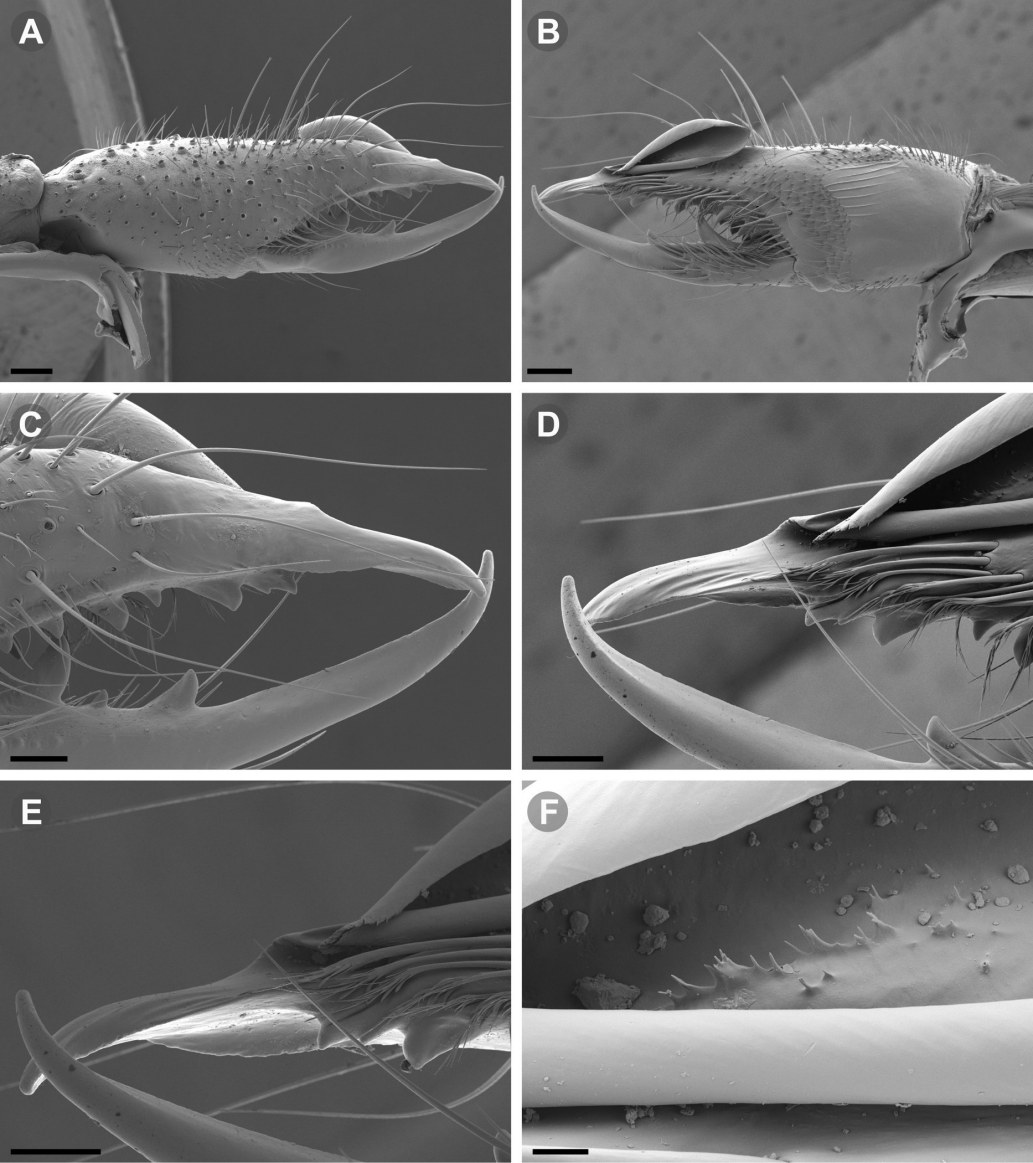
*Pseudocleobis lalackama* n. sp., SEM images of male right chelicera. (A) Retrolateral view. (B) Prolateral view. (C) Cheliceral fingers, retrolateral view. (D) Cheliceral fingers, prolateral view. (E) Fixed finger mucron, proventral view. (D) Flagellum prolateral surface, detail of distal area. Scale bars: 0,2 mm (A–B), 0,1 mm (C–E), 0,02 mm (D).

***Pseudocleobis lalackama* Iuri n. sp.** urn:lsid:zoobank.org:act:3DC1657F-D0E5-4489-A638-EAE6BEF9CCC9.

(Figs 2A, 5 and 25–27 and Tables 3 and 4).

**Type material.** Holotype: CHILE: Atacama region: ♂, Chañaral province, Pan de Azúcar National Park, Las Lomitas, 26°06’42.39’’S 70°38’47.65’’W, F.M. Alfaro, J. Pizarro-Araya coll., (MNNC 8426). Paratypes: CHILE: Antofagasta region: 1♂, Antofagasta province, Paposo, Quebrada Los Yales, 25°00’13.7’’S 70°25’52.6’’W, 891 m. a.s.l., 16-XI-2022, J. Pizarro-Araya, F.M. Alfaro & J.E. Calderón coll., (MACN-Ar 46525); 1♂, Antofagasta province, Paposo, cabaña CONAF, 25°00’24.90’’S 70°27’0.53’W, 700 m. a.s.l., 26-29-X-2015, R. Botero-Trujillo, J. Pizarro-Araya, J.E. Barriga-Tuñón, F.M. Alfaro coll., (LEULS 190).

**Etymology.** The specific epithet lalackama from Kunza (an extinct language spoken in the Atacama Desert of northern Chile and southern Peru by the Atacama people), lalackama = dawn, light up; Lalcktchi = light, lalcktur = dawn.

**Diagnosis.** Differs from all known *Pseudocleobis*, except *P. andinus*, *P. krausi* n. sp., *P. choros* n. sp. and *P. elongatus* n. sp., by having a retroventral flange on male fixed finger mucron (Figs 25, 26A, 26B, 26D and 26E). Differs from *P. elongatus* n. sp. by the short prodorsal flange (Fig 26B and 26C), and from *P. andinus* by the prodorsal flange curved downwards and the proventral flange not curved upward (Fig 26B). This species resembles *P. krausi* n. sp. and *P. choros* n. sp. in the morphology of male fixed finger mucron with a retroventral flange and the teardrop-shaped flagellum with a middle line of projections on distal half of proventral surface (Fig 27E). Differs from both by the prodorsal flange of flagellar groove, that barely extends beyond the FD tooth (Figs 26B and 27D). The retroventral flange (Figs 26D, 26E and 27E) is more pronounced than *P. krausi* n. sp., but less than *P. choros* n. sp.

**Description.** Measurements in Table 3. Male holotype: *Chelicerae*: Dentition and processes: fixed finger: median series with FD, 1 FSD, FM, 1 FSM and FP teeth (Fig 26A); FSD tooth vestigial, strongly fused with FD tooth; FD, FSD and FM fused at the base; FP tooth similar size as FM tooth; retrofondal series with RFM, 1 RFSM (there is one additional posterior, tiny RFSM tooth on left chelicera), RFP, 1 RFSP teeth (Figs 26A and 26D) and profondal series with PFM, 1 PFSM, PFP, 1 PFSP teeth (Fig 25B and 25D. Fixed finger mucron long, wide at proximal fourth but narrow most of its extension to distal; retroventral flange expanding downwards, covering part of the gnathal edge carina in lateral view (Fig 26A). Flagellar groove with short prodorsal flange barely extending beyond FD tooth (Figs 25A, 26B, 26C and 27D). Movable finger: with MM, 1 MSM and MP teeth (Fig 25A); MM bigger than FP; MP is the biggest tooth of the chelicera; movable finger mucron approx. 1.5 times as long as fixed finger mucron (Figs 25A, 25B, 26A and 27A–27C), slightly curved; gnathal edge carina poorly elevated and not forming a convex bulge; mucron tip distinctly curved upward (Figs 25A–25C, 26A and 27A–27D). Stridulatory plate with 9 parallel ridges (Figs 25B and 27B). *Flagellum*: flagellum base teardrop-shaped (Figs 25B and 26B), narrowing distally; dorsal margin runs convex to distal, whilst the ventral margin is almost straight. There is a distal middle row of irregular projections on prolateral surface (Figs 26B and 27F). The stalk (attachment ring) situated on the distal end of the prolateral setose area, longitudinally between FSM and RFM teeth (Figs 25B and 26B). *Pedipalps*: Femur with a proventral row of five long lateroventral spiniform setae, and a imperfectly aligned retroventral row of four lateroventral spiniform setae; the retroventral smaller than the proventral and imperfectly paired with these. Tibia with 2.2.2.2.2 long ventral spiniform setae, proximal pair setiform, retroventral row smaller and more similarly sized; there is a small additional spiniform retroventral seta between the medial and the subdistal (Fig 6D). Basitarsus with 2.2.2.2 long ventral spiniform setae, proximal pair setiform, distal pair smaller. *Opisthosoma*: Ctenidia present as thick setae, 2–2 on sternite III and 2–1 on sternite IV.

**Female.** unknown.

**Variability:** No variability on discrete characters. Size variability in Table 4.

**Distribution and ecology.** The area where this species has been collected belongs to the Taltal coastal desert and interior coastal desert botanical regions [17]. This species has only been collected in coastal environments, in areas with substrate formed mostly by sand, and with some small rocks, and associated with cacti as *Copiapoa cinerea* (Phil.) Britton & Rose, *Eulychnia iquiquensis* (K. Schum.) Britton & Rose, shrubs as *Lycium deserti* Phil., *Euphorbia lactiflua* Phil. and the annual herb *Cristaria integerrima* Phil.

The localities where this species has been collected belong to the "Lomas" environment, which have a markedly more humid microclimate than the surrounding desert, due to the fact that they are under the influence of marine fog (camanchaca), thus allowing the presence of a much more diverse and developed vegetation [41, 42].

The only known specimens of *Pseudocleobis lalackama* n. sp. have been collected in spring, which suggests that this species has a spring-summer activity period, as most known species of *Pseudocleobis*.

**Fig 28:**
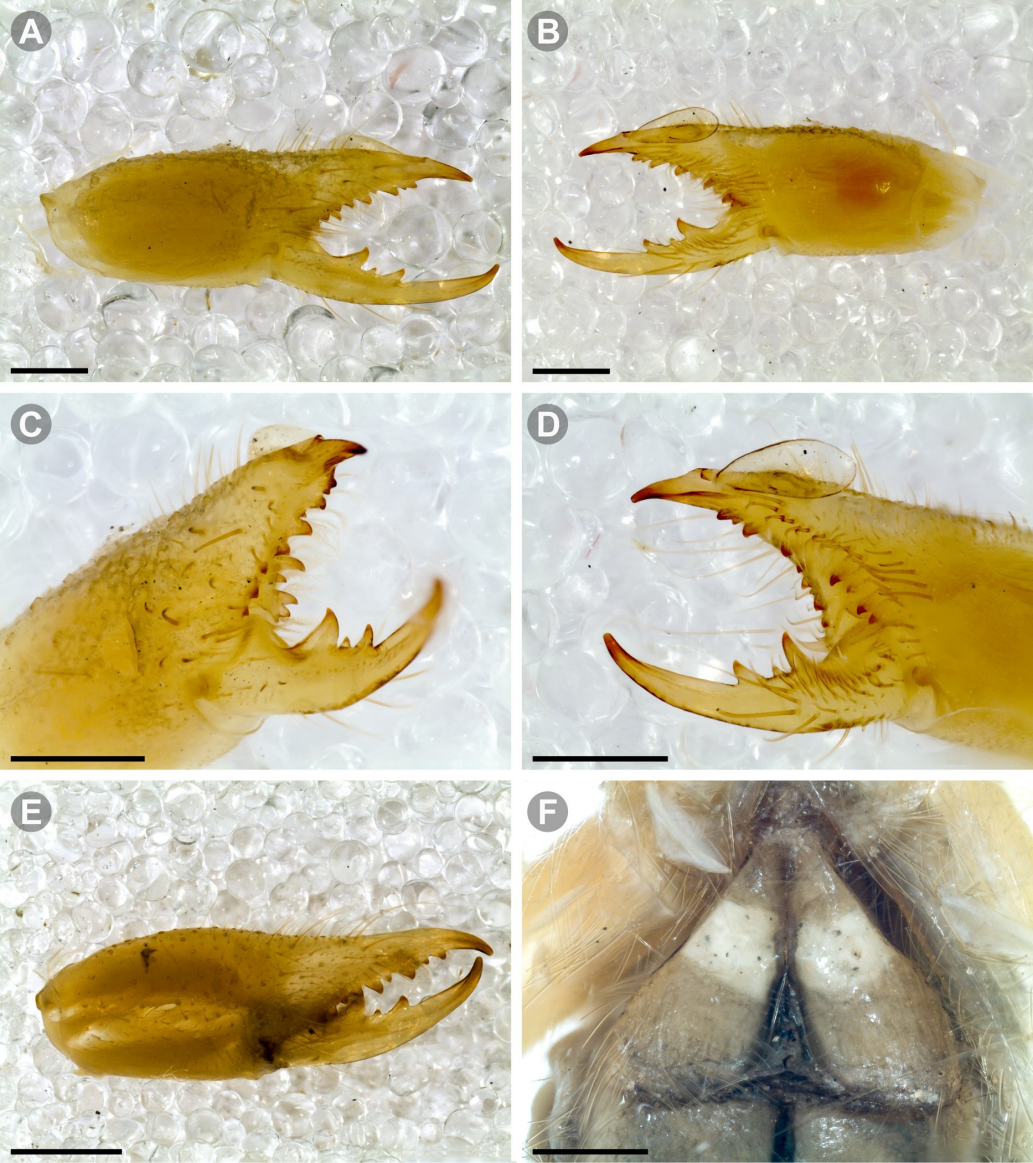
*Pseudocleobis mumai* n. sp. (A–D) Male holotype, right chelicera. (A) Retrolateral view. (B) Prolateral view. (C) Fixed finger, retroventral view. (D) Fixed finger, proventral view. (E–F). Female paratype. (E) Right chelicera, retrolateral view. (F) Genital sternite, ventral view. Scale bars: 1 mm (E); 0,5 mm (A–D, F).

**Fig 29:**
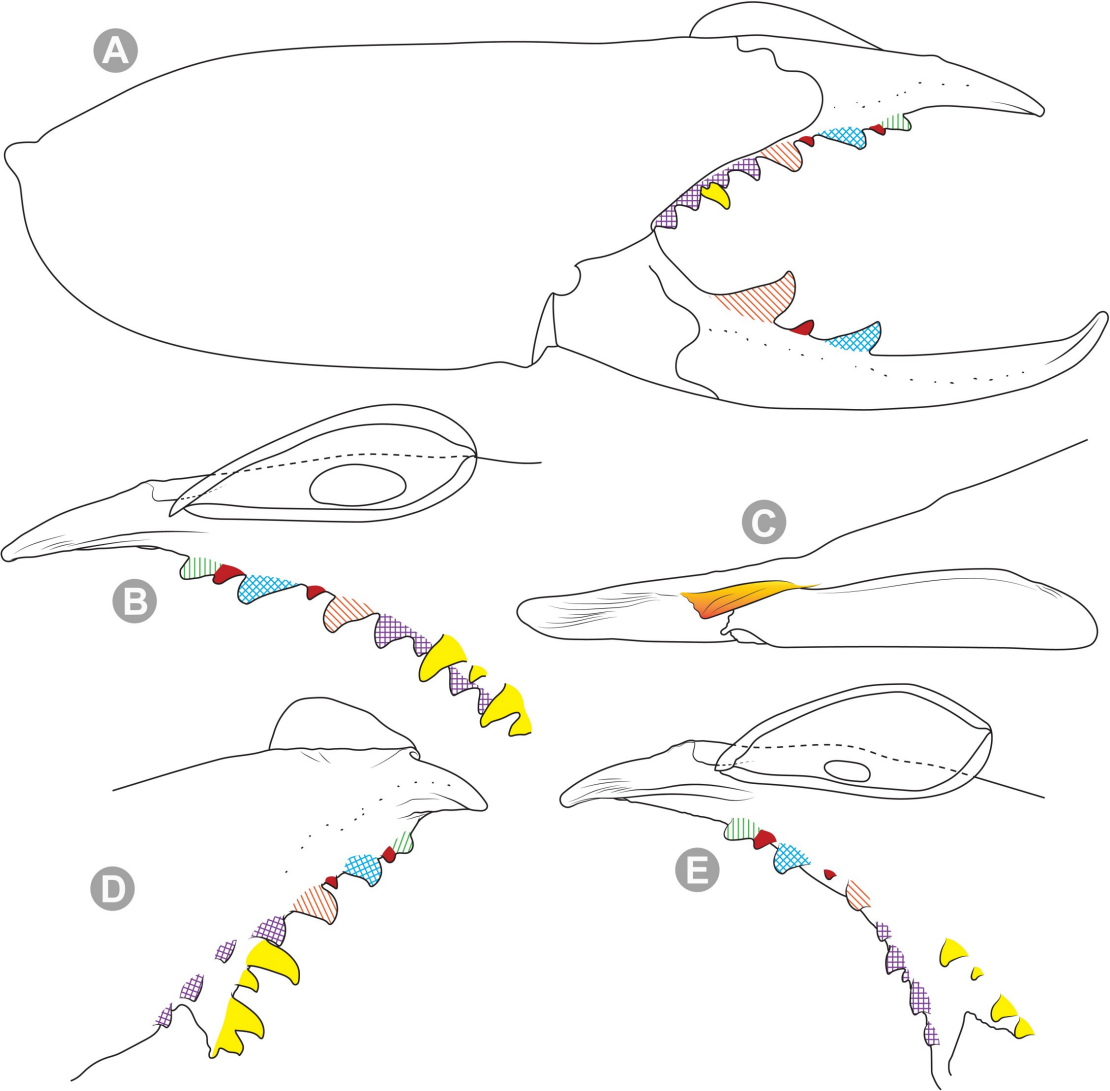
*Pseudocleobis mumai* n. sp., schemes of male right chelicera. (A) Retrolateral view. (B–E) Fixed finger. (B) Prolateral view. (C) Dorsal view (prodorsal flange orange colored). (D) Retroventral view. (E) Proventral view.

***Pseudocleobis mumai* Iuri n. sp.** urn:lsid:zoobank.org:act:7F4E15BA-68BC-426A-9DB2-1D428EDF044A.

(Figs 5, 28 and 29 and Tables 5 and 6).

**Table 5:** Metric data for *Pseudocleobis mumai* n. sp., *P. cekalovici* n. sp. and *P. escuadra* n. sp. Measurements in millimeters for male holotype and one female (when known).

		*P. mumai* n. sp.		*P. cekalovici* n. sp.		*P. escuadra* n. sp.	
		♂ (holotype)	♀	♂ (holotype)	♀	♂ (holotype)	♀
Total body	L^1^	8,41	7,38	9,34	8,66	9,54	8,88
Propeltidium	L	1,62	1,68	1,42	1,77	1,71	1,44
	W	1,95	2,88	1,97	2,74	2,37	2,25
Chelicera	L^2^	2,75	4,25	2,85	3,87	3,33	3,13
	W^3^	0,81	1,34	0,82	1,16	0,95	0,9
	H^4^	0,89	1,42	0,88	1,27	1,02	0,96
Pedipalp	L^5^	13,95	12,5	12,15	11,09	15,08	8,84
	femur L	5,14	4,61	4,48	3,93	5,86	3,16
	tibia L	5,04	4,61	4,24	4,02	5,33	3,14
	Basitarsus L	2,93	2,62	2,68	2,37	3	1,77
	telotarsus L	0,84	0,66	0,75	0,77	0,89	0,77
Leg I	L^5^	9,65	8,65	8,96	7,71	11,23	6,56
	patela L	3,56	2,91	3,25	2,43	4,04	2,3
	tibia L	3,36	3,47	2,91	2,88	3,88	2,29
	basitarsus L	1,85	1,49	1,79	1,63	2,19	1,28
	telotarsus L	0,88	0,78	1,01	0,77	1,12	0,69
Leg IV	L^5^	13,86	0	14,41	12,75	15,85	11,07
	patela L	4,98	-	4,75	4,03	4,98	3,61
	tibia L	4,98	-	4,5	4,04	5,17	3,64
	basitarsus L	3,9	-	3,37	3,12	3,79	2,6
	telotarsus L^6^	-	-	1,79	1,56	1,91	1,22

L = length; W = width; H = height.

^1^ Measured along medial axis, from the propeltidium anterior margin to the opisthosoma posterior margin.

^2^ Measured in retrolateral view from chelicero-peltidial condyle to the tip of fixed finger mucron.

^3^ Measured in dorsal view at widest point.

^4^ Measured in retrolateral view at highest point of the manus.

^5^ Sum of individual segments length.

^6^ Measurement excludes claws.

**Table 6:** Variability on metric data for *Pseudocleobis mumai* n. sp., *P. cekalovici* n. sp. and *P. escuadra* n. sp. Values in millimeters.

		*P. mumai* n. sp.			*P. cekalovici* n. sp.						*P. escuadra* n. sp.					
		male (n = 3)			male (n = 10)			female (n = 5)			male (n = 6)			female (n = 6)		
		MIN	MEAN	MAX	MIN	MEAN	MAX	MIN	MEAN	MAX	MIN	MEAN	MAX	MIN	MEAN	MAX
TB	L^1^	6,30	7,51	8,41	5,98	7,82	9,02	8,66	9,94	11,29	7,79	8,99	9,67	8,04	9,80	12,48
Prop.	L	1,52	1,57	1,62	1,13	1,47	1,60	1,57	1,73	1,93	1,59	1,73	2,03	1,44	1,68	2,11
	W	1,72	1,85	1,95	1,59	1,94	2,11	2,42	2,66	2,74	1,61	2,15	2,43	2,25	2,70	3,31
Chel.	L^2^	2,44	2,55	2,75	2,20	2,53	2,86	3,28	3,66	4,02	2,61	3,01	3,33	2,95	3,69	4,21
	W^3^	0,71	0,75	0,81	0,61	0,80	0,89	0,97	1,15	1,34	0,84	0,92	0,97	0,90	1,20	1,46

TB = Total Body; Prop. = Propeltidium; Chel. = Chelicera; L = length; W = width; H = height.

^1^ Measured along medial axis, from the propeltidium anterior margin to the opisthosoma posterior margin.

^2^ Measured in retrolateral view from chelicero-peltidial condyle to the tip of fixed finger mucron.

^3^ Measured in dorsal view at widest point.

*Pseudocleobis andinus* (Pocock): Muma, 1971 [9]: 15–16, Fig 25 (misidentification in part).

**Type material.** Holotype: CHILE: Coquimbo region: ♂, Elqui province, Las Hedionditas, camino a embalse La Laguna, I.1966, L.E. Peña coll., (MCZ 170348). Paratypes: CHILE: Coquimbo region: 1♂, 1 juv, same data as holotype, (MCZ 170349); 1♂, 1♀, Elqui province, El Indio gold Mine C.M.I., II-1993, 4250 m. a.s.l. (MNNC 8427–8428).

**Etymology.** The specific epithet is given in honor of Dr. Martin H. Muma (1916–1989), American arachnologist who made important contributions to Chilean solifuge fauna.

**Diagnosis.** Differs from *P. elongatus* n. sp., *P. atacamensis* n. sp. and *P. puna* n. sp. by the shorter prodorsal flange, and a wider flagellar groove (Fig 29B and 29C). Differs from *P. krausi* n. sp., *P. choros* n. sp. and *P. lalackama* n. sp., by the lack of retroventral flange (Figs 28D and 29E) and the flagellum, that is proportionally smaller. Resembles *P. cekalovici* n. sp. and *P. escuadra* n. sp. in the morphology of male fixed finger mucron. Differs from the first by prodorsal flange which is more curved downwards (Figs 28B, 28D, 29B and 29E) and by the proventral flange which is not curved upward (Fig 29B). Differs from *P. escuadra* n. sp. by the less elevated and less pronounced prodorsal flange (Figs 28B, 28D, 29B, 29C and 29E).

**Note:** The known specimens of this species are in poor conditions. The female seems to have been dried, and missing legs. The male holotype lacks both telotarsus IV.

**Description.** Measurements in Table 5. Male holotype: *Chelicerae*: Dentition and processes: fixed finger: median series with FD, 1 FSD (vestigial in left chelicera), FM, 1 FSM and FP teeth (Fig 29A); FSD tooth tiny, fused with FD tooth (Fig 29A and 29B); FP tooth similar in size as FM tooth; retrofondal series with RFM, 2 RFSM (the posterior is tiny; see variability), RFP, 1 RFSP teeth (Fig 29A, 29D and 29E) and profondal series with PFM, 1 PFSM, PFP, 1 PFSP teeth (Figs 28C, 28D, 29D and 29E). Fixed finger mucron long, proximally wide, anteriorly narrow. Flagellar groove with prodorsal flange shortly extending beyond FD tooth (Fig 29B and 29C), and strongly curved downwards (Figs 28D, 29B and 29E); proventral flange almost straight (Fig 29B). Movable finger: with MM, 1 MSM and MP teeth (Fig 29A); MM bigger than FP; MP is the biggest tooth of the chelicera; movable finger mucron approx. 1.5 times as long as fixed finger mucron (Figs 28A, 28B and 29A), slightly curved; gnathal edge carina poorly elevated and not forming a convex bulge; tip distinctly curved upward (Figs 28A, 28B and 29A). Stridulatory plate with 8 parallel ridges. *Flagellum*: flagellum base teardrop-shaped (Figs 28B and 29B), narrowing distally; dorsal margin of the base is convex to distal, whilst the ventral margin is almost straight. The stalk (attachment ring) situated on the distal end of the prolateral setose area, longitudinally between FSM and RFM teeth (Fig 29B). *Pedipalps*: Femur with a proventral row of five long lateroventral spiniform setae, and a imperfectly aligned retroventral row of four lateroventral spiniform setae; the retroventral smaller than the proventral and imperfectly paired with these. Tibia with 2.2.2.2.2 long ventral spiniform setae, proximal pair setiform, retroventral row shorter and more similar in size. Basitarsus with 2.2.2.2 long ventral spiniform setae, proximal pair setiform, distal pair shorter than the rest. *Opisthosoma*. Ctenidia present as thick setae, 3–3 on sternite IV (see variability).

**Female.** Morphology and chaetotaxy like males, except by the wider propeltidium, unmodified chelicerae (Fig 28E), and shorter legs and pedipalps. The spiniform setae of pedipalps are more spiniform than in males. Opisthosomal sternite III and IV without ctenidia. Genital plate (Fig 28F). bell-shaped, barely wider than long, with lateral margins slightly concave at distal half, and convex at proximal half; has a long middle cuticular open with margins convergent to distal (as an inverted long V); the two halves don’t touch each other posteriorly.

**Variability.** The male paratype (MCZ 170349) with 5–5 ctenidia on sternite III and 6–6 on sternite IV. The male paratype (MNNC 8427) with the FSD tooth vestigial on both chelicerae, and with one additional tiny posterior RFSM tooth on left chelicera. Size variability in Table 6.

**Distribution and ecology.** *Pseudocleobis mumai* n. sp. has been collected in the Andean sector of the Coquimbo Region, (Chile), between 3600 to 4300 m a.s.l. All localities are located within the high Andean steppe of the Doña Ana botanical subregion of the Andean desert botanical region [17, 43, 44].

Collections sites are associated with Andean wetland ecosystems called "vega", "humedal" or "bofedales" [43, 45]. These environments are characterized by an accumulation of water that results in permanently flooded soil. The temperature fluctuates between -20°C (winter), to more than 25°C (summer), although temperatures below zero can be recorded throughout the year, as well as precipitation in the form of snow (which can accumulate up to 8 m in some areas), these occur mainly during the winter [46]; The vegetation of the area corresponds to the high Andean steppe [45].

The specimens from El Indio were collected under stones associated with the periphery of the wetlands.

**Fig 30:**
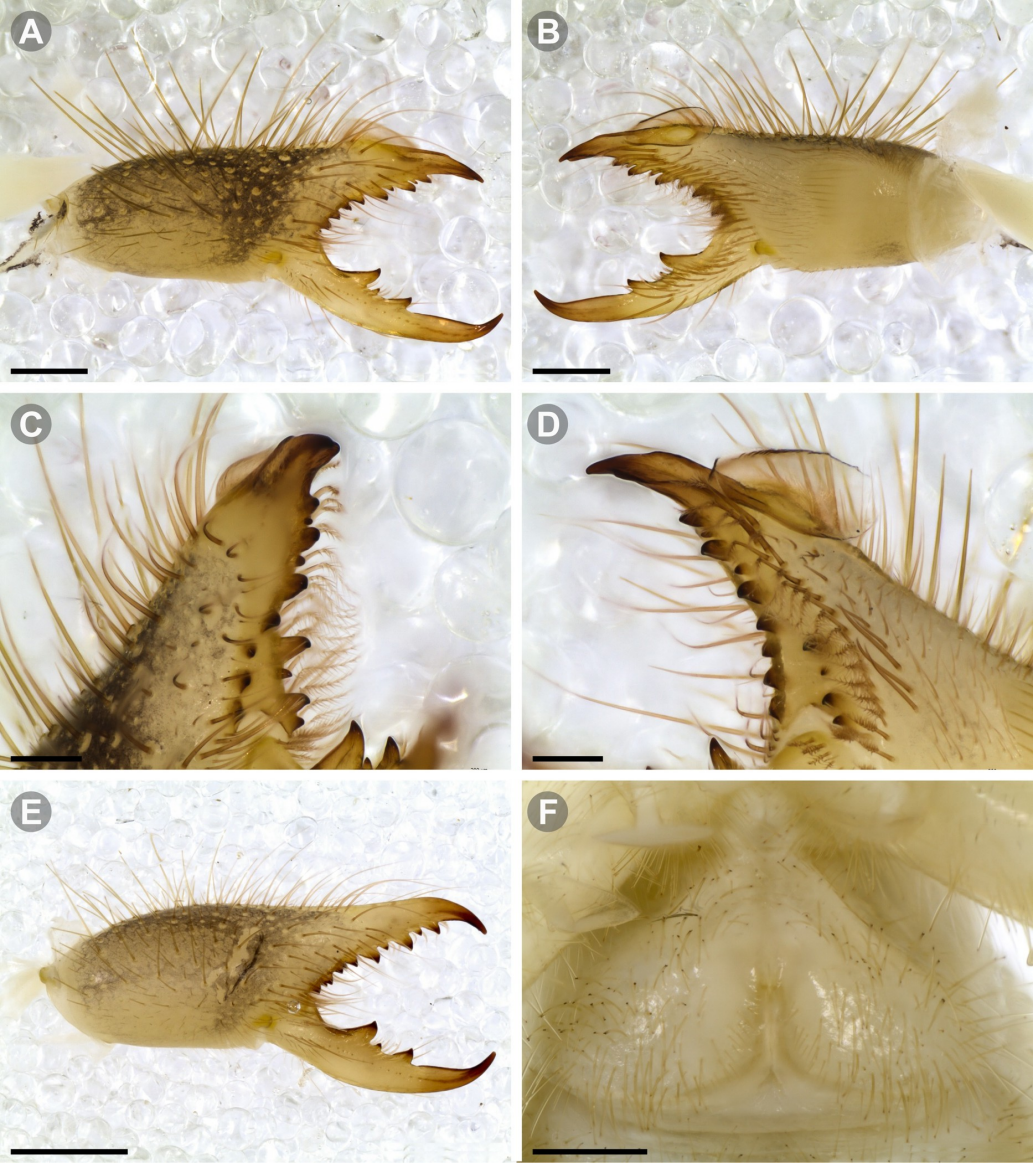
*Pseudocleobis cekalovici* n. sp. (A–E) Male holotype, right chelicera. (A) Retrolateral view. (B) Prolateral view. (C) Cheliceral fingers, retroventral view. (D) Cheliceral fingers, proventral view. (E) Fixed finger, dorsal view. (F) Male from Farellones, above Santiago (MCZ), right chelicera, retrolateral view. Scale bars: 0,5 mm (A–B, F); 0,2 mm (C–E).

**Fig 31:**
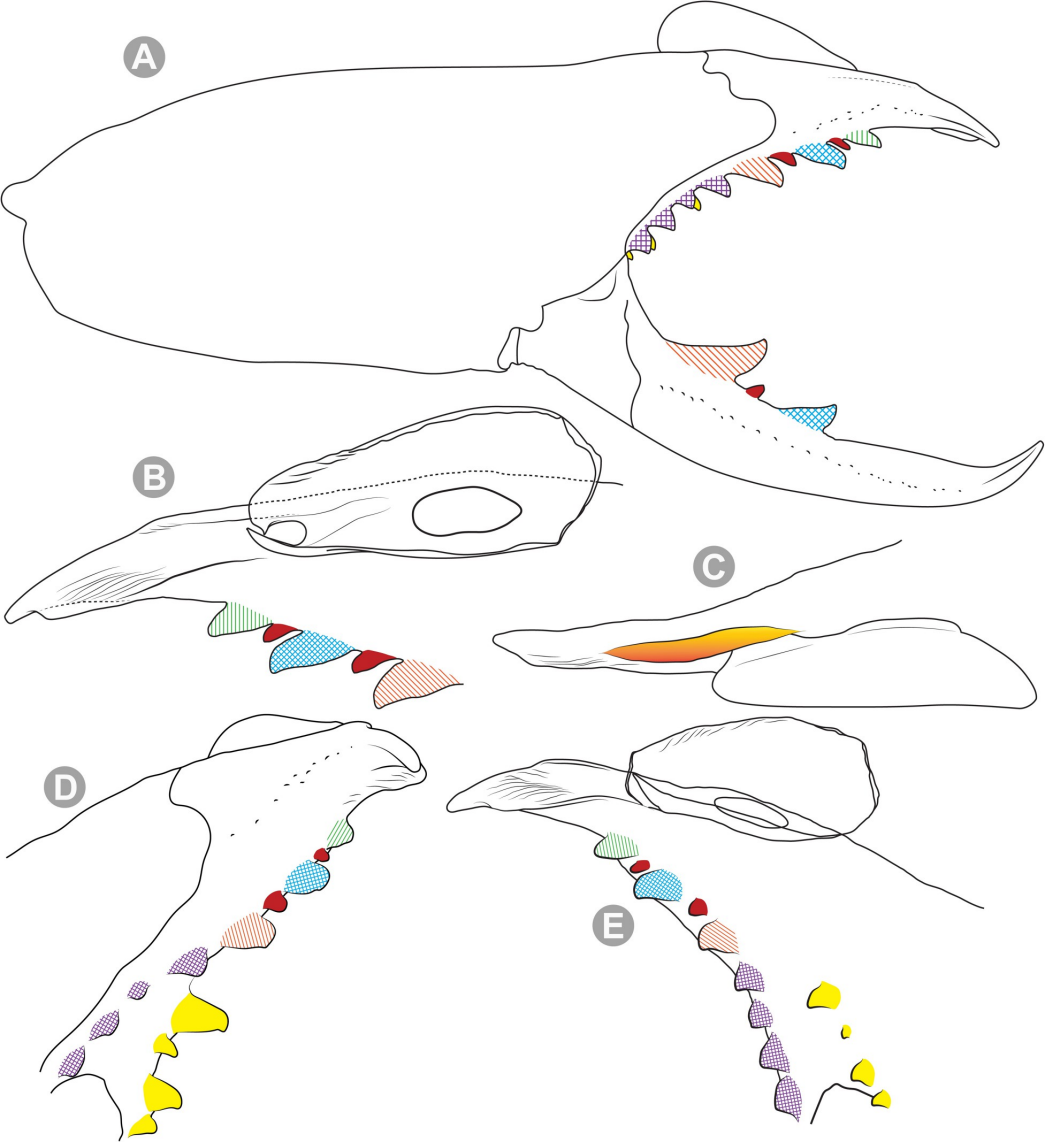
*Pseudocleobis cekalovici* n. sp., schemes of male right chelicera. (A) Retrolateral view. (A–E) Fixed finger. (B) Prolateral view. (C) Dorsal view (prodorsal flange orange colored). (D) Retroventral view. (E) Proventral view.

**Fig 32:**
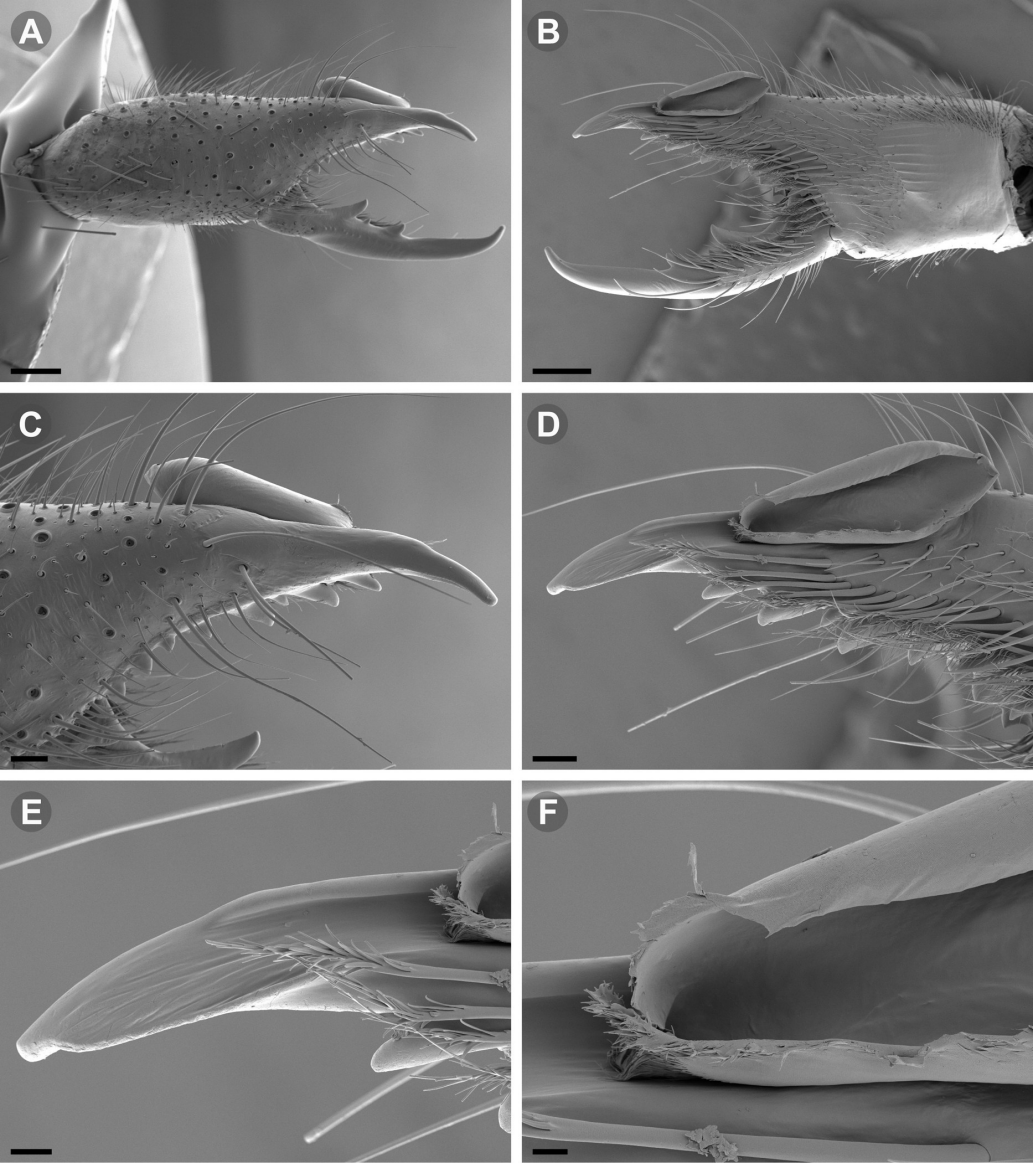
*Pseudocleobis cekalovici* n. sp., SEM images of male right chelicera. (A) Retrolateral view. (B) Prolateral view. (C) Cheliceral fixed finger, retrolateral view. (D) Cheliceral fixed finger, prolateral view. (E) Fixed finger mucron detail, proventral view. (D) Flagellum prolateral view, detail of distal area. Scale bars: 0,3 mm (A–B), 0,1 mm (C–E), 0,04 mm (E), 0,02 mm (D).

***Pseudocleobis cekalovici* Iuri & Maury n. sp.** urn:lsid:zoobank.org:act:AECABBD6-4B2B-429F-9DD4-4EEDAB827121.

(Figs 3B–3D, 5 and 30–32 and Tables 5 and 6).

*Pseudocleobis andinus* (Pocock): Muma, 1971 [9]: 15–16, Fig 26 (misidentification, in part).

**Type material.** Holotype: CHILE: Metropolitan region: ♂, Cordillera province, San José de Maipo commune, near rivera Río Volcán, 33°48’58.5’’S 70°00’00.8’’W, 2349 m. a.s.l., 16-XII-2023, manual collection at night, H.A. Iuri, A.A. Ojanguren-Affilastro, J. Pizarro-Araya, F.M. Alfaro, J.E. Calderón coll., (MNNC 8429). Paratypes: CHILE: Metropolitan region: 9♂, 1♀, 1juv, same data as holotype (MNNC 8430–8439); 5♂, same data as holotype (MACN-Ar 46526–46530); 5♂, same data as holotype (MZUC 48064–48068); 5♂, same data as holotype (LEULS 191–195); 4♂, 3♀, same locality and collectors as holotype, 16-19-XII-2023, pitfall trap (LEULS 196–202); 2♂, Cordillera province, San José de Maipo commune, Cascada Queltehua, 33°49’53.0’’S 70°07’00.3’’W, 1712 m. a.s.l., 16-XII-2023, manual collection at night, H.A. Iuri, A.A. Ojanguren-Affilastro, J. Pizarro-Araya, F.M. Alfaro, J.E. Calderón coll., (MACN-Ar 46531–46532).

**Other material.** CHILE: Valparaiso region: 1♂, Los Andes province, El Juncal, 27-X-1988, E.A. Maury coll., (MACN-Ar 46542); 1♂, Los Andes province, Río Blanco, 29-IX-1983, E.A. Maury coll., (MACN-Ar 46543). Metropolitan region: 6♂, 2♀, same data as holotype (MACN-Ar 46534–46541); 1♂, Cordillera province, San José de Maipo commune, Cascada Queltehua, 33°49’53.0’’S 70°07’00.3’’W, 1712 m. a.s.l., 16-XII-2023, manual collection at night, H.A. Iuri, A.A. Ojanguren-Affilastro, J. Pizarro-Araya, F.M. Alfaro, J.E. Calderón coll., (MACN-Ar 46533); 1♂, Santiago province, Farellones, above Santiago, 28-XI-1962, P.J. Darlington (MCZ 170350; Muma [9] mentioned this specimen as female).

**Etymology.** The specific epithet is given in honor of Tomas Cekalovic Kuschevich (1928–2013), Chilean arachnologist and entomologist who contributed to the knowledge of Chilean solifuges.

**Diagnosis.** Differs from *P. elongatus* n. sp., *P. atacamensis* n. sp. and *P. puna* n. sp. by the shorter prodorsal flange (Figs 30B, 31B and 32D), and the wider flagellar groove (Figs 31B and 32C). Differs from *P. krausi* n. sp., *P. choros* n. sp.and *P. lalackama* n. sp., by the lack of retroventral flange (Figs 30D, 31E and 32C) and by the flagellum, which is proportionally smaller. Resembles *P. mumai* n. sp. and *P. escuadra* n. sp. in the morphology of male fixed finger mucron. Differs from the first by the prodorsal flange which is not or barely curved downwards (Figs 31B, 31C and 32E) and by the proventral flange which runs downwards and is curved downwards distally (Figs 31B and 32E). Differs from *P. escuadra* n. sp. by the less pronounced and less elevated prodorsal flange (Figs 30A, 31A–31C, 32D and 32E).

**Description.** Measurements in Table 5. Male holotype: *Chelicerae*: Dentition and processes: fixed finger: median series with FD, 1 FSD, FM, 1 FSM and FP teeth (Fig 31A); FSD tooth small, not fused at the base with FD and/or FM tooth; FP tooth similar in size as FM tooth; retrofondal series with RFM, 1 RFSM, RFP, 1 RFSP teeth (Fig 31A and 31D) and profondal series with PFM, 1 PFSM, PFP, 1 PFSP teeth (Figs 30C and 31D). Fixed finger mucron long and wide; flagellar groove wide, with prodorsal flange not or barely curved downwards, andreaching almost the half mucron length (Figs 31B, 31C, 32D and 32E); proventral flange runs downwards and is curved downwards distally (Figs 31B, 32D and 32E). Movable finger: with MM, 1 MSM and MP teeth (Fig 31A); MM slightly bigger than FP; MP is the biggest tooth of the chelicera; movable finger mucron approx. 1.5 times as long as fixed finger mucron, slightly curved; gnathal edge carina poorly elevated, forming a slightly convex bulge proximally; mucron tip distinctly curved upward (Figs 30A, 30B, 31A and 32A). Stridulatory plate with 9 parallel ridges. *Flagellum*: flagellum base slightly pear-shaped (Figs 30A and 31B), narrowed distally; dorsal and ventral margins run slightly concave to distal. It has a lateral folding at the apex (Figs 31C and 32F). The stalk (attachment ring) situated on the distal end of the prolateral setose area, longitudinally between FSM and RFM teeth (Fig 31B). *Pedipalps*: Femur with a proventral row of five long lateroventral spiniform setae, and a imperfectly aligned retroventral row of four lateroventral spiniform setae; the retroventral smaller than the proventral and imperfectly paired with these. Tibia with 2.2.2.2.2 long ventral spiniform setae, proximal pair weaker, retroventral row smaller and more similar in size; there is a small additional spiniform retroventral seta between the medial and the subdistal. Basitarsus with 2.2.2.2 spiniform setae, proximal (basal) pair weak, replaced by a pair of thick bifurcated tip setae, distal pair smaller. *Opisthosoma*: Ctenidia present but hard to distinguish, 3–3 on sternite III and 3–3 on sternite IV.

**Female.** Morphology and chaetotaxy like males, except by the wider propeltidium, unmodified chelicerae (Fig 30E), and shorter legs and pedipalps (Fig 3D). The spiniform setae of pedipalps are more spiniform than in males. Opisthosomal sternite III and IV without ctenidia. Genital plate (Fig 30F). bell-shaped, barely wider than long, with lateral margins slightly concave at distal half, and convex at proximal half; has a long middle cuticular open, with margins convergent at proximal, but almost parallel at distal, till the half of the plate (Fig 30F); the two halves don’t touch each other posteriorly.

**Variability.** This is the only species in which we were able to examine a relatively large number of specimens, 41, of which 34 are from the same locality and collection event. Among the males of the type locality, one lacks the FSD tooth in right chelicera, one has a vestigial FSD tooth in left chelicera, one has a vestigial FSD tooth in right chelicera, and two have a tiny additional anterior RFSM tooth in left chelicera; the accessory spiniform seta of pedipalpal tibia is reduced in one of the pedipalps in seven males (5 on right, 2 on left), and one male has it reduced on both pedipalps; one male has an additional spiniform seta between the medial and the subbasal retrolateral spiniform setae of left tibia; the basal pair of of spiniform setae of the basitarsus are poorly developed, and replaced by a bifurcated tip setae, but in some specimens the prolateral might be thicker and with more prominent socket, being more easy to recognize. The male paratypes from Valparaiso region (MACN-Ar 46542–46543) are smaller in size and have the fixed finger mucron slightly longer. The male (MACN-Ar 46542) lacks the FSD in the left chelicera. The male from Farellones (MCZ 170350; S4 Fig) although the flagellar furrow and the pro and retroventral flanges are like the others specimens of this species, present slightly shorter mucra and with a blunt tip; however, this seems to be the result of the wear of the tips. Size variability in Table 6.

**Note:** The male specimen (MCZ 170350; S4 Fig), found and examined at the MACN, has an identification label by MH Muma describing it as “*Pseudocleobis andinus*”, which is consistent with his paper [21].

**Distribution and ecology.** This species has been collected in intermediate altitudes in the Andes neighboring Santiago city, in Metropolitan region. This area belongs to the Andean Mediterranean low scrub of *Chuquiraga oppositifolia* D. Don. and *Nardophyllum lanatum* (Meyen) [40]. The records are from intermediate altitudes between 1600 and 2400 m a.s.l in localities belonging to the Mediterranean Andes botanical subregion, in the High Andean Steppe botanical region [17]. Most of the specimens from the type locality were collected using headlights during a very windy night, with low temperatures. Despite the harsh weather all specimens were active.

**Fig 33:**
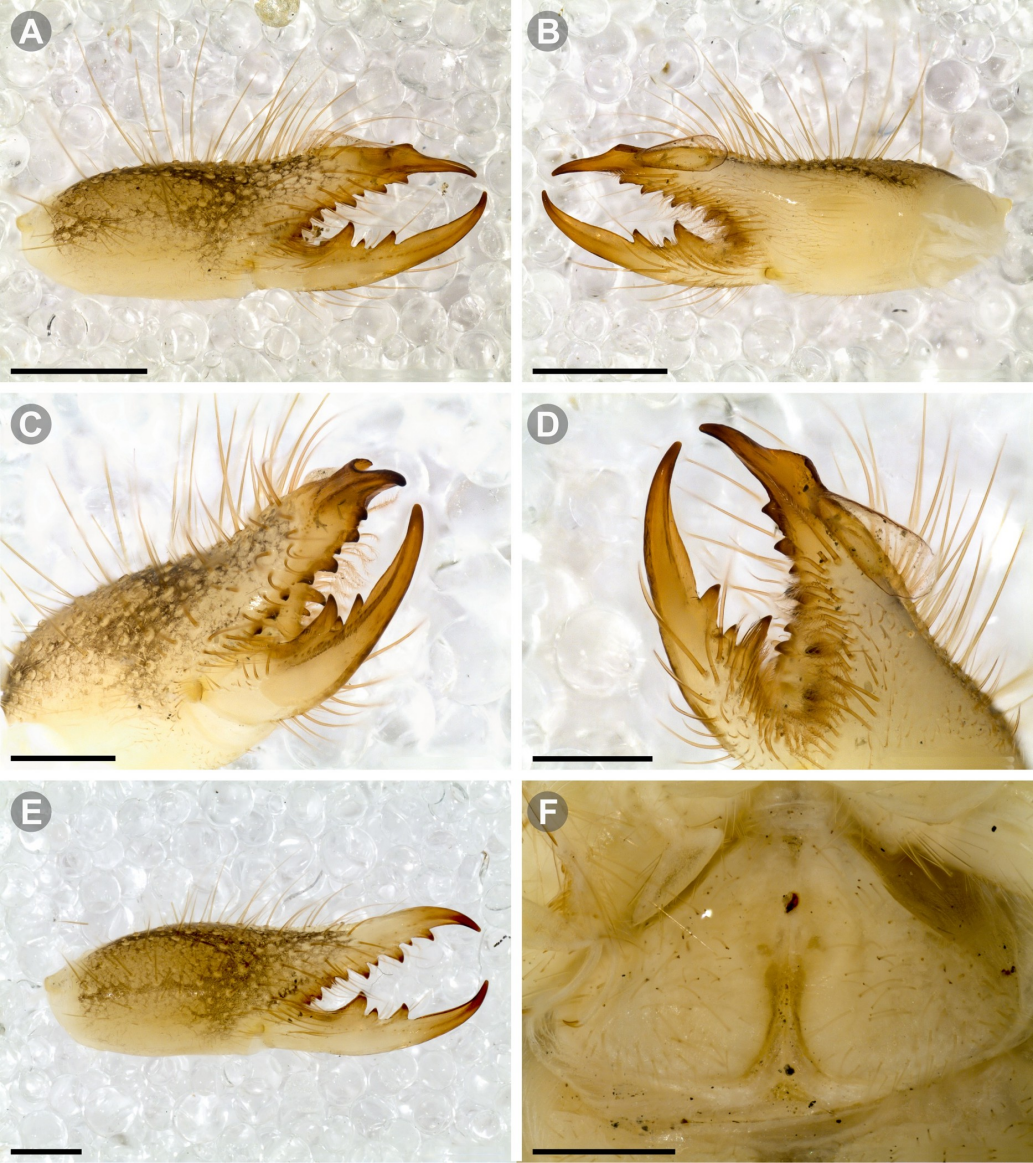
*Pseudocleobis escuadra* n. sp. (A–D) Male holotype, right chelicera. (A) Retrolateral view. (B) Prolateral view. (C) Retroventral view. (D) Proventral view. (E–F). Female paratype. (E) Right chelicera, retrolateral view. (F) Genital sternite, ventral view. Scale bars: 1 mm (A–B); 0,5 mm (C–F).

**Fig 34:**
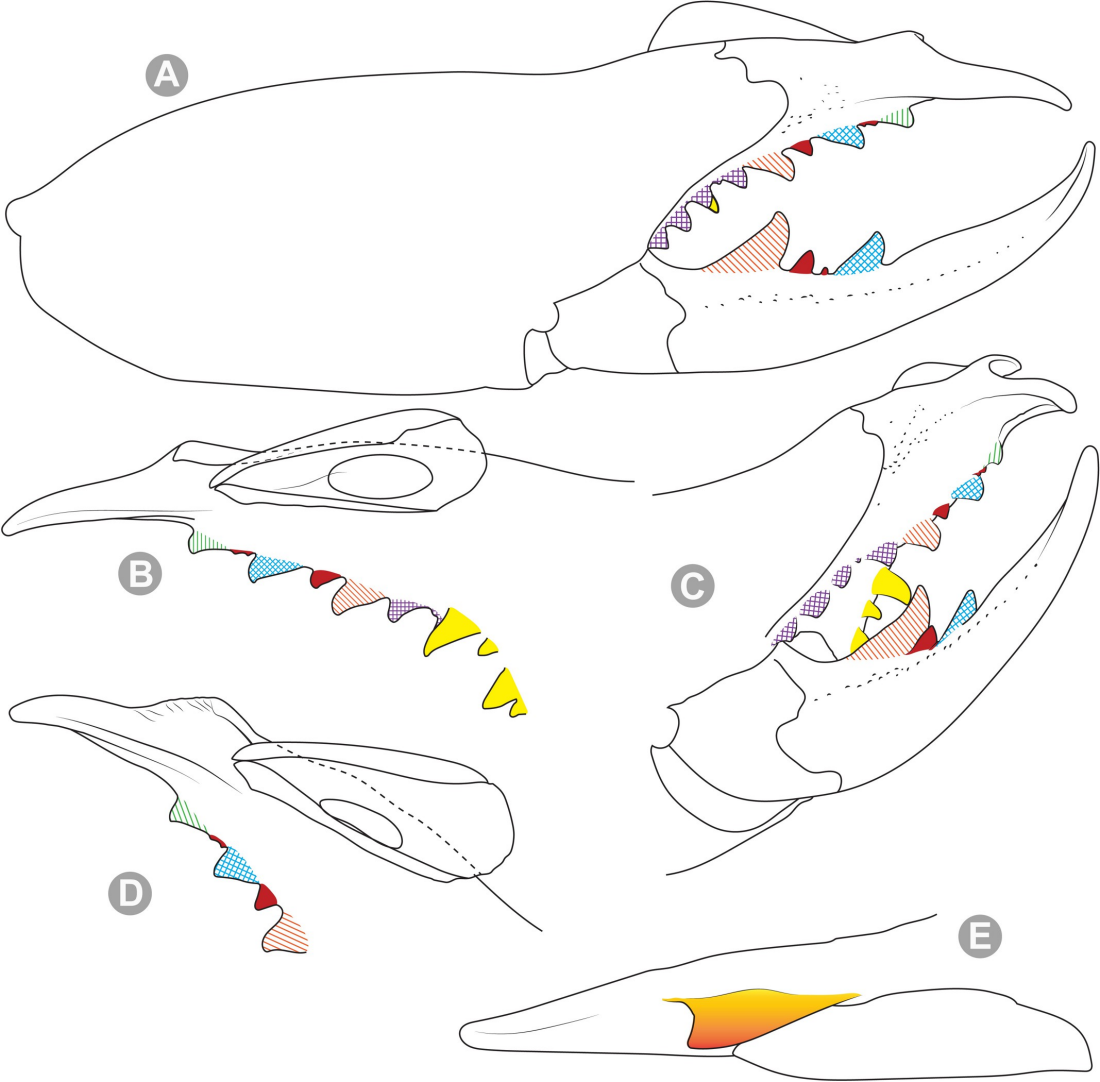
*Pseudocleobis escuadra* n. sp., schemes of male right chelicera. (A) Retrolateral view. (A–E) Fixed finger. (B) Prolateral view. (C) Retroventral view. (D) Proventral view. (E) Dorsal view (prodorsal flange orange coloured).

**Fig 35:**
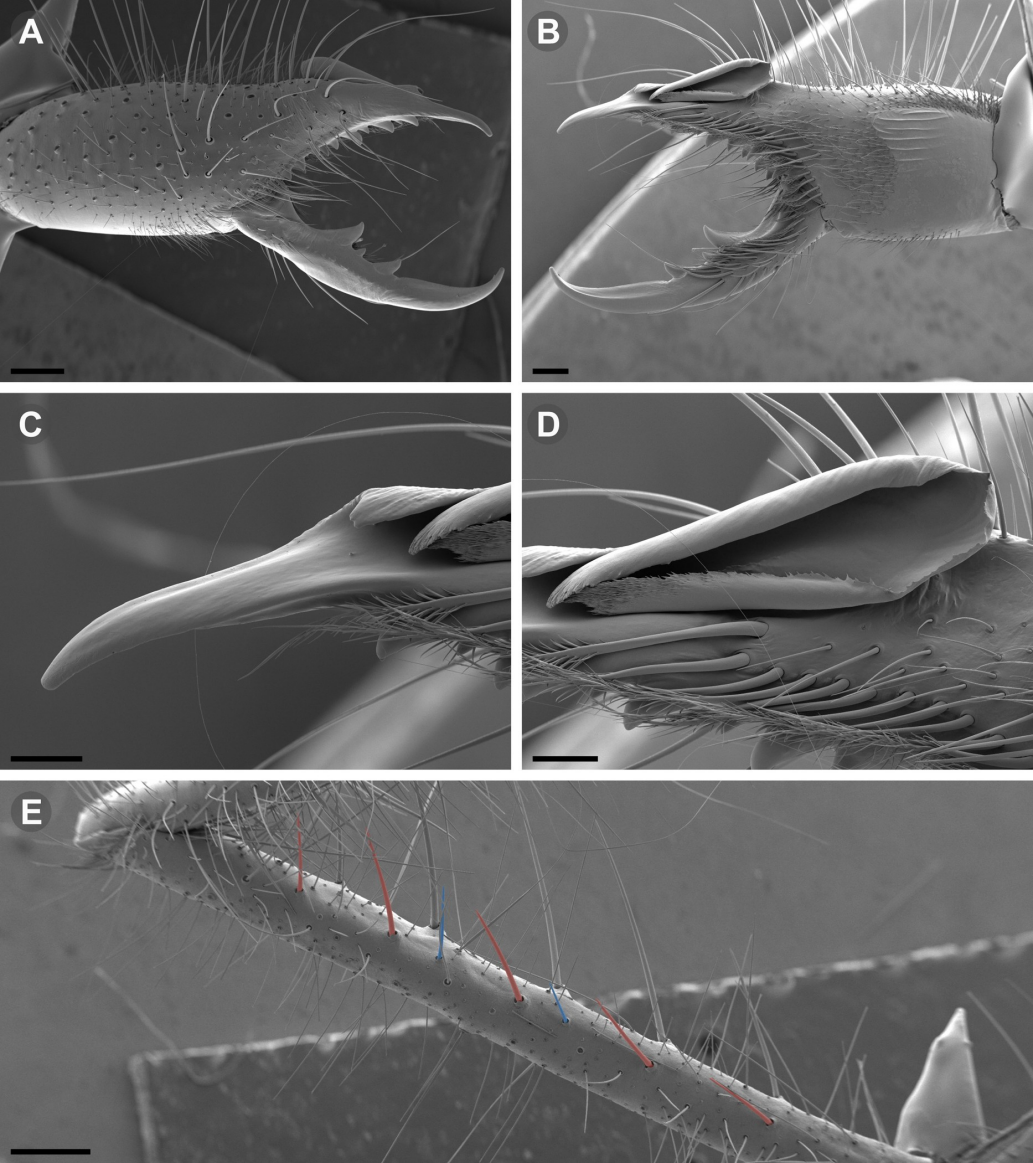
*Pseudocleobis escuadra* n. sp., SEM images of male right chelicera. (A) Retrolateral view. (B) Prolateral view. (C) Mucron detail, retrolateral view. (D) Flagellum detail, prolateral view. (E) Tibia of right pedipalp, retroventral view, retroventral spiniform seta in red (main) and blue (supplementary). Scale bars: 0,4 mm (E), 0,3 mm (A), 0,2 mm (B), 0,1 mm (C–D).

***Pseudocleobis escuadra* Iuri n. sp.** urn:lsid:zoobank.org:act:EA186211-5C16-4849-9C57-A68DD53685E7.

(Figs 3A, 5, 33–35 and Tables 5 and 6).

**Type material.** Holotype: CHILE: Maule region: ♂, Talca province, San Clemente commune, Fundo La Escuadra, sitio 4, 35°44’21.8’’S 70°46’43.7’‘W, 1292 m a.s.l.,16-X-2022, J. Pizarro-Araya, F.M. Alfaro and J.E. Calderón coll., (MNNC 8440). Paratypes: CHILE: Maule region: 1♀, same data as holotype (MNNC 8441); 1♂, 1♀, Fundo La Escuadra, sitio 2, 35°43’16.1’’S 70°47’04.9’‘W, 1260 m a.s.l., same date and collectors as holotype, (MACN-Ar 46544–46545). 2♂, Talca province, San Clemente commune, Fundo La Escuadra, sitio 2, 35°45’14.0’’S 70°47’09.9’‘W, 1135 m a.s.l.,9-XII-2023, pitfall trap (S2R3), J. Pizarro-Araya, F.M. Alfaro and J.E. Calderón coll., (MNNC 8442–8443); 2♀, same but pitfall trap (S2R1), (MNNC 8444–8445); 1♂, 1♀, Talca province, San Clemente commune, Fundo La Escuadra, sitio 4, 34°44’42.1’’S 70°46’59.4’‘W, 1258 m a.s.l.,9-XII-2023, pitfall trap (S4R1), J. Pizarro-Araya, F.M. Alfaro and J.E. Calderón coll., (LEULS 203–204); 1♀, same but pitfall trap (S4R2), (LEULS 205); 2♂, Talca province, San Clemente commune, Fundo La Escuadra, sitio 2, 35°45’14.0’’S 70°47’09.9’‘W, 1135 m a.s.l.,9-XII-2023, J. Pizarro-Araya, F.M. Alfaro and J.E. Calderón coll., (MACN-Ar 46546–46547);

**Etymology.** The specific epithet refers to the type locality Fundo La Escuadra, in the Maule region.

**Diagnosis.** Differs from *P. elongatus* n. sp., *P. atacamensis* n. sp. and *P. puna* n. sp. by the shorter prodorsal flange, and the wider flagellar groove (Figs 33B–33D, 34B–34E and 35B–35D). Differs from *P. krausi* n. sp., *P. choros* n. sp. and *P. lalackama* n. sp., by the lack of retroventral flange (Figs 33D, 34A, 34C, 34D and 35A) and by the flagellum which is proportionally smaller. Resembles *P. mumai* n. sp. and *P. cekalovici* n. sp. by the morphology of male fixed finger mucron and the flagellum. Differs from both by the more prominent and elevated prodorsal flange (Figs 33B–33D, 34B–34E, 35B and 35C).

**Description.** Measurements in Table 5. Male holotype: *Chelicerae*: Dentition and processes: fixed finger: median series with FD, 1 FSD (two vestigial in right chelicera), FM, 1 FSM and FP teeth (Fig 34A); FSD tooth small, not fused at the base with FD and/or FM tooth; FP tooth similar in size to FM tooth; retrofondal series with RFM, 1 RFSM, RFP, 1 RFSP teeth (Fig 34A and 34C) and profondal series with PFM, 1 PFSM, PFP, 1 PFSP teeth (Fig 34B). Fixed finger mucron long, narrowing distally (Fig 34A); flagellar groove proximally wide, with prominent prodorsal flange, strongly curved downwards, extending shortly beyond the FD tooth, and proventral flange not curved (Figs 33A–33D and 34). Movable finger: with MM, 1 MSM and MP teeth (Fig 34A); MM bigger than FP; MP is the biggest tooth of the chelicera; movable finger mucron (Figs 33A, 33B and 34A) approx. 1.5 times as long as fixed finger mucron, slightly curved and the gnathal edge carina forms a convex bulge at proximal half (Figs 33A and 34A). Stridulatory plate with 8 parallel ridges. *Flagellum*: flagellum base (Fig 34A) proportionally small, teardrop-shaped, narrowing distally, not extending beyond the FD tooth; dorsal and ventral margin run almost straight to distal. The stalk (attachment ring) situated on the distal end of the prolateral setose area, longitudinally between FSM and RFM teeth (Fig 34B). *Pedipalps*: Femur with a proventral row of five long lateroventral spiniform setae, and a imperfectly aligned retroventral row of four lateroventral spiniform setae; the retroventral smaller than the proventral and imperfectly paired with these. Tibia with 2.2.2.2.2 long ventral spiniform setae, retroventral row smaller and more similar in size; there is a small additional spiniform retroventral seta between subbasal and medial, and another one between medial and subdistal (Figs 8A and 35E). Basitarsus with 2.2.2.2 long ventral spiniform setae, proximal pair weaker, distal pair smaller. *Opisthosoma*: Ctenidia present as thick reddish setae, 6–6 on sternite III and 5–5 on sternite IV.

**Female.** Morphology and chaetotaxy like males, except by the wider propeltidium, unmodified chelicerae (Fig 33E), and shorter legs and pedipalps. The spiniform setae of pedipalps are more spiniform than in males; there is an additional small retroventral spiniform seta on basitarsus, between the subbasal and subdistal (Fig 8A). Opisthosomal sternite III and IV without ctenidia. Genital plate (Fig 33F). bell-shaped, wider than long, with lateral margins concave at distal half, and slightly convex at proximal half; with a posterior cuticular opening, with margins convergent at proximal, but almost parallel at distal, till the half of the plate (Fig 33F), and a distal middle oval circular depression; the two halves don’t touch each other posteriorly.

**Variability.** Two male paratypes (MACN-Ar 46544 and LEULS 203) with 6–6 ctenidia on sternite III and 6–6 on sternite IV. One male paratype (MACN-Ar 46546) with 4–4 ctenidia on sternite III and 4–4 on sternite IV. One male paratype (MNNC 8442) with 5–**6** ctenidia on sternite III and 6–5 on sternite IV. One male paratype (M**N**NC 8443) with 5–5 ctenidia on sternite III and 4–3 on sternite IV. One male paratype (LEULS 203) has a very tiny tooth (RFA?) posterior to FP in right chelicera. The male paratype (MNNC 8442) has an additional tiny posterior RFSM tooth in left chelicera. The Female paratype (MNNC 8444) has an additional tiny anterior RFSM tooth in left chelicera. The female paratype (LEULS 204) has the left chelicera with one additional posterior FSD, one additional anterior RFSM, and an additional anterior MSM teeth; the right chelicera with one additional posterior FSD, and two additional (one anterior and one posterior) RFSM teeth. Size variability in Table 6.

**Distribution and ecology.** This species is only known from the type locality, between the Hornitos and Quizapú volcanoes, near the Maule River, placed at intermediate altitudes of the Andes in the Maule region, in south-central Chile. The botanical formation corresponds to the mountain deciduous forest and high Andean Maule steppe [17].

This species has been collected only in the northern limit of Fundo La Escuadra, a highly preserved private natural area in Maule region. The specimens were found at night, associated with rock edges, on volcanic sand substrates near the Invernada lagoon and in cypress forests of the *Austrocedrus chilensis* (D. Don) Pic-Serm. & Bizzarri. This area (Fundo La Escuadra) is considered the southernmost limit of the high Andean steppes. Towards the south there is a change in ecological conditions, related to an increase in rainfall and snowfall in the area, marking a natural limit for the distribution of many southern and boreal species [17]. The environment of this zone is heterogeneous, covered by dry forests, shrubby steppes (e.g., *Chuquiraga oppositifolia* D. Don, *Gochnatia fololiosa* D. Don and *Proustia cuneifolia* D. Don (Asteraceae), *Discaria articulata* (Phil.) Miers (Rhamnaceae), *Fabiana imbricata* Ruiz & Pav. (Solanaceae)), and steppe grasses (eg, *Acaena alpina* Poepp. ex Walp. (Rosaceae) and *Festuca acanthophylla* Desv. (Poaceae)).

### Key to males of Chilean species of *Pseudocleobis*

**1A.** Prodorsal flange of flagellar groove long and straight, almost reaching the mucron tip (Figs 12B, 14B, 17B and 20B).

……………………………………………………………………………………………………**2**

**1B.** Prodorsal flange of flagellar groove short, not reaching further the half of the mucron (Figs 22B, 24B, 26B, 29B, 31B and 34B).

……………………………………………………………………………………………………**4**

**2A.** Proventral flange of flagellar groove curved upward proximally (Fig 11F).

……………………………………………………………***Pseudocleobis alticola* Pocock, 1900**

**2B.** Proventral flange of flagellar groove not curved upward (Figs 15D, 18D and 20B).

……………………………………………………………………………………………………**3**

**3A.** Small retroventral flange present (Fig 15E). Proventral flange expanded downwards distally (Fig 15D).

……………………………………………………………………***Pseudocleobis elongatus* n. sp.**

**3B.** Retroventral flange absent. Proventral flange not expanded downwards distally.

……………………………………………………………………………………………………**4**

**4A.** Flagellar groove narrowing at distal third (proventral flange runs upward at distal third; Figs 17B and 18D).

…………………………………………………………………***Pseudocleobis atacamensis* n. sp.**

**4B.** Flagellar groove with the same width throughoutits length (proventral flange runs upward at the tip of mucron; Fig 20B).

………………………………………………………………………***Pseudocleobis puna* n. sp.**

**5A.** Retroventral flange present (Figs 22D, 24E and 26E). Flagellum greatly expanded at dorsal.

……………………………………………………………………………………………………**6**

**5B.** Retroventral flange absent. Flagellum poorly expanded at dorsal.

……………………………………………………………………………………………………**8**

**6A.** Retroventral flange short, not expanded downwards (Fig 22A, 22D and 22E).

………………………………………………………………………***Pseudocleobis krausi* n. sp.**

**6B.** Retroventral flange evident, expanded downwards, hiding part of the gnathal edge carina in lateral view (Figs 24A, 26A and 27C).

……………………………………………………………………………………………………**7**

**7A.** Prodorsal flange extends beyond the FD tooth almost to the half of the mucron (Fig 24A–24C). Retroventral flange greatly expanded (Fig 24E).

………………………………………………………………………***Pseudocleobis choros* n. sp.**

**7B.** Prodorsal flange barely extends beyond the FD (Figs 26A–26C and 27D). Retroventral flange not greatly expanded (Fig 26E).

…………………………………………………………………‥***Pseudocleobis lalackama* n. sp.**

**8A.** Proventral flange strongly curved downwards distally (Fig 32E). Prodorsal flange not or barely curved downwards (Fig 32E).

…………………………………………………………………‥***Pseudocleobis cekalovici* n. sp.**

**8B.** Proventral flange not or barely curved downward distally. Prodorsal flange evidently curved downwards (Figs 29B and 35C).

……………………………………………………………………………………………………**9**

**9A.** Prodorsal flange prominent and abruptly higher than the distal part of the mucron (Figs 34A, 34B and 35C).

………………………………………………………………………***Pseudocleobis escuadra* n. sp.**

**9B.** Prodorsal flange not prominent and not abruptly higher (Figs 28A, 28B and 29B).

………………………………………………………………………***Pseudocleobis mumai* n. sp.**

## Discussion

*Pseudocleobis* is the most diversified ammotrechid genus from South America with currently 29 species (including the species described herein).

The characters used by Roewer [7] to define the species of the genus, i.e., the number of secondary teeth and spiniform setal pattern of pedipalp, proved to have some degree of intraspecific variability and thus are not useful to diagnose at the species level, as has already been reported in previous works regarding species of this genus [2, 9, 10]. The patterns and variations herein described for the spiniform setae of the pedipalps explain most of the patterns reported in old descriptions, therefore rendering their diagnoses doubtful. However, this trait needs to be studied in depth and on a large series of specimens, as some patterns might be useful as supplementary diagnostic character at the species group level.

The *Pseudocleobis* species from Chile can be divided, regarding its cheliceral morphology, into two groups. The first one includes *P. elongatus* n. sp., *P. atacamensis* n. sp., and the high-Andean *P. puna* n. sp., characterized by narrow flagellar furrow and long prodorsal flange. The male cheliceral morphology of this group has some resemblance with the chelicera morphology of the high-Andean species *P. alticola* and might be closely related.

The second group includes the species, *P. krausi* n. sp., *P. choros* n. sp., *P. lalackama* n. sp., *P. mumai* n. sp., *P. cekalovici* n. sp., and *P. escuadra* n. sp., characterized by the flagellar furrow with short prodorsal flange. The presence of a small supplementary retrolateral spiniform seta on pedipalpal tibia seems to be frequent in this group. This group can be subdivided into two sub-groups, regarding the presence/absence of retroventral flange, which seems to be an expansion of the retrolateral edge carina. The species, *P. krausi* n. sp., *P. choros* n. sp., and *P. lalackama* n. sp., distributed from the coastal desert in north Coquimbo region to south Antofagasta region, have an evident, expanded, retroventral flange, while *P. mumai* n. sp., *P. cekalovici* n. sp., and *P. escuadra* n. **sp.**, distributed through the central Chilean Andes, lack this retroventral flange (or the edge carina is not expanded).

The retroventral flange has been reported so far only in the High-Andean species *P. andinus* (Fig 7A). The cheliceral morphology of this species has some resemblance with the Chilean coastal species with which it shares this trait, *P. choros* n. sp., *P. krausi* n. sp., and *P. lalackama* n. sp., that also share the presence of a distal middle area with pointed projections on the prolateral surface of the flagellum (Fig 7B), suggesting a close relationship between them.

The possible relation between the high-Andean species with different lineages of Chilean lowland species may suggest the existence of an Andean-Pacific clade. A similar evolutionary history was supported by molecular data for the scorpion genus *Brachistosternus* Pocock, 1893 [47, 48], which has a similar distribution pattern to the genus *Pseudocleobis*.

### New species and implications for the knowledge of diversity

The high number of species recorded in this study, increasing the number of described species of Chilean *Pseudocleobis* from 3 to 10, indicates the evident underestimation of Chilean solifuge richness. This current lack of knowledge is mostly due to the scarce or unpublished sampling carried out in coastal, pre-Andean, and Andean environments along Chile. Despite the enormous collection effort presented in this contribution, we believe that future sampling in other different areas and seasons than those herein presented could lead to the description of even more new taxonomic entities for this group from Chile. This underestimation of species richness is a clear sign of the Linnean and Wallacean shortfalls related to the global lack of knowledge of the real diversity and geographic distribution of terrestrial arthropods, which is particularly severe in Chile, [49]. This problem is exacerbated by the increasing deficit of specialists able to tackle this effort, especially for the Chilean solifuge fauna, which has not been formally studied since 1987 [50]. Unfortunately, this is not a local problem only, as the development of taxonomy in many developing countries face serious challenges due to the low number of specialists dedicated to identifying and classifying species [51].

### New species and identification of interesting areas for the conservation

Although it is common to use big, vulnerable, charismatic vertebrates as flagship species for conservation efforts, this is not always feasible as some environments may lack vertebrate species that meet those requisites. In this context the use of invertebrate species as flagship species can be a valuable strategy to promote new local areas of conservation. The diversity revealed in this study in a poorly known group of arachnids becomes important for this purpose. Despite there are many places yet to be explored, most of the species described here seem to be highly endemic from specific areas (e.g., *P. lalackama* n. sp., *P. escuadra* n. sp.) and they could be taken in consideration as flagship species when proposing new local areas of conservation.

In Chile other arachnids’ species have already played important roles in local conservation efforts [e.g., 52], and hopefully some of the species herein described from preserved areas, could also play this role in the future.

## Conclusion

This study significantly expands our understanding of Chilean *Pseudocleobis*, increasing the number of described species a 300 percent, and shedding new light on their diversity. Despite the existence of few published works, our knowledge of the Chilean solifuge fauna remains limited. Moreover, the examination of numerous unidentified females and juveniles in this study hints at a potentially greater diversity yet to be discovered.

## Supporting information

S1 Fig *Pseudocleobis atacamensis* n. sp. vectors from Maury unpublished drawings of male specimen SMF 17368 from Dulcinea, Chile. (PDF)

S2 Fig *Pseudocleobis krausi* n. sp. vectors from Maury unpublished drawings of male specimen SMF 17374 from Travesía, Chile. (PDF)

S3 Fig *Pseudocleobis choros* n. sp. vectors from Maury unpublished drawings of male specimen SMF 17367 from El Tofo, Chile. (PDF)

S4 Fig *Pseudocleobis cekalovici* n. sp. vectors from Maury unpublished drawings and photo of male specimen (MCZ 170350) from Farellones, above Santiago, Chile. (PDF)

S1 Table Measurement used for the variability report of the new species. (PDF)
