# Supplementary material for: Unveiling high solifuge diversity: Review of the genus Pseudocleobis Pocock, 1900 (Ammotrechidae) in Chile with the description of nine new species
Source: PLoS One. 2025 Jan 15;20(1):e0309776. doi: 10.1371/journal.pone.0309776 (PMC11734978; doi:10.1371/journal.pone.0309776)
Supplement: S2 Fig — (PDF) [file pone.0309776.s002.pdf]

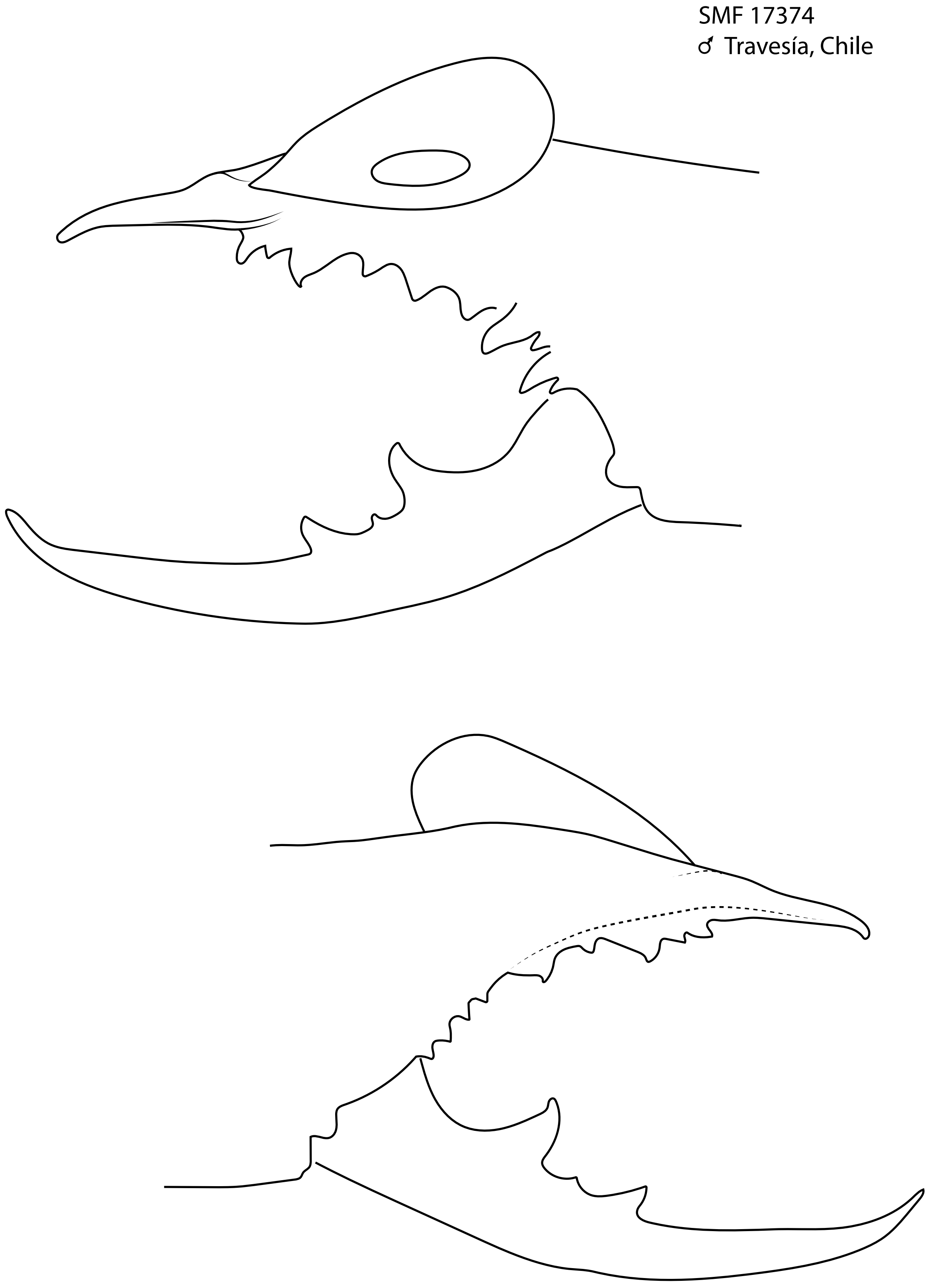

S2 Fig: *Pseudocleobis krausi* n. sp. vectors from Maury unpublished drawings of male specimen SMF 17374 from Travesía, Chile.
