# Supplementary material for: Unveiling high solifuge diversity: Review of the genus Pseudocleobis Pocock, 1900 (Ammotrechidae) in Chile with the description of nine new species
Source: PLoS One. 2025 Jan 15;20(1):e0309776. doi: 10.1371/journal.pone.0309776 (PMC11734978; doi:10.1371/journal.pone.0309776)
Supplement: S3 Fig — (PDF) [file pone.0309776.s003.pdf]

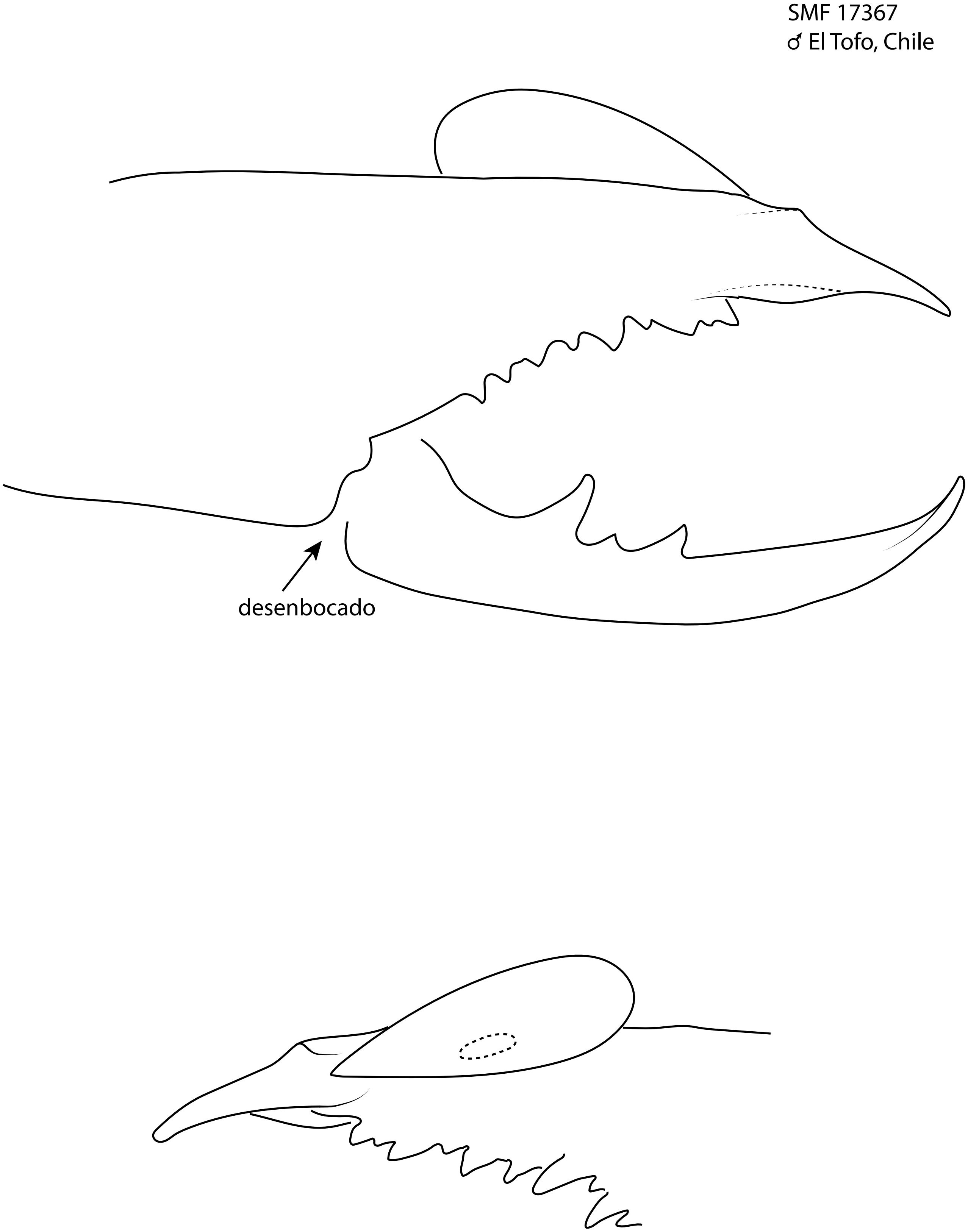

S3 Fig: *Pseudocleobis choros* n. sp. vectors from Maury unpublished drawings of male specimen SMF 17367 from El Tofo, Chile.
