# Supplementary material for: Unveiling high solifuge diversity: Review of the genus Pseudocleobis Pocock, 1900 (Ammotrechidae) in Chile with the description of nine new species
Source: PLoS One. 2025 Jan 15;20(1):e0309776. doi: 10.1371/journal.pone.0309776 (PMC11734978; doi:10.1371/journal.pone.0309776)
Supplement: S1 Table — (PDF) [file pone.0309776.s005.pdf]

		Pseudocleobis elongatus n. sp. (males)												
		1	2*	3**	4	5	6	7	8	9	10	MIN	MEAN	MAX
Body	Total Lenght w/o chel.	6,49	5,93	6,20	6,17	-	-	-	-	-	-	5,93	6,20	6,49
Propeltidium	Lenght	1,36	1,46	1,39	1,45	-	-	-	-	-	-	1,36	1,41	1,46
Propeltidium	Weight	1,77	1,76	1,50	1,74	-	-	-	-	-	-	1,50	1,69	1,77
Chelicera	Lenght	2,39	2,40	2,47	2,45	-	-	-	-	-	-	2,39	2,43	2,47
Chelicera	Weight	0,72	0,69	0,70	0,71	-	-	-	-	-	-	0,69	0,70	0,72

		Pseudocleobis elongatus n. sp. (females)												
		1	2*	3*	4	5	6	7	8	9	10	MIN	MEAN	MAX
Body	Total Lenght w/o chel.	7,51	10,43	6,79	8,59	-	-	-	-	-	-	6,79	8,33	10,43
Propeltidium	Lenght	1,42	1,78	1,42	1,38	-	-	-	-	-	-	1,38	1,50	1,78
Propeltidium	Weight	2,34	2,66	2,62	2,04	-	-	-	-	-	-	2,04	2,41	2,66
Chelicera	Lenght	3,54	4,61	3,72	3,29	-	-	-	-	-	-	3,29	3,79	4,61
Chelicera	Weight	1,10	1,48	1,18	0,91	-	-	-	-	-	-	0,91	1,17	1,48

		Pseudocleobis atacamensis n. sp. (males)												
		1*	2*	3**	4***	5	6	7	8	9	10	MIN	MEAN	MAX
Body	Total Lenght w/o chel.	7,47	10,04	9,01	9,11	10,60	-	-	-	-	-	7,47	9,25	10,60
Propeltidium	Lenght	1,80	2,11	2,07	1,70	1,90	-	-	-	-	-	1,70	1,92	2,11
Propeltidium	Weight	2,12	2,79	2,62	2,27	2,42	-	-	-	-	-	2,12	2,44	2,79
Chelicera	Lenght	3,14	3,97	3,43	3,18	3,60	-	-	-	-	-	3,14	3,47	3,97
Chelicera	Weight	0,93	1,14	1,07	0,90	0,98	-	-	-	-	-	0,90	1,00	1,14

		Pseudocleobis puna n. sp. (males)												
		1	2	3	4	5	6	7	8	9	10	MIN	MEAN	MAX
Body	Total Lenght w/o chel.	10,94	14,02	-	-	-	-	-	-	-	-	10,94	12,48	14,02
Propeltidium	Lenght	2,09	2,18	-	-	-	-	-	-	-	-	2,09	2,14	2,18
Propeltidium	Weight	2,69	2,77	-	-	-	-	-	-	-	-	2,69	2,73	2,77
Chelicera	Lenght	3,80	3,92	-	-	-	-	-	-	-	-	3,80	3,86	3,92
Chelicera	Weight	1,24	1,19	-	-	-	-	-	-	-	-	1,19	1,21	1,24

		Pseudocleobis choros n. sp. (females)												
		1	2	3	4	5	6	7	8	9	10	MIN	MEAN	MAX
Body	Total Lenght w/o chel.	10,79	10,50	11,70	-	-	-	-	-	-	-	10,50	11,00	11,70
Propeltidium	Lenght	1,79	1,80	1,48	-	-	-	-	-	-	-	1,48	1,69	1,80
Propeltidium	Weight	2,54	2,43	2,52	-	-	-	-	-	-	-	2,43	2,50	2,54
Chelicera	Lenght	3,68	3,76	3,81	-	-	-	-	-	-	-	3,68	3,75	3,81
Chelicera	Weight	1,24	1,17	1,14	-	-	-	-	-	-	-	1,14	1,18	1,24

		Pseudocleobis lalackama n. sp. (males)												
		1*	2*	3	4	5	6	7	8	9	10	MIN	MEAN	MAX
Body	Total Lenght w/o chel.	6,79	6,07	6,74	-	-	-	-	-	-	-	6,07	6,54	6,79
Propeltidium	Lenght	1,37	1,31	1,25	-	-	-	-	-	-	-	1,25	1,31	1,37
Propeltidium	Weight	1,72	1,55	1,84	-	-	-	-	-	-	-	1,55	1,71	1,84
Chelicera	Lenght	2,43	2,04	2,37	-	-	-	-	-	-	-	2,04	2,28	2,43
Chelicera	Weight	0,73	0,67	0,71	-	-	-	-	-	-	-	0,67	0,70	0,73

		Pseudocleobis mumai n. sp. (males)												
		1	2	3	4	5	6	7	8	9	10	MIN	MEAN	MAX
Body	Total Lenght w/o chel.	6,30	7,82	8,41	-	-	-	-	-	-	-	6,30	7,51	8,41
Propeltidium	Lenght	1,52	1,56	1,62	-	-	-	-	-	-	-	1,52	1,57	1,62
Propeltidium	Weight	1,88	1,72	1,95	-	-	-	-	-	-	-	1,72	1,85	1,95
Chelicera	Lenght	2,44	2,47	2,75	-	-	-	-	-	-	-	2,44	2,55	2,75
Chelicera	Weight	0,71	0,74	0,81	-	-	-	-	-	-	-	0,71	0,75	0,81

		Pseudocleobis cekalovici n. sp. (females)												
		1	2	3	4	5	6	7	8	9	10	MIN	MEAN	MAX
Body	Total Lenght w/o chel.	9,63	9,37	11,29	10,77	8,66	-	-	-	-	-	8,66	9,94	11,29
Propeltidium	Lenght	1,63	1,57	1,76	1,93	1,77	-	-	-	-	-	1,57	1,73	1,93
Propeltidium	Weight	2,42	2,69	2,70	2,74	2,74	-	-	-	-	-	2,42	2,66	2,74
Chelicera	Lenght	3,28	3,57	3,58	4,02	3,87	-	-	-	-	-	3,28	3,66	4,02
Chelicera	Weight	0,97	1,14	1,14	1,34	1,16	-	-	-	-	-	0,97	1,15	1,34

		Pseudocleobis cekalovici n. sp. (males)												
		1	2	3	4	5	6	7	8	9	10	MIN	MEAN	MAX
Body	Total Lenght w/o chel.	6,92	9,02	8,85	7,41	8,57	8,56	8,95	7,36	6,54	5,98	5,98	7,82	9,02
Propeltidium	Lenght	1,32	1,53	1,57	1,44	1,55	1,49	1,60	1,57	1,50	1,13	1,13	1,47	1,60
Propeltidium	Weight	1,90	2,09	2,03	2,04	2,05	2,04	2,11	1,82	1,71	1,59	1,59	1,94	2,11
Chelicera	Lenght	2,50	2,61	2,86	2,75	2,37	2,56	2,44	2,63	2,39	2,20	2,20	2,53	2,86
Chelicera	Weight	0,81	0,89	0,85	0,80	0,79	0,82	0,85	0,81	0,75	0,61	0,61	0,80	0,89

		Pseudocleobis escuadra n. sp. (males)												
		1	2	3	4	5	6	7	8	9	10	MIN	MEAN	MAX
Body	Total Lenght w/o chel.	8,57	9,67	9,09	9,31	7,79	9,54	-	-	-	-	7,79	8,99	9,67
Propeltidium	Lenght	1,69	1,59	1,68	1,70	2,03	1,71	-	-	-	-	1,59	1,73	2,03
Propeltidium	Weight	1,92	2,43	2,30	2,28	1,61	2,37	-	-	-	-	1,61	2,15	2,43
Chelicera	Lenght	2,61	3,23	2,94	3,02	2,91	3,33	-	-	-	-	2,61	3,01	3,33
Chelicera	Weight	0,84	0,94	0,97	0,94	0,89	0,95	-	-	-	-	0,84	0,92	0,97

		Pseudocleobis escuadra n. sp. (females)												
		1	2	3	4	5	6	7	8	9	10	MIN	MEAN	MAX
Body	Total Lenght w/o chel.	10,18	12,48	8,47	8,04	10,78	8,88	-	-	-	-	8,04	9,80	12,48
Propeltidium	Lenght	1,61	2,11	1,61	1,45	1,85	1,44	-	-	-	-	1,44	1,68	2,11
Propeltidium	Weight	2,81	3,31	2,82	2,27	2,72	2,25	-	-	-	-	2,25	2,70	3,31
Chelicera	Lenght	4,16	4,21	3,64	2,95	4,04	3,13	-	-	-	-	2,95	3,69	4,21
Chelicera	Weight	1,28	1,46	1,27	1,06	1,24	0,90	-	-	-	-	0,90	1,20	1,46
